# Infant outcome at four years of age after intrapartum sampling of scalp blood lactate for fetal assessment. A cohort study

**DOI:** 10.1371/journal.pone.0193887

**Published:** 2018-03-23

**Authors:** Nana Wiberg, Tobias Wirenfeldt Klausen, Tobias Tyrberg, Lennart Nordström, Eva Wiberg-Itzel

**Affiliations:** 1 Department of Obstetrics and Gynecology, Skåne University Hospital, Lund, Sweden; 2 Department of Clinical Sciences, Lund University, Malmö, Sweden; 3 Department of Hematology, Herlev University Hospital, Herlev, Denmark; 4 Department of Infectious Diseases, Sahlgrenska University Hospital, Goteborg, Sweden; 5 Department of Obstetrics and Gynecology, Karolinska University Hospital, Stockholm Sweden; 6 Department of Women’s and Children’s Health, Karolinska Institute, Stockholm, Sweden; 7 Department of Clinical Science and Education, Section of Obstetrics and Gynecology, Karolinska Institutet, Sodersjukhuset, Stockholm, Sweden; TNO, NETHERLANDS

## Abstract

**Objective:**

To correlate the value of lactate in fetal scalp blood at delivery and the outcomes of the offspring at four years of age.

**Methods:**

Cases where scalp blood lactate was taken within sixty minutes before delivery were identified from the randomized trial "Determination of pH or lactate in fetal scalp blood in management of intrapartum fetal distress”. Data were grouped according to the generally accepted cutoffs for normality, pre-acidemia, acidemia and concentrations above mean +2 SD during the second stage. The outcome measures included gross-/fine motor function, vision, hearing, speaking and cognitive disorders, signs of central motor damage and referral to specialized pediatric services.

**Results:**

307 cases were available for final analyse. With normal scalp lactate concentration, the number of children with a diagnosed disorder was lower compared to the pre-acidemic/acidemic groups, although the findings were only significant for fine motor dysfunction (p = 0.036). Elevated lactate values were significantly associated with increased risk for a poorer capacity of attention and understanding of instructions (OR 1.37, 95% CI 1.07–1.74), and for fine motor dysfunction (OR 1.22, 95% CI 1.00–1.49) at the age of four.

**Conclusion:**

Higher levels of lactate in fetal scalp blood seems to be associated with increased risk of an aberrant developmental outcome at four years of age in some areas.

## Introduction

The key purpose of fetal surveillance is to ensure fetal wellbeing. Interventions are performed with the aim of improving short- and long-term morbidity and neurological outcome. Nevertheless, long-term outcome is seldom reported of randomised controlled trials or prospective observational studies[1]. Follow-up studies are expensive, time-consuming, beyond obstetricians’ main research field, and—because of the timeframe—often out of the primary defined study period. Optimal fetal monitoring is characterized by low inter- and intra-observer variation, no false negative tests and a low false positive ratio to keep the rate of unnecessary interventions to a minimum. Worldwide, cardiotocography (CTG) is the most frequently used method[2]. CTG is characterized by a high intra- and inter-observer variation and a low positive predictive value for adverse outcome, resulting in a variable but inappropriately high operative delivery rate[3]. A few methods have been proposed as an accessory assessment to CTG, including fetal scalp blood sampling (FBS) to better decide whether to manage the case expectantly or intervene for better neonatal outcome[4,5]. Traditionally scalp pH has been measured, but since the introduction of point-of-care (POC) devices, analysis of lactate has become an alternative to pH[6]. It is impossible to discriminate between respiratory and metabolic acidemia from only pH, whereas lactate is the major end-product of anaerobic metabolism. It has been suggested that neonatal complications such as impaired neurologic development and end-organ damage are associated with metabolic rather than respiratory acidosis[7,8]. Studies have shown that lactate in scalp blood has better or equal predictive and preventive properties compared to pH in the identification of metabolic acidemia, low Apgar score and short-term neonatal morbidity with no significant difference in the rate of operative deliveries [9,10]. However, the long-term outcome for children managed by fetal scalp blood lactate sampling during labor is unknown.

The aim of this study was to correlate scalp lactate concentrations collected within 60 minutes before delivery with infant outcome at four years of age.

## Material and methods

This study is a retrospective cohort study. From December 2002 to December 2005, 3,007 women from 10 delivery units in Sweden with an indication for fetal scalp blood sampling were randomized to measurement of either scalp lactate or scalp pH (International Standard randomized trial Number 1606064)[10]. In total, 1,504 women were assigned to lactate analysis. Inclusion criteria in the RCT were: singleton pregnancy, cephalic presentation, gestational age ≥ 34 weeks and sustained suspicious or pathological CTG defined according to the Swedish national guidelines[11]. A commercially available point-of-care device, Lactate ProTM (Arkary, Kyoto, Japan), measured lactate with the result displayed after one minute. The cut-off points for lactate and recommendations for action were: <4.2mmol/L = normal value with no indication for intervention, 4.2–4.8mmol/L = pre-acidemia, repeat FBS within 20–30 minutes if non-reassuring CTG continues, >4.8mmol/L = acidemia and delivery should be considered[12]. The acid-base values in arterial and venous cord blood were analyzed within 15 minutes after delivery by a stationary blood gas analyzer.

From the RCT we included all cases with the latest scalp lactate sampling less than 60 minutes between FBS and delivery. The group division was based on the lactate cut-offs for normality, pre-acidemia, acidemia [12] and concentrations above mean + 2 SD during second stage [13,14] where the control group was defined as cases with normal lactate concentration. An informed consent request was sent to the parents for acquisition of information pertaining to the child’s performance at the four-year health examination. When informed consent was obtained, the respective school nurse was asked to send a copy of the medical record and to specify whether the child had been referred to specialized pediatric services or rehabilitation. A second reminder was sent within two months to both the parents and the school nurse if we did not receive the informed consent/requested medical record after the first letter.

Based on recommendations from the Swedish National Board of Health and Welfare, all children in Sweden are offered several examinations during childhood including an extensive examination at four years of age at Child Health Centers[15]. The examination is performed by specially educated health nurses or specialized doctors and includes tasks for testing cognitive, gross and fine motor function, vision, hearing and speaking levels. During the examination, the investigator evaluates the child’s capacity for attention and the ability to understand instructions. The information is noted in a standardized health chart and kept at the relevant Health Center until the child starts school, at which point the documents are sent to the child's school. The need for referral to specialized pediatric centers or, for example, speech therapy is recorded in the health chart.

### Statistical analyses

The ratios were compared and analysed using Chi-square or Fisher's exact test. Group comparison of continuous variables was performed with the Kruskal-Wallis test or the Mann-Whitney U test when appropriate. Values indicated as medians, interquartile range (IQR) The risk difference was calculated to compare the frequency of disability between the groups. Only if overall comparisons over more than two groups showed statistical significance step-down analysis was performed between pairs of categories either by Fisher’s exact test or Mann-Whitney test with correction for multiple tests. Binary logistic regression was used to measure the association between continuous lactate values and the probability of having a disability and presents with odds ratios (OR) with a 95% confidence interval. All tests were two-sided and a P-value < 0.05 was considered significant. Analyses were performed using IBM SPSS 22 (SPSS Inc. Chicago, Illinois, USA) and R version 3.2.3 /R Foundation for Statistical Computing, Vienna, Austria).

### Ethics

The primary RCT was approved by The Regional Ethic Committee of Karolinska Institute, Stockholm, Sweden (Diary number 109/02). A complimentary approval for the 4-year follow up study was given by The Regional Ethic Committee of Karolinska Institute, Stockholm, Sweden (Diary number 2013/015-32).

## Results

**Fig 1:**
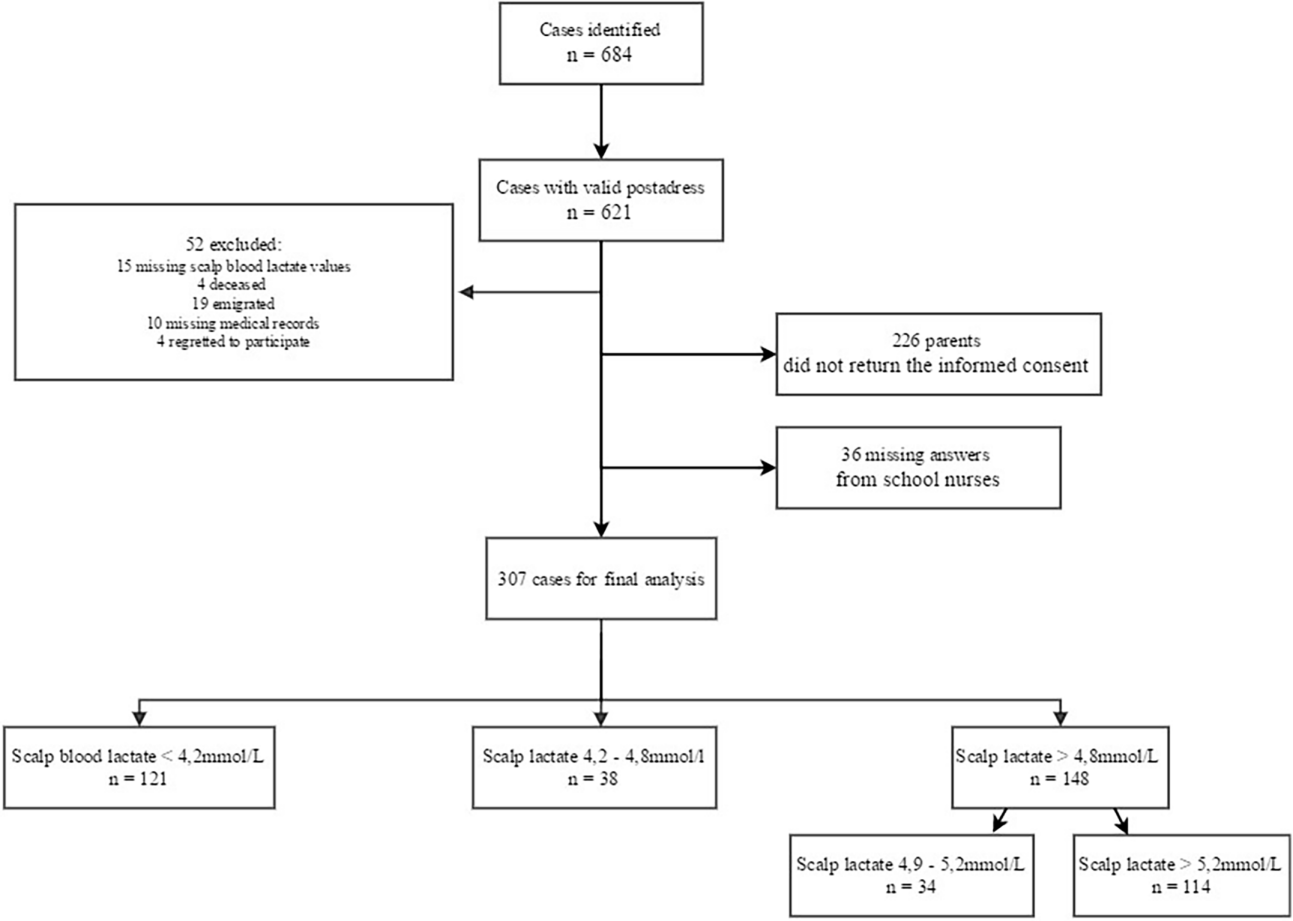
Flow chart demonstrating exclusions and group division based on the levels of scalp blood lactate.

From the RCT, 684 cases with attempted scalp blood lactate analysis less than 60 minutes before delivery were identified. Searching in the Swedish National personal register, it was possible to trace 621 of the 684 cases via a valid post address. After further exclusions, 307 cases were available for final analysis, Fig 1.

Table 1 shows the comparison of scalp lactate, blood-gas values in cord artery blood and neonatal outcome divided into the following categories: no answer from the parents, follow-up data received, lack of medical records from the school and primary excluded due to emigration, missing lactate values or death. There was significant differences between the groups in the level of scalp lactate (p<0.001), level of base deficit (p = 0.015) and Apgar score at five minutes (p = 0.021).

**Table 1:** Comparison of fetal scalp blood lactate, blood-gas values in umbilical cord artery blood, neonatal outcome and delivery mode. Values indicated as medians, interquartile range (IQR) and number of cases (percentages).

	*Group I* Informed consent from parents not obtained n = 226	*Group II* Follow-up data received n = 307	*Group III* Informed consent obtained but no answer from the school n = 36	*Group IV* Primary excluded n = 52	*P* value
	Median (IQR)	Median (IQR)	Median (IQR)	Median (IQR)	
Scalp lactate mmol/L	3.7 (2.6; 5.1)	4.8 (3.2; 5.9)	3.9 (2.6; 5.3)	4.9 (3.3; 6.0)	< 0.0001†
Missing	0	0	0	15	
Infant weight, g	3455 (3150; 3840)	3550 (3273; 3880)	3433 (3275; 3703)	3475 (3250; 3655)	0.12†
Missing	0	0	0	12	
Gestational age, days	283 (277; 290)	285 (279; 290)	283 (279; 288)	291 (284; 296)	0.040†
Missing	152	131	22	28	
Umbilical artery pH	7.24 (7.18; 7.28)	7.21 (7.15; 7.27)	7.21 (7.15; 7.30)	7.22 (7.14; 7.28)	0.20†
Missing	19	14	0	14	
Umbilical artery BD	1.35 (-5.00; 6.00)	4.00 (-4.60; 8.00)	-3.60 (-7.95; 5.00)	5.00 (-3.57; 7.00)	0.015†
Missing	20	18	1	14	
	n (%)	n (%)	n (%)	n (%)	
Metabolic acidemia^a^	6 (2.9)	7 (2.4)	1 (2.9)	1 (2.6)	0.94‡
Missing	16	15	1	14	
Apgar score < 7 at 5 min	5 (2.2)	18 (5.9)	1 (2.8)	5 (12.5)	0.021‡
Missing	0	0	0	12	
HIE I-II	0	3 (1.0)	0	1 (2.5)	0.19‡
Missing	0	0	0	11	
Mode of delivery: Normal delivery	35 (15.5)	85 (27.7)	6 (16.7)	11 (27.5)	0.004‡‡
Ventrose/forceps	153 (67.7)	152 (49.5)	24 (66.7)	21 (52.5)	
Emergency CS	18 (8.0)	40 (13.0)	3 (8.3)	7 (17.5)	
Non-emergency CS	20 (8.8)	30 (9.8)	3 (8.3)	1 (2.5)	
Missing	0	0	0	12	

BD: base deficit, HIE: hypoxic-ischemic encephalopathy, CS: cesarean section.

^a^pH <7,05 and BD >12 mmol/l in umbilical artery cord blood.

† Kruskal Wallis test.

‡ Fisher’s exact test.

‡‡Fisher exact test using Monte Carlo method.

In Table 2, the cut-off values for normality (control group), pre-acidemia and acidemia were used as references for sub-dividing neonatal data, certain acid-base values in umbilical artery blood and delivery mode. Across the three groups, we found significant differences in umbilical artery pH (p *=* 0.005), low AS (p = 0.002) and delivery mode. Step-down analysis for umbilical artery pH showed significant higher values for normal lactate compaired to lactate >4.8mmol/L but no differences between other groups (p = 0.001, p = 0.13 and p = 0.51). For Apgar score the step down analyses showed significant more participants having low AS in the lactate >4.8mmol/L group compaired to the two other groups (p = 0.003, p = 0.045) but no difference between these groups (p = 1). For delivery mode step down analyses showed differences between normal lactate and both groups with elevated lactate (p < 0.0001 for both) but not between the two elevated groups (p = 0.58).

**Table 2:** Characteristics of 307 cases with measurement of scalp blood lactate less than 60 minutes before delivery. Values are medians, interquartile range (IQR) and number of cases (percentages).

	Lactate < 4.2 mmol/l (n = 121)	Lactate 4.2–4.8 mmol/l (n = 38)	Lactate > 4.8 mmol/l (n = 148)	*P* value
	Median (IQR)	Median (IQR)	Median (IQR)	
Infant weight, g	3605 (3280; 3990)	3592 (3328; 3858)	3482 (3233; 3833)	0.19†
Umbilical artery pH	7.24 (7.17; 7.29)	7.21 (7.14; 7.25)	7.20 (7.14; 7.25)	0.005†
Umbilical artery BD	3.65 (-3.78; 6.95)	3.05 (-6.17; 7.85)	4.00 (-5.65; 8.00)	0.64†
	n (%)	n (%)	n (%)	n (%)
Gender, boys %	59 (48.8)	19 (50.0)	69 (46.6)	0.90^‡‡^
Metabolic acidosis^a^	1 (0.9)	0	6 (4.2)	0.19^‡^
Apgar score < 7 at 5 min	2 (1.7)	0	16 (10.8)	0.002^‡^
HIE I-II	0	0	3 (2.0)	0.36^‡^
Mode of delivery Normal delivery	2 (1.7)	14 (36.8)	69 (46.6)	≤ 0.0001^‡^
Ventrose/forceps	101 (83.4)	11 (28.9)	40 (27.0)	
Emergency CS	0	11 (28.9)	29 (19.6)	
Non-emergency CS	18 (14.9)	2 (5.3)	10 (6.8)	

BD: base deficit, HIE: hypoxic-ischemic encephalopathy, CS: cesarean section.

^a^pH <7,05 and BD >12 mmol/l in umbilical artery blood.

†Kruskal Wallis test.

^‡^Fisher’s exact test.

‡‡ Chi-square test

Table 3 shows the difference in outcomes at the 4 year examination between the different groups (normality (control group), pre-acidemia, acidemia and more than 2 SD above mean during the second stage of labor). All over the frequency of a dysfunction were lower in the control group although only significant for fine motor dysfunction (p = 0.030). Sub-analysis for this task showed: normal lactate versus lactate between 4.9–5.2mmol/l (p = 0.022) and normal lactate versus lactate > 5.2mmol/L (p = 0.031). Comparison of outcomes in the low and high range of acidemic scalp lactate values revealed a higher proportion of disorders in the 4.9 to 5.2mmol/L group (7/9 disorders) compared with those having had lactate concentrations above 5.2mmol/l,

**Table 3:** Results from the health investigation of 307 children. The different performance tasks are defined by the Swedish National Board of Health. Values are number of cases (%) of the whole study group. Risk differences with the corresponding 95% confidence intervals are provided for each task.

Scalp lactate mmol/L					
	Normal values = controls	Values predicting preacidemia, acidemia and > mean +2SD during second stage (>5.2mmol/L)			
	< 4.2 n = 121	4.2–4.8 n = 38	4.9–5.2 n = 34	> 5.2 n = 114	P
Gross motor dysfunction	2 (1.7%)	0 -1.7 (5.8;7.6)	3 (9.1%) 7.4 (0.2; 22.0)	6 (5.3%) 3.6 (-1.4; 9.5)	0.084†
Fine motor dysfunction	2 (1.7%)	2 (5.3%) 3.6 (-2.1; 15.7)	4 (11.8%) 10.1 (1.9; 25.0)	9 (7.9%) 6.2 (0.6; 12.8)	0.036†
Visual disorder	15 (12.5%)	6 (15.8%) 3.3 (-7.7; 18.7)	5 (15.2%) 2.7 (-8.7; 19.1)	19 (16.7%) 4.2 (-5.0; 13.4)	0.81†
Speech disorder	13 (10.8%)	4 (10.5%) -0.3 (-9.6; 14.9)	5 (15.2%) 4.3 (-6.6; 20.7)	9 (7.9%) -2.9 (-10.7; 0.8)	0.62†
Hearing disorder	5 (4.2%)	0 -4.2 (-9.5; 5.3)	2 (6.1%) 1.8 (-5.1; 15.6)	1 (0.9%) -3.4 (-8.7; 1.3)	0.14†
Capacity of attention and understanding instructions	2 (1.7%)	0 -1.7 (-6.0; 7.8)	1 (3.0%) 1.3 (-3.6; 13.7)	6 (5.3%) 3.6 (-1.6; 9.4)	0.32†
Cognitive disorder	4 (3.4%)	2 (5.3%) 1.9 (-4.4; 14.1)	3 (9.4%) 6.0 (-1.9; 21.0)	7 (6.1%) 2.8 (-3.1; 9.1)	0.46†
Signs of central motoric damage	1 (0.8%)	0 -0.8 (-4.6; 8.4)	2 (5.9%) 5.0 (-0.6; 18.3)	4 (3.5%) 2.7 (-1.7; 7.9)	0.17†
Referral to pediatric clinic (somatic/psychiatric)	6 (5%)	5 (13.2%) 8.1 (-1.1; 12.5)	6 (17.6%) 12.6 (1.8; 28.7)	12 (10.5%) 5.5 (-1.6; 13.0)	0.080†
Composite of data	31 (25.6%)	13 (34.2%) 8.6 (-6.9; 25.9)	12 (35.3%) 9.7 (-6.5; 7.8)	36 (31.6%) 6.0 (-5.5; 17.3)	0.57‡

†Fisher’s exact test.

‡Chi-square test

Calculating odds ratios (OR), we found an univariate significantly increased risk of decreased capacity of attention and understanding instructions and for fine motor function with increased scalp lactate concentrations (OR 1.37; 95% CI 1.07–1.74, p = 0.011), respective (OR 1.22; 95% CI 1.00–1.49, p = 0.050). There were no significantly increased odds for other outcomes with increasing concentrations of scalp lactate (Table 4).

**Table 4:** Odds ratio for having a disability at 4 years of age with increased level of lactate in fetal scalp blood during labor measured by Lactate Pro^TM^.

	R^2^	OR (95% CI)	P
Gross motor dysfunction	0.032	1.23 (0.97–1.55)	0.086
Fine motor dysfunction	0.033	1.22 (1–1.49)	0.050
Visual disorder	0.016	1.13 (0.98–1.3)	0.088
Speech disorder	0.00	1 (0.84–1.2)	0.97
Hearing disorder	0.04	0.91 (0.64/1.31)	0.62
Capacity of attention and to understand instructions	0.08	1.37 (1.07–1.74)	0.011
Cognitive disorder	0.013	1.14 (0.92–1.41)	0.23
Signs of central motoric damage	0.041	1.27 (0.96–1.67)	0.089
Referral to pediatric clinic (somatic/psychiatric)	0.023	1.17 (0.99–1.38)	0.065
Composite of outcome	0.011	1.08 (0.98–1.23)	0.12

R^2^ (Nagelkerke), P *<* 0.05 significant

## Discussion

In this work we have, for the first time, been studying long-term effects in children born with high/low lactate levels in scalp blood during their delivery. Children having had higher levels of scalp lactate had an increased probability of fine motoric and cognitive dysfunction at four years of age, while children with low levels of lactate at delivery did not appear to have this kind of trouble.

We know that high levels of scalp lactate during labor have an association with poor fetal outcome after delivery, with an affected Apgar score, increased risk for metabolic acidosis and transfer to NICU [9,10,16] and that gestational adjusted lactate in arterial umbilical cord blood has the best accuracy for depressed vitality a birth (low 5-minute AS or transfer to NICU) [17]. The association between the neonatal condition and the long-term outcome is disputed although it seems that the like-hood of persistent sequelae rises with worsened acidosis, but still, a reliable tool to identify permanent brain damage remains elusive [7,18]. Off cause we cannot exclude influence of other factors on our results such as ante partum events, severe neonatal infection, environmental pollution or social-economical status but we find it acceptable to assume that the probability of such influences should be equally/consistent distributed throughout the study groups with equal or no impact on the results.

One of the major purposes of fetal surveillance has been to reduce the amount of asphyxia-related cerebral palsy (CP). Though formerly believed to be the main cause of instances of CP, studies show that birth-related asphyxia only accounts for a minority of cases of CP [19,20]. Continuous FHR monitoring is by far the most common method of surveillance for fetal well-being during labor despite that CTG host a significant inter/intra observer variation as well as a low specificity although a high sensitivity for fetal acidosis by a non-reassuring tracing. Therefore, the use of CTG alone leads to a higher frequency of operative deliveries due to the high false positive rate. As a consequence, obstetricians have over years discussed and studied complementary methods where FBS is one such method[3,12]. The argumentation is the ability to increase the diagnostic specificity for injurious hypoxia when the FHR recordings shift from a reassuring to a non-reassuring trace during delivery. FBS is simply, well tolerated by the woman with the result available in only a few minutes and a false negative test result is unlikely [4,10,13,21]. Obstetric emergencies such as acute severe chorioamnionitis, cord prolapse, total placental abruption or uterine scar rupture might happen at any time and are not within the scope of FBS; such emergencies need to be managed immediately.

We were able to identify two RCTs comparing fetal monitoring with CTG alone and CTG combined with FBS, one published and one ongoing [21,22]. The published study from 1979 found a tendency towards the reduction of caesarean sections when FBS was added, although the finding was not significant. Several other indirect measures and some retrospective observational studies support the conclusion of a reduction in operative deliveries when FBS is used complementary to CTG Avoiding instrumental delivery without compromising the fetus will reduce the health risks associated with both vaginal and abdominal instrumental delivery for both the mother and child as well as decrease the economic burden especially related to caesarean section. From our results, we suggest that labor can continue although of a pathological CTG tracing if scalp lactate is with the normal range. Although of the relative small material, our results are clinically important and one can speculate that with a larger study differences would be more sustainable and seen in other areas.

During delivery, it is a physiological factor that the fetus suffers from a mild intermittent hypoxia due to uterine contractions, resulting in low pH and high pCO_2_ (respiratory acidemia). Sustained oxygen deficiency can develop into metabolic acidemia characterized by low pH and high lactate. In high concentrations, both the hydrogen ions and lactate ions are injurious to the fetal brain. To a certain extent, the hydrogen ions can be buffered in fetal compartments (extra-and intracellular) whereas the lactate ions have to be transported actively over the placenta membrane, commonly a slow process. It is not possible to separate respiratory and metabolic acidemia by pH alone, thus scalp lactate is suggested to be preferred to pH to assess the degree of potential malicious hypoxia[10,23]. An elevated level of scalp blood lactate can be regarded as an early marker in the hypoxic process of intrapartum hypoxia[24] and a normal scalp lactate during the course of labor is reassuring for a healthy, vigorous fetus, allowing labor to continue.

Hypoxia might also be chronic, established in the antenatal period or early in labor, before admission to the labor ward. In these cases, the outcome for the neonate is dependent upon the duration and severity of the hypoxia, two parameters that influence outcome. Etiologies unrelated to asphyxia, such as maternal/fetal infection and inflammation, have a clear association with both HIE and later CP[25]. We cannot exclude the possibility that the high lactate concentration in some of the fetuses was due to an intra-uterine event long before FBS and was not induced close to the time FBS was performed. In these cases, lactate acidemia could only be predictive for birth acidemia and could not have a preventive role. The natural limitation of FBS and similar diagnostic tests is that they provide a spot value. One could argue that obtaining multiple scalp samples in a clinical setting could resemble continuous monitoring comparable to CTG.

It is crucial that a test checking for fetal wellbeing has no false negative results, i.e. that fetuses with a normal scalp blood test is well oxygenated and not acidemic. In the initial report of our RCT we explored those having had a scalp blood sampling within 60 minutes of delivery and none of the fetuses with normal scalp blood lactate were delivered with severe birth acidemia in umbilical cord blood, defined as cord artery pH < 7.00 [10].

The calculation of statistical power with the current number of follow-up participants and on the assumption that the prevalence of motor skill disorders and/or cognitive delay at four years is between 10 to 15% [26] in a normal population showed that the difference in adverse outcome between the non-acidemic and acidemic groups was at least at a relative risk (RR) of 2.3 with a power at 80%, i.e. a risk difference (RD) of 13% to reach significance. This was not in fact the case for most of the tasks presumptive due to the relative low number of cases. The present follow-up data suggest that morbidity at four years of age is low and that its prevalence was similar to the general population for the control group [26–29].

The use of scalp blood sampling varies widely between countries. When the method was launched in the 1960s, pH was analysed. However, a large volume of blood was and is still needed and the analysis procedure is complicated and time-consuming, implicating that some 10–20% of sampling events fail and in some 9% of the cases the delay between sampling and result exceeded the accepted timeframe[10,30–32]. The introduction of POC-lactate-test-strip methods removed these difficulties and made lactate analysis more attractive. There are several lactate meters on the market and due to the test characteristics and variations within the POCs it is important to emphasize that the recommended cut-off values for normality/ pre-acidemia/acidemia are based on measurements with only Lactate Pro^TM^ [33]

Strengths: This is the first long-term follow-up study of fetuses monitored by intrapartum scalp blood sampling as a supplementary test to confirm or exclude the diagnosis of hypoxia/acidosis in case of CTG abnormalities. The Swedish national screening program at four years of age is a standardised and extensive examination of the child, enabling detection of rather mild impairments

Limitations: The number of “lost to follow up” was high since we had difficulties of identifying valid post addresses and because in many cases the informed consent was not returned.

## Supporting information

S1 File Neonatal and 4 year follow-up dataset. Dataset: https://www.protocols.io/view/infant-outcome-at-four-years-of-age-after-intrapar-k3ecyje.
(PDF)
