# Supplementary material for: Infant outcome at four years of age after intrapartum sampling of scalp blood lactate for fetal assessment. A cohort study
Source: PLoS One. 2018 Mar 23;13(3):e0193887. doi: 10.1371/journal.pone.0193887 (PMC5865709; doi:10.1371/journal.pone.0193887)
Supplement: S1 File — Dataset: https://www.protocols.io/view/infant-outcome-at-four-years-of-age-after-intrapar-k3ecyje. (PDF) [file pone.0193887.s001.pdf]

| Pat nummer | 1 ingen svar fra | Sista prov lac | Tid sista prov- | Paritet | Fostervikt | gestations |
|---|---|---|---|---|---|---|
| 330 | 1 | 4,0 | 00:51 | 1 | 3220 | |
| 34 | 1 | 2,8 | 00:10 | 1 | 3300 | |
| 420 | 1 | 2,3 | 00:45 | 3 | 2545 | |
| 230 | 1 | 1,7 | 00:40 | 1 | 3865 | |
| 2554 | 1 | 6,7 | 00:35 | 0 | 3615 | 290 |
| 2042 | 1 | 3,9 | 00:38 | 1 | 2830 | |
| 550 | 1 | 5,1 | 00:43 | 0 | 2475 | 276 |
| 198 | 1 | 4,4 | 00:18 | 0 | 3160 | 278 |
| 1526 | 1 | 6,1 | 00:04 | 1 | 4115 | 296 |
| 2210 | 1 | 1,6 | 00:33 | 2 | 2670 | |
| 1013 | 1 | 3,1 | 00:38 | 2 | 3450 | |
| 1486 | 1 | 2,1 | 00:23 | 5 | 3275 | |
| 289 | 1 | 2,4 | 00:57 | 1 | 3140 | |
| 2439 | 1 | 2,7 | 00:43 | 2 | 3410 | |
| 93 | 1 | 6,6 | 00:18 | 0 | 3675 | 296 |
| 2328 | 1 | 1,6 | 00:19 | 1 | 3615 | |
| 2565 | 1 | 7,8 | 00:25 | 0 | 3975 | 301 |
| 6 | 1 | 2,3 | 00:19 | 4 | 4020 | |
| 243 | 1 | 4,0 | 00:17 | 1 | 3210 | |
| 2044 | 1 | 3,1 | 00:24 | 2 | 3340 | |
| 2654 | 1 | 2,1 | 00:29 | 1 | 3730 | |
| 2450 | 1 | 6,2 | 00:29 | 0 | 3780 | 288 |
| 528 | 1 | 2,4 | 00:17 | 3 | 3900 | |
| 426 | 1 | 3,4 | 00:52 | 1 | 3495 | |
| 1207 | 1 | 5,6 | 00:12 | 1 | 4800 | 285 |
| 526 | 1 | 7,9 | 00:27 | 0 | 3220 | 292 |
| 2922 | 1 | 5,1 | 00:26 | 0 | 3415 | 286 |
| 2384 | 1 | 2,7 | 00:13 | 1 | 4630 | |
| 1993 | 1 | 1,9 | 00:45 | 1 | 2745 | |
| 1680 | 1 | 0,8 | 00:57 | 1 | 3645 | |
| 1674 | 1 | 1,9 | 00:48 | 4 | 4270 | |
| 2025 | 1 | 3,1 | 00:25 | 2 | 4000 | |
| 1978 | 1 | 4,9 | 00:17 | 1 | 3790 | 268 |
| 968 | 1 | 4,9 | 00:14 | 0 | 3320 | 295 |
| 2017 | 1 | 3,8 | 00:42 | 1 | 3255 | |
| 476 | 1 | 3,3 | 00:52 | 2 | 3520 | |
| 1375 | 1 | 4,3 | 00:30 | 1 | 3800 | 278 |
| 1955 | 1 | 2,7 | 00:50 | 3 | 3300 | |
| 1335 | 1 | 1,9 | 00:53 | 2 | 4010 | |
| 2416 | 1 | 2,7 | 00:08 | 1 | 4560 | |
| 2270 | 1 | 3,7 | 00:53 | 2 | 3030 | |
| 1331 | 1 | 4,2 | 00:13 | 2 | 3845 | |
| 2656 | 1 | 2,6 | 00:19 | 3 | 2840 | |
| 2588 | 1 | 4,0 | 00:47 | 4 | 2280 | |
| 2143 | 1 | 2,8 | 00:59 | 1 | 3225 | |
| 2499 | 1 | 2,1 | 00:30 | 1 | 3450 | |
| 304 | 1 | 6,9 | 00:12 | 1 | 3060 | 265 |
| 2827 | 1 | 4,6 | 00:14 | 0 | 4110 | 273 |
| 1775 | 1 | 6,8 | 00:14 | 0 | 3820 | 284 |
| 946 | 1 | 3,2 | 00:13 | 2 | 3625 | |
| 2030 | 1 | 3,0 | 00:20 | 2 | 3690 | |
| 982 | 1 | 4,7 | 00:24 | 2 | 4605 | |
| 2363 | 1 | 2,1 | 00:40 | 1 | 3650 | |
| 1339 | 1 | 3,0 | 00:22 | 1 | 3790 | |
| 2776 | 1 | 5,2 | 00:12 | 1 | 4365 | 281 |
| 2489 | 1 | 6,9 | 00:11 | 1 | 2945 | 282 |
| 557 | 1 | 1,7 | 00:56 | 2 | 3200 | |
| 1139 | 1 | 4,3 | 00:19 | 0 | 2935 | 296 |

| | | | | | | |
|---|---|---|---|---|---|---|
| 994 | 1 | 4,6 | 00:46 | 1 | 3790 | 275 |
| 1499 | 1 | 3,1 | 00:10 | 1 | 2350 | |
| 42 | 1 | 3,9 | 00:15 | 1 | 4070 | |
| 1706 | 1 | 3,2 | 00:43 | 1 | 3180 | |
| 2974 | 1 | 3,8 | 04:38 | 1 | 3250 | |
| 2101 | 1 | 1,2 | 00:39 | 1 | 3605 | |
| 1655 | 1 | 5,1 | 00:10 | 1 | 3185 | 293 |
| 2254 | 1 | 3,7 | 00:21 | 1 | 3045 | |
| 2165 | 1 | 2,2 | 00:52 | 1 | 3470 | |
| 2934 | 1 | 3,2 | 00:36 | 1 | 3460 | |
| 64 | 1 | 2,0 | 00:22 | 1 | 3565 | |
| 211 | 1 | 1,6 | 00:57 | 1 | 4220 | |
| 406 | 1 | 4,7 | 00:43 | 1 | 3050 | 278 |
| 2826 | 1 | 6,2 | 00:15 | 1 | 3600 | 290 |
| 2237 | 1 | 2,2 | 00:29 | 2 | 3840 | |
| 2980 | 1 | 5,7 | 00:16 | 0 | 3280 | 288 |
| 1601 | 1 | 2,4 | 00:39 | 1 | 2790 | |
| 1074 | 1 | 5,6 | 00:35 | 0 | 3365 | 285 |
| 502 | 1 | 4,3 | 00:23 | 0 | 3360 | 284 |
| 967 | 1 | 5,4 | 00:12 | 0 | 3550 | 283 |
| 2638 | 1 | 2,0 | 05:18 | 1 | 4180 | |
| 390 | 1 | 1,4 | 01:00 | 1 | 3135 | |
| 438 | 1 | 3,8 | 00:53 | 1 | 3525 | |
| 1683 | 1 | 6,8 | 00:21 | 0 | 3235 | 274 |
| 1194 | 1 | 1,3 | 00:17 | 2 | 3480 | |
| 1228 | 1 | 3,8 | 00:19 | 1 | 3880 | |
| 22 | 1 | 4,8 | 00:20 | 0 | 3945 | 279 |
| 2275 | 1 | 5 | 00:38 | 0 | 3400 | 297 |
| 108 | 1 | 2,0 | 00:41 | 1 | 2940 | |
| 240 | 1 | 3,0 | 00:27 | 1 | 4100 | |
| 1703 | 1 | 8 | 00:19 | 0 | 3430 | 293 |
| 1186 | 1 | 4,8 | 00:38 | 0 | 3110 | 291 |
| 1871 | 1 | 3,9 | 00:24 | 1 | 2860 | |
| 2528 | 1 | 2,6 | 00:11 | 2 | 1990 | |
| 1906 | 1 | 2,6 | 00:18 | 1 | 3210 | |
| 332 | 1 | 4,1 | 00:45 | 1 | 3640 | |
| 1185 | 1 | 4,8 | 00:29 | 0 | 3370 | 293 |
| 2169 | 1 | 5,2 | 00:20 | 1 | 2468 | |
| 2955 | 1 | 6,8 | 00:15 | 0 | 3295 | 280 |
| 1529 | 1 | 1,7 | 00:50 | 2 | 3180 | |
| 1103 | 1 | 3,9 | 00:41 | 1 | 3350 | |
| 1055 | 1 | 6,0 | 00:31 | 1 | 3095 | |
| 976 | 1 | 2,3 | 00:18 | 1 | 4400 | |
| 1366 | 1 | 5,4 | 00:27 | 0 | 2815 | 280 |
| 318 | 1 | 4,0 | 00:21 | 1 | 3700 | |
| 1032 | 1 | 4,2 | 00:12 | 1 | 4050 | |
| 1931 | 1 | 8,1 | 00:50 | 1 | 3364 | |
| 2029 | 1 | 3,8 | 00:09 | 1 | 3750 | |
| 1051 | 1 | 2,8 | 00:12 | 1 | 3645 | |
| 2718 | 1 | 5,7 | 00:25 | 0 | 3800 | 272 |
| 1532 | 1 | 4,1 | 00:37 | 3 | 3480 | |
| 52 | 1 | 3,0 | 00:55 | 1 | 2710 | |
| 2513 | 1 | 1,1 | 00:56 | 1 | 3840 | |
| 1989 | 1 | 1,4 | 00:19 | 1 | 4355 | |
| 1960 | 1 | 3,7 | 00:38 | 1 | 3285 | |
| 1263 | 1 | 3,1 | 00:03 | 2 | 3020 | |
| 2511 | 1 | 4,9 | 00:03 | 0 | 2790 | 267 |
| 2003 | 1 | 1,6 | 00:51 | 2 | 3085 | |
| 1351 | 1 | 7,7 | 00:12 | 0 | 3040 | 282 |

| | | | | | | |
|---|---|---|---|---|---|---|
| 186 | 1 | 14,6 | 00:51 | 0 | 2540 | 281 |
| 848 | 1 | 4,6 | 00:30 | 1 | 3300 | |
| 2540 | 1 | 5,2 | 00:11 | 0 | 4340 | 284 |
| 1806 | 1 | 5,6 | 00:49 | 1 | 3170 | |
| 922 | 1 | 2,0 | 00:45 | 1 | 2755 | |
| 2847 | 1 | 2,8 | 00:12 | 1 | 2510 | |
| 952 | 1 | 2,7 | 01:57 | 1 | 4330 | |
| 2211 | 1 | 1,7 | 00:18 | 1 | 4095 | |
| 2247 | 1 | 5,6 | 00:15 | 0 | 3410 | 287 |
| 1329 | 1 | 2,8 | 00:53 | 1 | 3195 | |
| 2305 | 1 | 5,4 | 00:55 | 0 | 3900 | 258 |
| 2420 | 1 | 3,1 | 00:33 | 1 | 3290 | |
| 1590 | 1 | 8,1 | 00:20 | 0 | 3015 | 264 |
| 294 | 1 | 6,4 | 00:09 | 1 | 3258 | |
| 2819 | 1 | 2,2 | 00:33 | 1 | 3570 | |
| 1109 | 1 | 1,9 | 05:59 | 1 | 3735 | |
| 1914 | 1 | 3,7 | 00:19 | 2 | 3200 | |
| 1987 | 1 | 3,4 | 00:34 | 1 | 3865 | |
| 3008 | 1 | 3,9 | 00:24 | 1 | 3520 | |
| 2127 | 1 | 2,3 | 01:28 | 1 | 4065 | |
| 2721 | 1 | 6,6 | 00:08 | 1 | 3385 | 283 |
| 2877 | 1 | 1,6 | 00:24 | 5 | 3385 | |
| 1855 | 1 | 3,3 | 00:15 | 2 | 3520 | |
| 594 | 1 | 1,6 | 00:53 | 2 | 3990 | |
| 2525 | 1 | 11,4 | 00:08 | 0 | 3150 | 270 |
| 2353 | 1 | 3,6 | 00:25 | 1 | 2840 | |
| 1979 | 1 | 4,0 | 00:14 | 1 | 2245 | |
| 1355 | 1 | 4,0 | 00:10 | 1 | 3335 | |
| 2875 | 1 | 6,7 | 00:26 | 0 | 3435 | 277 |
| 586 | 1 | 1,1 | 00:12 | 1 | 3160 | |
| 190 | 1 | 2,3 | 00:03 | 3 | 3870 | |
| 2381 | 1 | 3,4 | 00:52 | 1 | 4000 | |
| 2081 | 1 | 4,0 | 00:35 | 1 | 2440 | |
| 1845 | 1 | 4,2 | 00:43 | 1 | 4490 | 278 |
| 1097 | 1 | 2,7 | 00:34 | 1 | 3980 | |
| 934 | 1 | 2,7 | 00:59 | 1 | 4325 | |
| 1389 | 1 | 4,8 | 00:35 | 0 | 3950 | 282 |
| 2299 | 1 | 2,0 | 00:07 | 1 | 3470 | |
| 1518 | 1 | 4,2 | 00:33 | 0 | 3510 | 263 |
| 1999 | 1 | 4,1 | 00:25 | 1 | 3980 | |
| 2446 | 1 | 6,2 | 00:12 | 0 | 3620 | 291 |
| 2924 | 1 | 2,7 | 00:19 | 1 | 3260 | |
| 2490 | 1 | 8,8 | 00:34 | 0 | 2095 | 278 |
| 2646 | 1 | 3,4 | 00:56 | 1 | 4020 | |
| 995 | 1 | 3,4 | 00:41 | 2 | 2825 | |
| 2806 | 1 | 6,6 | 00:41 | 0 | 4260 | 294 |
| 2482 | 1 | 2,1 | 00:12 | 3 | 3735 | |
| 1068 | 1 | 2,9 | 00:50 | 1 | 3285 | |
| 1023 | 1 | 2,1 | 00:15 | 1 | 2945 | |
| 44 | 1 | 6,9 | 00:22 | 0 | 2745 | 283 |
| 2640 | 1 | 3,6 | 00:36 | 1 | 3335 | |
| 563 | 1 | 5,3 | 00:23 | 0 | 3885 | 293 |
| 142 | 1 | 1,7 | 01:40 | 1 | 2850 | |
| 2992 | 1 | 2,8 | 00:12 | 3 | 3790 | |
| 280 | 1 | 6 | 00:20 | 1 | 3475 | 277 |
| 1446 | 1 | 7,8 | 00:21 | 0 | 3200 | 294 |
| 301 | 1 | 2,0 | 00:38 | 1 | 3730 | |
| 1746 | 1 | 2,0 | 00:27 | 1 | 3570 | |
| 1704 | 1 | 6,5 | 00:15 | 0 | 3430 | 289 |

| | | | | | | |
|---|---|---|---|---|---|---|
| 448 | 1 | 4,0 | 00:31 | 1 | 3060 | |
| 1995 | 1 | 2,6 | 00:57 | 1 | 3790 | |
| 2303 | 1 | 2,3 | 00:58 | 1 | 3160 | |
| 1402 | 1 | 4,2 | 00:32 | 1 | 3645 | 274 |
| 541 | 1 | 1,8 | 00:48 | 1 | 2740 | |
| 1215 | 1 | 4,1 | 00:27 | 0 | 3800 | 285 |
| 849 | 1 | 2,8 | 00:44 | 1 | 3630 | |
| 1599 | 1 | 3,1 | 00:37 | 1 | 3360 | |
| 2172 | 1 | 5,9 | 00:18 | 0 | 3915 | 295 |
| 396 | 1 | 3,6 | 00:55 | 1 | 2940 | |
| 1154 | 1 | 2,7 | 00:20 | 1 | 3660 | |
| 2181 | 1 | 3,9 | 00:32 | 1 | 4160 | |
| 1050 | 1 | 7,3 | 00:06 | 0 | 3420 | 277 |
| 14 | 1 | 2,9 | 00:05 | 1 | 4575 | |
| 2954 | 1 | 5,4 | 00:48 | 0 | 3210 | 285 |
| 322 | 1 | 3,7 | 00:18 | 2 | 2755 | |
| 2229 | 1 | 2,9 | 01:00 | 2 | 3820 | |
| 2990 | 1 | 5,8 | 00:39 | 0 | 2985 | 265 |
| 932 | 1 | 5,3 | 00:33 | 0 | 2155 | 264 |
| 2815 | 1 | 5,1 | 00:31 | 0 | 3860 | 283 |
| 1514 | 1 | 4,2 | 00:30 | 0 | 4185 | 284 |
| 986 | 1 | 2,2 | 00:55 | 1 | 4005 | |
| 2816 | 1 | 3,8 | 00:07 | 1 | 3640 | |
| 1330 | 1 | 5,3 | 00:14 | 0 | 3680 | 288 |
| 1418 | 1 | 5,9 | 00:40 | 0 | 2630 | 266 |
| 1534 | 1 | 5,2 | 00:12 | 1 | 4065 | 282 |
| 58 | 1 | 3,3 | 00:37 | 1 | 3575 | |
| 416 | 1 | 2,6 | 01:00 | 1 | 4255 | |
| 1935 | 1 | 3,1 | 00:58 | 1 | 3965 | |
| 2648 | 1 | 4,1 | 00:32 | 2 | 2520 | |
| 1251 | 1 | 4,0 | 00:52 | 1 | 3960 | |
| 2458 | 1 | 2,1 | 00:13 | 1 | 3970 | |
| 1383 | 1 | 3,7 | 00:13 | 1 | 3165 | |
| 1628 | 1 | 2,9 | 00:56 | 1 | 3820 | |
| 282 | 1 | 3,4 | 00:37 | 1 | 3555 | |
| 960 | 1 | 2,2 | 01:00 | 1 | 3115 | |
| 1713 | 1 | 2,9 | 00:43 | 1 | 3030 | |
| 1288 | 1 | 2,2 | 00:23 | 1 | 3150 | |
| 2008 | 1 | 5,4 | 00:10 | 1 | 3005 | |
| 1167 | 1 | 2,7 | 00:35 | 1 | 3660 | |
| 2027 | 1 | 4,1 | 00:35 | 1 | 4445 | |
| 1585 | 1 | 2,8 | 00:47 | 1 | 3520 | |
| 1437 | 1 | 3,3 | 00:18 | 1 | 3895 | |
| 2055 | 1 | 3,3 | 00:40 | 1 | 3380 | |
| 2478 | 1 | 3,7 | 00:29 | 1 | 3030 | |
| 2426 | 1 | 7,7 | 00:15 | 0 | 4305 | 285 |
| 2530 | 1 | 8,3 | 00:45 | 0 | 2960 | 279 |
| 2636 | 1 | 5,9 | 00:30 | 0 | 3315 | 287 |
| 1183 | 1 | 5,1 | 00:34 | 0 | 3125 | 264 |
| 2996 | 2 | 7,9 | 00:22 | 0 | 1905 | 230 |
| 1156 | 2 | 2,2 | 00:39 | 2 | 3185 | |
| 2602 | 2 | 8,6 | 00:25 | 0 | 3755 | 290 |
| 2671 | 2 | 5,4 | 00:18 | 0 | 4205 | 298 |
| 1260 | 2 | 4,3 | 00:08 | 1 | 3475 | 285 |
| 2246 | 2 | 5,4 | 00:04 | 1 | 2970 | 285 |
| 2473 | 2 | 3,4 | 00:10 | 4 | 3699 | |
| 1064 | 2 | 5,0 | 00:14 | 2 | 3765 | |
| 84 | 2 | 0,9 | 00:50 | 3 | 3840 | |
| 2605 | 2 | 4,7 | 00:14 | 0 | 3005 | 286 |

| | | | | | | |
|---|---|---|---|---|---|---|
| 250 | 2 | 4,0 | 00:34 | 1 | 3355 | |
| 1722 | 2 | 2,6 | 00:57 | 1 | 3755 | |
| 1027 | 2 | 3,2 | 00:49 | 3 | 3975 | |
| 238 | 2 | 4,4 | 00:41 | 0 | 3350 | 282 |
| 2748 | 2 | 4,3 | 00:18 | 2 | 2370 | |
| 1965 | 2 | 6,2 | 00:24 | 1 | 3320 | 277 |
| 26 | 2 | 2,0 | 00:12 | 1 | 3605 | |
| 1458 | 2 | 7,6 | 00:17 | 1 | 3370 | 291 |
| 1912 | 2 | 2,3 | 00:08 | 3 | 3705 | |
| 216 | 2 | 4,1 | 00:37 | 2 | 4500 | |
| 2032 | 2 | 3,0 | 00:25 | 1 | 4000 | |
| 2236 | 2 | 6,7 | 00:28 | 0 | 2815 | 288 |
| 2831 | 2 | 5,6 | 00:54 | 0 | 2910 | 274 |
| 358 | 2 | 4,1 | 00:27 | 1 | 4050 | |
| 2475 | 2 | 9 | 00:28 | 1 | 3635 | 283 |
| 1755 | 2 | 3,9 | 00:28 | 1 | 4130 | |
| 899 | 2 | 7,1 | 00:13 | 0 | 3410 | 287 |
| 2716 | 2 | 7,6 | 00:32 | 0 | 4135 | 295 |
| 525 | 2 | 4,1 | 00:50 | 1 | 3610 | |
| 2889 | 2 | 7,6 | 00:11 | 0 | 3305 | 281 |
| 1456 | 2 | 2,3 | 00:30 | 1 | 3795 | |
| 2106 | 2 | 5,3 | 00:15 | 0 | 3860 | 290 |
| 992 | 2 | 5,6 | 00:14 | 0 | 3685 | 294 |
| 20 | 2 | 2,1 | 00:49 | 2 | 3945 | |
| 1810 | 2 | 5,6 | 00:37 | 0 | 3380 | 276 |
| 1785 | 2 | 5,4 | 00:28 | 0 | 3238 | 292 |
| 1115 | 2 | 1,1 | 00:20 | 1 | 4110 | |
| 2387 | 2 | 5,1 | 00:42 | 0 | 4156 | 286 |
| 1259 | 2 | 6,3 | 00:21 | 0 | 3430 | 288 |
| 2292 | 2 | 3,8 | 00:40 | 1 | 4860 | |
| 175 | 2 | 3,4 | 00:30 | 1 | 3470 | |
| 1445 | 2 | 7,4 | 00:22 | 0 | 2615 | 259 |
| 858 | 2 | 3,9 | 00:23 | 2 | 3270 | |
| 167 | 2 | 9 | 00:28 | 0 | 3780 | 287 |
| 1717 | 2 | 5 | 00:39 | 0 | 4260 | 282 |
| 112 | 2 | 3,1 | 00:18 | 2 | 3865 | |
| 472 | 2 | 4,8 | 00:21 | 0 | 3450 | 279 |
| 2739 | 2 | 5,1 | 00:06 | 0 | 2750 | 282 |
| 2 | 2 | 1,3 | 00:41 | 1 | 4990 | |
| 2585 | 2 | 4,8 | 00:16 | 0 | 4320 | 286 |
| 2802 | 2 | 3,9 | 00:55 | 1 | 3380 | |
| 1146 | 2 | 6 | 00:15 | 0 | 3480 | 294 |
| 2590 | 2 | 2,6 | 00:43 | 1 | 2610 | |
| 772 | 2 | 5 | 00:21 | 0 | 3515 | 280 |
| 1574 | 2 | 3,8 | 00:51 | 1 | 3330 | |
| 2338 | 2 | 11,2 | 00:10 | 0 | 3220 | 277 |
| 1647 | 2 | 3,8 | 00:44 | 1 | 3350 | |
| 996 | 2 | 5,6 | 00:13 | 0 | 3355 | 290 |
| 1512 | 2 | 5,4 | 00:17 | 0 | 3888 | 291 |
| 412 | 2 | 5,4 | 00:15 | 0 | 3365 | 300 |
| 1398 | 2 | 2,3 | 00:30 | 1 | 3335 | |
| 148 | 2 | 2,6 | 00:28 | 3 | 3155 | |
| 24 | 2 | 2,8 | 00:59 | 1 | 4125 | |
| 1527 | 2 | 5,9 | 00:18 | 0 | 3140 | 290 |
| 546 | 2 | 1,9 | 00:21 | 1 | 2710 | |
| 1953 | 2 | 1,2 | 00:48 | 3 | 3600 | |
| 265 | 2 | 4,2 | 00:45 | 0 | 3040 | 272 |
| 653 | 2 | 2,1 | 04:49 | 2 | 3695 | |
| 2915 | 2 | 4,9 | 00:21 | 1 | 3590 | 276 |

| | | | | | | |
|---|---|---|---|---|---|---|
| 2526 | 2 | 4,2 | 00:38 | 1 | 4030 | 282 |
| 609 | 2 | 2,2 | 05:07 | 1 | 3330 | |
| 2273 | 2 | 5,1 | 00:37 | 0 | 3200 | 279 |
| 1287 | 2 | 1,9 | 00:25 | 2 | 4190 | |
| 374 | 2 | 6,6 | 00:13 | 1 | 3475 | 288 |
| 1070 | 2 | 4,7 | 00:06 | 0 | 3615 | 273 |
| 136 | 2 | 1,7 | 00:51 | 1 | 3430 | |
| 28 | 2 | 8,2 | 00:28 | 1 | 4105 | 289 |
| 1044 | 2 | 10,4 | 00:29 | 1 | 3430 | 285 |
| 1731 | 2 | 5,1 | 00:08 | 0 | 2710 | 267 |
| 931 | 2 | 3,1 | 00:59 | 1 | 3545 | |
| 1742 | 2 | 2,3 | 00:35 | 1 | 4390 | |
| 1892 | 2 | 5,4 | 00:12 | 0 | 3840 | 282 |
| 308 | 2 | 4,6 | 00:47 | 0 | 3585 | 281 |
| 2321 | 2 | 4,6 | 00:51 | 1 | 3600 | |
| 2911 | 2 | 3,2 | 00:45 | 2 | 3995 | |
| 1811 | 2 | 6,2 | 00:20 | 0 | 3470 | 295 |
| 1903 | 2 | 2,3 | 00:36 | 3 | 4360 | |
| 2628 | 2 | 10 | 00:15 | 2 | 4440 | |
| 1918 | 2 | 7 | 00:15 | 0 | 3270 | 280 |
| 2660 | 2 | 5,1 | 00:19 | 1 | 3440 | 293 |
| 2285 | 2 | 4,8 | 00:38 | 1 | 3650 | 290 |
| 2199 | 2 | 2,6 | 00:33 | 2 | 4340 | |
| 904 | 2 | 5,8 | 00:17 | 1 | 3445 | |
| 2452 | 2 | 9 | 00:13 | 0 | 3395 | 282 |
| 1536 | 2 | 2,0 | 00:55 | 3 | 4660 | |
| 888 | 2 | 4,6 | 00:16 | 0 | 3760 | 284 |
| 2427 | 2 | 3,2 | 00:13 | 1 | 3280 | |
| 1369 | 2 | 3,6 | 00:22 | 1 | 3765 | |
| 2019 | 2 | 5,6 | 00:25 | 0 | 4980 | 300 |
| 1293 | 2 | 4,9 | 00:36 | 1 | 3830 | 287 |
| 1985 | 2 | 2,8 | 00:32 | 1 | 3155 | |
| 360 | 2 | 6,4 | 00:08 | 0 | 3430 | 258 |
| 2171 | 2 | 4,8 | 00:11 | 0 | 3350 | 274 |
| 871 | 2 | 8,3 | 00:31 | 0 | 3850 | 278 |
| 1794 | 2 | 2,4 | 00:53 | 1 | 3480 | |
| 1299 | 2 | 4,1 | 00:33 | 1 | 4230 | |
| 1066 | 2 | 3,8 | 00:35 | 1 | 2330 | |
| 1739 | 2 | 2,7 | 00:35 | 1 | 3765 | |
| 1827 | 2 | 7,3 | 00:15 | 0 | 3250 | 283 |
| 969 | 2 | 6,7 | 00:05 | 0 | 3040 | 285 |
| 1093 | 2 | 4,7 | 00:16 | 1 | 3610 | 291 |
| 2197 | 2 | 4,4 | 00:22 | 0 | 4650 | 291 |
| 293 | 2 | 4,4 | 00:26 | 1 | 4125 | 287 |
| 588 | 2 | 6 | 00:11 | 2 | 4530 | |
| 1454 | 2 | 3,9 | 00:31 | 1 | 3550 | |
| 2829 | 2 | 1,8 | 00:23 | 1 | 2910 | |
| 1776 | 2 | 2,6 | 00:36 | 1 | 4050 | |
| 2946 | 2 | 7,7 | 00:18 | 0 | 4035 | 296 |
| 2336 | 2 | 6,3 | 00:15 | 0 | 3485 | 291 |
| 2657 | 2 | 2,2 | 00:19 | 1 | 2505 | |
| 2235 | 2 | 5,2 | 00:26 | 0 | 3455 | 290 |
| 1312 | 2 | 9,9 | 00:26 | 0 | 3300 | 272 |
| 463 | 2 | 3,1 | 00:41 | 1 | 4080 | |
| 2594 | 2 | 3,7 | 00:17 | 1 | 3410 | |
| 86 | 2 | 2,1 | 00:42 | 2 | 3750 | |
| 974 | 2 | 6 | 00:16 | 0 | 3600 | 279 |
| 962 | 2 | 1,6 | 00:40 | 1 | 3850 | |
| 1126 | 2 | 3,7 | 00:23 | 3 | 3980 | |

| | | | | | | |
|---|---|---|---|---|---|---|
| 3004 | 2 | 5,8 | 00:28 | 0 | 3410 | 287 |
| 1838 | 2 | 5,1 | 00:15 | 1 | 3480 | |
| 1724 | 2 | 1,6 | 00:36 | 2 | 3735 | |
| 878 | 2 | 2,9 | 01:00 | 1 | 3185 | |
| 2593 | 2 | 9,6 | 00:15 | 0 | 3430 | 283 |
| 1614 | 2 | 3,6 | 00:10 | 1 | 2960 | |
| 1387 | 2 | 6,9 | 00:17 | 0 | 4130 | 295 |
| 2941 | 2 | 1,7 | 01:00 | 2 | 4920 | |
| 1667 | 2 | 2,2 | 00:07 | 1 | 3645 | |
| 270 | 2 | 3,3 | 00:15 | 1 | 3635 | |
| 100 | 2 | 3,6 | 00:13 | 1 | 3510 | |
| 1553 | 2 | 6,6 | 00:12 | 1 | 3805 | 293 |
| 1170 | 2 | 5,6 | 00:05 | 0 | 3540 | 299 |
| 425 | 2 | 5,6 | 00:14 | 0 | 3605 | 285 |
| 2508 | 2 | 2,9 | 00:55 | 1 | 3705 | |
| 94 | 2 | 7,6 | 00:27 | 1 | 4075 | 269 |
| 870 | 2 | 1,7 | 00:48 | 2 | 3560 | |
| 1843 | 2 | 6,1 | 00:11 | 0 | 2910 | 279 |
| 1039 | 2 | 8,4 | 00:23 | 0 | 2860 | 253 |
| 2393 | 2 | 3,9 | 00:17 | 2 | 3280 | |
| 1803 | 2 | 4,3 | 00:37 | 1 | 3955 | |
| 2225 | 2 | 4,2 | 00:17 | 0 | 3880 | 282 |
| 2296 | 2 | 5,6 | 00:13 | 1 | 3985 | 283 |
| 542 | 2 | 6,3 | 00:14 | 0 | 3120 | 287 |
| 2621 | 2 | 5,2 | 00:19 | 0 | 2750 | 283 |
| 1857 | 2 | 5,8 | 00:09 | 0 | 3480 | 277 |
| 1941 | 2 | 4,9 | 00:20 | 1 | 3880 | 269 |
| 2778 | 2 | 6,3 | 00:13 | 0 | 2720 | 287 |
| 868 | 2 | 6,1 | 00:16 | 0 | 3880 | 295 |
| 1271 | 2 | 3,1 | 00:33 | 1 | 3685 | |
| 380 | 2 | 5,1 | 00:27 | 0 | 3024 | 277 |
| 1971 | 2 | 6,6 | 00:26 | 0 | 3515 | 292 |
| 2821 | 2 | 4,1 | 00:15 | 1 | 3530 | |
| 1096 | 2 | 2,1 | 00:57 | 1 | 3280 | |
| 1640 | 2 | 5,4 | 00:13 | 0 | 3150 | 283 |
| 2556 | 2 | 5,1 | 00:42 | 0 | 3115 | 286 |
| 150 | 2 | 5 | 00:30 | 0 | 3500 | 293 |
| 859 | 2 | 3,2 | 00:43 | 1 | 3905 | |
| 1262 | 2 | 5,6 | 00:30 | 1 | 4000 | |
| 196 | 2 | 4,2 | 00:59 | 0 | 3690 | 277 |
| 877 | 2 | 1,8 | 00:28 | 1 | 3200 | |
| 512 | 2 | 5,4 | 00:29 | 0 | 3645 | 301 |
| 590 | 2 | 5,3 | 00:16 | 0 | 3245 | 299 |
| 2711 | 2 | 5,2 | 00:35 | 0 | 3065 | 251 |
| 110 | 2 | 3,9 | 00:52 | 1 | 2880 | |
| 2203 | 2 | 2,8 | 00:15 | 1 | 3810 | |
| 1881 | 2 | 5,2 | 00:38 | 0 | 3820 | 297 |
| 2334 | 2 | 4,9 | 00:11 | 1 | 3760 | 279 |
| 1513 | 2 | 4,9 | 00:56 | 0 | 2450 | 267 |
| 2613 | 2 | 2,8 | 00:23 | 2 | 4040 | |
| 1551 | 2 | 4,8 | 00:09 | 1 | 3790 | 268 |
| 2674 | 2 | 5,7 | 00:25 | 0 | 3960 | 290 |
| 1684 | 2 | 2,1 | 00:45 | 3 | 2830 | |
| 1782 | 2 | 6,6 | 00:47 | 1 | 4095 | 293 |
| 2758 | 2 | 2,2 | 00:13 | 1 | 4045 | |
| 1225 | 2 | 2,0 | 00:07 | 2 | 3620 | |
| 680 | 2 | 3,6 | 01:10 | 1 | 3275 | |
| 424 | 2 | 5,9 | 00:13 | 0 | 4240 | 294 |
| 2754 | 2 | 6 | 00:14 | 0 | 3790 | 279 |

| | | | | | | |
|---|---|---|---|---|---|---|
| 1750 | 2 | 3,9 | 00:41 | 1 | 3350 | |
| 1983 | 2 | 5,8 | 00:22 | 0 | 3615 | 255 |
| 2703 | 2 | 8,3 | 00:21 | 0 | 3040 | 285 |
| 633 | 2 | 4,6 | 00:40 | 0 | 4165 | 281 |
| 1285 | 2 | 4,9 | 00:11 | 0 | 2590 | 273 |
| 427 | 2 | 1,7 | 00:50 | 2 | 3180 | |
| 2998 | 2 | 5,2 | 00:26 | 0 | 4660 | 288 |
| 2116 | 2 | 2,9 | 00:24 | 1 | 3980 | |
| 2372 | 2 | 7,9 | 00:15 | 0 | 4010 | 295 |
| 1622 | 2 | 3,8 | 00:34 | 1 | 3845 | |
| 1466 | 2 | 4,9 | 00:16 | 0 | 3320 | 272 |
| 1980 | 2 | 4,3 | 00:25 | 0 | 2545 | 276 |
| 2702 | 2 | 6,7 | 00:34 | 1 | 3545 | 279 |
| 2226 | 2 | 5,3 | 00:40 | 0 | 3785 | 278 |
| 166 | 2 | 4,1 | 00:23 | 1 | 2965 | |
| 1419 | 2 | 4,8 | 00:15 | 0 | 3375 | 295 |
| 2083 | 2 | 4,6 | 00:13 | 0 | 3370 | 273 |
| 200 | 2 | 6,6 | 00:17 | 0 | 3975 | 290 |
| 2238 | 2 | 5,8 | 00:50 | 0 | 4175 | 284 |
| 372 | 2 | 4,2 | 00:11 | 0 | 2895 | 292 |
| 419 | 2 | 6 | 00:16 | 0 | 4055 | 297 |
| 487 | 2 | 7,9 | 00:12 | 0 | 3770 | 294 |
| 2346 | 2 | 5,2 | 00:31 | 0 | 3990 | 283 |
| 1945 | 2 | 6,4 | 00:28 | 0 | 3405 | 283 |
| 78 | 2 | 5,3 | 00:21 | 1 | 3510 | 261 |
| 2066 | 2 | 3,4 | 00:39 | 1 | 3000 | |
| 2600 | 2 | 4,8 | 00:33 | 0 | 2995 | 295 |
| 2294 | 2 | 3,3 | 00:51 | 1 | 4300 | |
| 1907 | 2 | 1,7 | 00:38 | 1 | 3675 | |
| 1092 | 2 | 2,6 | 00:42 | 1 | 3415 | |
| 2159 | 2 | 2,8 | 00:08 | 1 | 3485 | |
| 2264 | 2 | 8,1 | 00:14 | 0 | 3880 | 283 |
| 2405 | 2 | 2,8 | 00:19 | 1 | 3480 | |
| 2205 | 2 | 7 | 00:15 | 0 | 3120 | 281 |
| 1767 | 2 | 5,4 | 00:50 | 1 | 4030 | 288 |
| 1381 | 2 | 5,8 | 00:13 | 0 | 3660 | 284 |
| 381 | 2 | 4,1 | 00:39 | 1 | 4245 | |
| 2495 | 2 | 4,6 | 00:19 | 0 | 3890 | 295 |
| 898 | 2 | 7,2 | 00:16 | 0 | 3775 | 285 |
| 860 | 2 | 1,6 | 00:53 | 1 | 4320 | |
| 2414 | 2 | 1,8 | 00:50 | 2 | 4175 | |
| 2015 | 2 | 11,8 | 00:14 | 0 | 2760 | 286 |
| 1448 | 2 | 5,2 | 00:18 | 0 | 3535 | 292 |
| 1319 | 2 | 6,9 | 00:28 | 0 | 3250 | 283 |
| 2057 | 2 | 2,7 | 00:38 | 1 | 3550 | |
| 1831 | 2 | 7,9 | 00:17 | 0 | 3650 | 294 |
| 942 | 2 | 4,9 | 00:12 | 0 | 3070 | 284 |
| 46 | 2 | 4,0 | 00:43 | 1 | 3280 | |
| 1147 | 2 | 2,2 | 00:18 | 2 | 4570 | |
| 2733 | 2 | 7,1 | 00:13 | 0 | 3080 | 268 |
| 1524 | 2 | 5,7 | 00:22 | 0 | 3565 | 287 |
| 2949 | 2 | 1,9 | 00:30 | 2 | 3450 | |
| 2093 | 2 | 2,9 | 00:45 | 1 | 3990 | |
| 1787 | 2 | 2,9 | 00:34 | 1 | 3555 | |
| 1063 | 2 | 7,4 | 00:10 | 0 | 3295 | 288 |
| 1733 | 2 | 5,8 | 00:16 | 0 | 3545 | 295 |
| 2507 | 2 | 3,2 | 00:34 | 1 | 3840 | |
| 2396 | 2 | 3,6 | 00:44 | 1 | 3580 | |
| 2730 | 2 | 2,1 | 00:29 | 1 | 4020 | |

| | | | | | | |
|---|---|---|---|---|---|---|
| 1568 | 2 | 15,9 | 00:09 | 0 | 3475 | 275 |
| 2152 | 2 | 4,3 | 00:33 | 0 | 2420 | 273 |
| 2770 | 2 | 7,4 | 00:07 | 0 | 4365 | 288 |
| 2056 | 2 | 4,6 | 00:19 | 0 | 3710 | 285 |
| 2538 | 2 | 7,7 | 00:32 | 0 | 4460 | 284 |
| 1740 | 2 | 4,1 | 00:25 | 1 | 3710 | |
| 2317 | 2 | 6,3 | 00:11 | 0 | 3470 | 275 |
| 2550 | 2 | 4,9 | 00:59 | 0 | 3642 | 287 |
| 2187 | 2 | 2,6 | 00:34 | 2 | 4090 | |
| 2768 | 2 | 5,3 | 00:34 | 0 | 3200 | 294 |
| 1140 | 2 | 5 | 00:37 | 0 | 2980 | 274 |
| 2614 | 2 | 7,4 | 00:09 | 0 | 3310 | 281 |
| 313 | 2 | 4,9 | 00:23 | 0 | 4430 | 292 |
| 1566 | 2 | 4,4 | 00:11 | 0 | 3070 | 280 |
| 1451 | 2 | 5,9 | 00:17 | 0 | 3325 | 289 |
| 572 | 2 | 4,0 | 00:25 | 1 | 2944 | |
| 2846 | 2 | 6,7 | 00:32 | 0 | 3645 | 295 |
| 2972 | 2 | 6,3 | 00:09 | 0 | 3295 | 295 |
| 551 | 2 | 5,1 | 00:10 | 0 | 2990 | 287 |
| 2871 | 2 | 4,3 | 00:10 | 0 | 3950 | 288 |
| 1148 | 2 | 3,9 | 00:15 | 1 | 3450 | 276 |
| 978 | 2 | 2,4 | 00:56 | 1 | 3260 | |
| 2117 | 2 | 2,9 | 00:28 | 1 | 3270 | |
| 1487 | 2 | 4,4 | 00:42 | 0 | 3600 | 292 |
| 2189 | 2 | 5,6 | 00:12 | 0 | 3600 | 277 |
| 1649 | 2 | 7 | 00:33 | 0 | 3790 | 282 |
| 1507 | 2 | 11 | 00:12 | 0 | 2165 | 262 |
| 350 | 2 | 7,1 | 00:14 | 1 | 3180 | |
| 2973 | 2 | 6,6 | 00:12 | 0 | 4900 | 287 |
| 1779 | 2 | 2,2 | 01:00 | 1 | 4205 | |
| 534 | 2 | 4,8 | 00:30 | 0 | 3570 | 289 |
| 1060 | 2 | 5 | 00:19 | 0 | 3225 | 285 |
| 2177 | 2 | 2,9 | 00:57 | 1 | 3570 | |
| 2071 | 2 | 3,8 | 00:17 | 1 | 3430 | |
| 1498 | 2 | 4,8 | 00:39 | 0 | 3320 | 293 |
| 1342 | 2 | 5,9 | 00:42 | 0 | 3045 | 290 |
| 123 | 2 | 3,7 | 00:20 | 1 | 3185 | |
| 1701 | 2 | 5,2 | 00:39 | 0 | 3215 | 276 |
| 2063 | 2 | 6,7 | 00:15 | 1 | 3565 | |
| 959 | 2 | 3,2 | 00:13 | 2 | 3625 | |
| 2394 | 2 | 4,7 | 00:21 | 0 | 4560 | 285 |
| 1812 | 2 | 6 | 00:11 | 0 | 3065 | 284 |
| 1624 | 2 | 3,8 | 00:28 | 1 | 4515 | |
| 2131 | 2 | 2,0 | 00:19 | 1 | 2990 | |
| 2318 | 2 | 1,6 | 00:44 | 1 | 4680 | |
| 1106 | 2 | 1,6 | 00:54 | 1 | 3420 | |
| 2335 | 2 | 4,7 | 00:52 | 0 | 3565 | 280 |
| 2959 | 2 | 5,6 | 00:54 | 0 | 3445 | 274 |
| 1318 | 2 | 9,4 | 00:17 | 0 | 3225 | 267 |
| 138 | 2 | 2,2 | 00:45 | 1 | 3040 | |
| 1732 | 2 | 5,2 | 00:11 | 0 | 3965 | 281 |
| 1915 | 2 | 5,8 | 00:40 | 0 | 3890 | 265 |
| 1264 | 2 | 5,6 | 00:14 | 0 | 4020 | 277 |
| 582 | 2 | 3,1 | 00:06 | 1 | 2930 | |
| 2145 | 2 | 5,4 | 00:15 | 0 | 3595 | 280 |
| 2604 | 2 | 3,0 | 00:54 | 1 | 3276 | |
| 1494 | 2 | 7,0 | 00:46 | 1 | 3235 | 279 |
| 2097 | 2 | 2,6 | 00:46 | 1 | 3500 | |
| 2982 | 2 | 4,8 | 00:14 | 0 | 1860 | 259 |

| | | | | | | |
|---|---|---|---|---|---|---|
| 2870 | 2 | 3,4 | 00:42 | 1 | 2780 | |
| 2545 | 2 | 6,6 | 00:05 | 0 | 3580 | 287 |
| 180 | 3 | 2,4 | 00:52 | 1 | 2690 | |
| 286 | 3 | 2,6 | 00:57 | 1 | 3550 | |
| 1846 | 3 | 2,2 | 00:29 | 1 | 3310 | |
| 1357 | 3 | 3,9 | 00:33 | 1 | 4170 | |
| 226 | 3 | 2,7 | 00:33 | 1 | 3450 | |
| 207 | 3 | 5,8 | 00:39 | 1 | 3345 | 275 |
| 273 | 3 | 4,9 | 00:30 | 0 | 3550 | 280 |
| 1464 | 3 | 2,4 | 00:37 | 3 | 3505 | |
| 522 | 3 | 2,6 | 00:19 | 1 | 3010 | |
| 2040 | 3 | 3,9 | 00:38 | 2 | 3320 | |
| 2694 | 3 | 3,4 | 00:50 | 1 | 3400 | |
| 1715 | 3 | 1,3 | 00:23 | 4 | 4310 | |
| 1773 | 3 | 4,0 | 00:43 | 1 | 2640 | |
| 1404 | 3 | 6,1 | 00:22 | 1 | 3415 | 279 |
| 1289 | 3 | 6,1 | 00:09 | 1 | 4010 | 289 |
| 1162 | 3 | 4 | 00:49 | 3 | 3720 | |
| 296 | 3 | 2,7 | 00:56 | 1 | 3280 | |
| 1545 | 3 | 5,4 | 00:19 | 0 | 3385 | 293 |
| 2201 | 3 | 4,4 | 00:12 | 1 | 3475 | 280 |
| 16 | 3 | 5,9 | 00:07 | 0 | 3700 | 277 |
| 2176 | 3 | 2,1 | 00:14 | 1 | 4570 | |
| 2764 | 3 | 7,3 | 00:16 | 0 | 3270 | 286 |
| 2110 | 3 | 2,2 | 00:55 | 1 | 3705 | |
| 1181 | 3 | 6,0 | 00:15 | 1 | 3480 | |
| 2726 | 3 | 4,4 | 00:27 | 0 | 3404 | 286 |
| 1083 | 3 | 3,4 | 00:36 | 1 | 2645 | |
| 518 | 3 | 3,9 | | 1 | 4135 | |
| 1406 | 3 | 6,6 | 00:13 | 1 | 2910 | 267 |
| 371 | 3 | 2,4 | 00:41 | 1 | 2050 | |
| 2774 | 3 | 3,7 | 00:50 | 2 | 4365 | |
| 1851 | 3 | 4,4 | 00:07 | 0 | 3375 | 281 |
| 1017 | 3 | 2,9 | 00:51 | 1 | 4040 | |
| 2868 | 3 | 5,1 | 00:34 | 0 | 3225 | 287 |
| 1643 | 3 | 2,2 | 00:36 | 1 | 3605 | |
| 2918 | 3 | 6 | 00:25 | 0 | 3120 | 288 |
| 2744 | 3 | 4,9 | 00:40 | 1 | 3560 | 295 |
| 1199 | 4 | 6 | 00:08 | 1 | 4030 | 294 |
| 116 | 4 | 2,7 | 00:14 | 1 | 3240 | |
| 1152 | 4 | 5,0 | 00:15 | 2 | 3450 | |
| 1944 | 4 | 8,6 | 00:11 | 0 | 3535 | 293 |
| 2710 | 4 | 4,1 | 00:25 | 1 | 2635 | |
| 1099 | 4 | 1,3 | 00:53 | 1 | 3620 | |
| 2320 | 4 | 3,3 | 00:52 | 2 | 3520 | |
| 2796 | 4 | 4,2 | 00:33 | 0 | 3825 | 292 |
| 2620 | 4 | 4,0 | 00:39 | 4 | 3470 | |
| 1947 | 4 | 3,2 | 00:20 | 1 | 3265 | |
| 2517 | 4 | 5,6 | 00:30 | 0 | 3480 | 284 |
| 539 | 4 | 4,4 | 00:11 | 1 | 3540 | 288 |
| 1594 | 4 | 3,9 | 00:42 | 1 | 2200 | |
| 2581 | 4 | 2,1 | 00:42 | 2 | 3400 | |
| 1829 | 4 | 6,1 | 00:40 | 1 | 4245 | 299 |
| 861 | 4 | | | | | |
| 2419 | 4 | 5,7 | 00:21 | 0 | 3490 | 287 |
| 145 | 4 | | | | | |
| 2400 | 4 | 5,7 | 00:08 | 0 | 3490 | 276 |
| 1565 | 4 | 6,4 | 00:18 | 0 | 3285 | 284 |
| 961 | 4 | | | | | |

| | | | | | | |
|---|---|---|---|---|---|---|
| 2903 | 4 | 5,2 | 00:14 | 2 | 4120 | |
| 248 | 4 | 14,7 | 00:17 | 0 | 4190 | 291 |
| 1371 | 4 | 4,9 | 00:34 | 0 | 3835 | 295 |
| 2154 | 4 | 5,9 | 00:09 | 0 | 3520 | 291 |
| 1249 | 4 | | | | | |
| 2365 | 4 | 3,3 | 00:31 | 1 | 3395 | |
| 1353 | 4 | 6,6 | 00:16 | 0 | 3680 | 282 |
| 1422 | 4 | | | | | |
| 2279 | 4 | 7 | 00:25 | 0 | 2595 | 264 |
| 1227 | 4 | | | | | |
| 2547 | 4 | 5,3 | 00:19 | 0 | 3400 | 300 |
| 152 | 4 | 6,3 | 00:23 | 0 | 2755 | 271 |
| 2345 | 4 | 2,0 | 00:34 | 1 | 4050 | |
| 2107 | 4 | 6,4 | 00:23 | 0 | 3260 | 288 |
| 351 | 4 | | | | | |
| 1191 | 4 | 2,1 | 00:28 | 1 | 3160 | |
| 918 | 4 | 4,3 | 00:37 | 0 | 3930 | 296 |
| 1337 | 4 | 4,4 | 00:33 | 0 | 3150 | 296 |
| 2354 | 4 | 1,8 | 01:00 | 1 | 2650 | |
| 279 | 4 | | | | | |
| 1780 | 4 | | | | | |
| 1436 | 4 | 2,2 | 00:24 | 3 | 3080 | |
| 1809 | 4 | 6,4 | 00:09 | 0 | 3345 | 275 |
| 1865 | 4 | 5,2 | 00:14 | 0 | 3080 | 279 |
| 549 | 4 | 0,8 | 00:28 | 1 | 3630 | |
| 547 | 4 | | | | | |
| 1330 | 4 | | | | | |
| 2916 | 4 | | | | | |
| 2628 | 4 | | 00:15 | 1 | 4440 | 299 |
| 2067 | 4 | | 00:37 | 0 | 3440 | 297 |
| 2873 | 4 | | 00:48 | 0 | 3600 | 295 |

| Apgar1 | Apgar5 | Apgar10 | Apgar<7 vid 5 | Sex, | Förlossningssätt, | simpleforlossningss |
|---|---|---|---|---|---|---|
| 9 | 10 | 10 | 0 | P | 4 | 3 |
| 9 | 10 | 10 | 0 | P | 1 | 1 |
| 9 | 10 | 10 | 0 | P | 1 | 1 |
| 9 | 10 | 10 | 0 | P | 1 | 1 |
| 9 | 10 | 10 | 0 | 1 | 0 | 0 |
| 9 | 10 | 10 | 0 | P | 4 | 3 |
| 6 | 8 | 10 | 0 | 1 | 1 | 1 |
| 9 | 10 | 10 | 0 | 0 | 0 | 0 |
| 7 | 8 | 10 | 0 | 1 | 3 | 2 |
| 10 | 10 | 10 | 0 | F | 1 | 1 |
| 8 | 9 | 10 | 0 | P | 1 | 1 |
| 9 | 10 | 10 | 0 | P | 1 | 1 |
| 9 | 10 | 10 | 0 | F | 1 | 1 |
| 9 | 10 | 10 | 0 | F | 1 | 1 |
| 7 | 10 | 10 | 0 | 0 | 4 | 3 |
| 9 | 10 | 10 | 0 | F | 1 | 1 |
| 8 | 8 | 8 | 0 | 1 | 3 | 2 |
| 9 | 10 | 10 | 0 | P | 1 | 1 |
| 4 | 8 | 8 | 0 | F | 2 | 1 |
| 8 | 9 | 9 | 0 | P | 1 | 1 |
| 9 | 10 | 10 | 0 | F | 1 | 1 |
| 9 | 10 | 10 | 0 | 1 | 0 | 0 |
| 9 | 10 | 10 | 0 | P | 1 | 1 |
| 7 | 10 | 10 | 0 | F | 2 | 1 |
| 8 | 9 | 10 | 0 | 1 | 0 | 0 |
| 9 | 10 | 10 | 0 | 1 | 0 | 0 |
| 2 | 7 | 10 | 0 | 1 | 0 | 0 |
| 10 | 10 | 10 | 0 | F | 1 | 1 |
| 8 | 9 | 10 | 0 | P | 2 | 1 |
| 9 | 10 | 10 | 0 | F | 0 | 0 |
| 8 | 9 | 10 | 0 | F | 1 | 1 |
| 9 | 10 | 10 | 0 | P | 1 | 1 |
| 8 | 9 | 10 | 0 | 0 | 0 | 0 |
| 6 | 10 | 10 | 0 | 0 | 1 | 1 |
| 9 | 10 | 10 | 0 | F | 2 | 1 |
| 9 | 10 | 10 | 0 | F | 1 | 1 |
| 7 | 8 | 10 | 0 | 0 | 1 | 1 |
| 8 | 10 | 10 | 0 | P | 1 | 1 |
| 9 | 10 | 10 | 0 | F | 1 | 1 |
| 9 | 10 | 10 | 0 | F | 4 | 3 |
| 8 | 9 | 10 | 0 | F | 2 | 1 |
| 7 | 9 | 10 | 0 | F | 1 | 1 |
| 9 | 10 | 10 | 0 | F | 1 | 1 |
| 8 | 9 | 10 | 0 | P | 1 | 1 |
| 8 | 10 | 10 | 0 | P | 2 | 1 |
| 9 | 10 | 10 | 0 | P | 4 | 3 |
| 7 | 9 | 10 | 0 | 0 | 0 | 0 |
| 7 | 8 | 8 | 0 | 0 | 4 | 3 |
| 1 | 4 | 7 | 1 | 0 | 0 | 0 |
| 4 | 8 | 10 | 0 | F | 1 | 1 |
| 10 | 10 | 10 | 0 | F | 1 | 1 |
| 9 | 10 | 10 | 0 | P | 4 | 3 |
| 9 | 10 | 10 | 0 | P | 1 | 1 |
| 9 | 10 | 10 | 0 | P | 4 | 3 |
| 9 | 10 | 10 | 0 | 0 | 0 | 0 |
| 3 | 7 | 8 | 0 | 0 | 1 | 1 |
| 9 | 10 | 10 | 0 | F | 1 | 1 |
| 9 | 10 | 10 | 0 | 0 | 1 | 1 |

| | | | | | | |
|---|---|---|---|---|---|---|
| 9 | 10 | 10 | 0 | 0 | 1 | 1 |
| 9 | 10 | 10 | 0 | F | 1 | 1 |
| 8 | 10 | 10 | 0 | P | 1 | 1 |
| 8 | 8 | 10 | 0 | P | 2 | 1 |
| 4 | 6 | 9 | 1 | P | 1 | 1 |
| 9 | 10 | 10 | 0 | F | 4 | 3 |
| 9 | 9 | 10 | 0 | 1 | 3 | 2 |
| 3 | 10 | 10 | 0 | F | 1 | 1 |
| 7 | 9 | 10 | 0 | F | 2 | 1 |
| 9 | 10 | 10 | 0 | F | 1 | 1 |
| 9 | 10 | 10 | 0 | F | 4 | 3 |
| 9 | 10 | 10 | 0 | F | 4 | 3 |
| 9 | 10 | 10 | 0 | 0 | 0 | 0 |
| 9 | 10 | 10 | 0 | 0 | 3 | 2 |
| 9 | 10 | 10 | 0 | P | 2 | 1 |
| 7 | 10 | 10 | 0 | 0 | 3 | 2 |
| 7 | 8 | 9 | 0 | P | 1 | 1 |
| 1 | 7 | 9 | 0 | 0 | 0 | 0 |
| 5 | 7 | 9 | 0 | 1 | 0 | 0 |
| 9 | 10 | 10 | 0 | 0 | 0 | 0 |
| 9 | 10 | 10 | 0 | P | 1 | 1 |
| 9 | 10 | 10 | 0 | P | 1 | 1 |
| 9 | 9 | 10 | 0 | F | 1 | 1 |
| 9 | 10 | 10 | 0 | 0 | 0 | 0 |
| 9 | 10 | 10 | 0 | P | 1 | 1 |
| 9 | 10 | 10 | 0 | F | 2 | 1 |
| 9 | 10 | 10 | 0 | 0 | 1 | 1 |
| 9 | 10 | 10 | 0 | 0 | 0 | 0 |
| 10 | 10 | 10 | 0 | P | 1 | 1 |
| 9 | 10 | 10 | 0 | F | 1 | 1 |
| 7 | 9 | 10 | 0 | 1 | 1 | 1 |
| 9 | 10 | 10 | 0 | 1 | 0 | 0 |
| 9 | 10 | 10 | 0 | P | 1 | 1 |
| 9 | 10 | 10 | 0 | P | 1 | 1 |
| 9 | 9 | 10 | 0 | F | 1 | 1 |
| 6 | 7 | 8 | 0 | P | 2 | 1 |
| 9 | 10 | 10 | 0 | 1 | 3 | 3 |
| 6 | 8 | 9 | 0 | P | 2 | 1 |
| 7 | 9 | 10 | 0 | 0 | 3 | 2 |
| 9 | 10 | 10 | 0 | P | 4 | 3 |
| 9 | 10 | 10 | 0 | F | 1 | 1 |
| 6 | 7 | 9 | 0 | F | 0 | 0 |
| 9 | 10 | 9 | 0 | P | 2 | 1 |
| 2 | 4 | 8 | 1 | 1 | 1 | 1 |
| 9 | 10 | 10 | 0 | P | 2 | 1 |
| 9 | 10 | 10 | 0 | P | 1 | 1 |
| 5 | 7 | 8 | 0 | F | 4 | 3 |
| 8 | 9 | 10 | 0 | P | 2 | 1 |
| 9 | 10 | 10 | 0 | F | 1 | 1 |
| 1 | 6 | 8 | 1 | 1 | 1 | 1 |
| 6 | 7 | 9 | 0 | F | 2 | 1 |
| 9 | 10 | 10 | 0 | F | 1 | 1 |
| 10 | 10 | 10 | 0 | F | 1 | 1 |
| 9 | 10 | 10 | 0 | P | 1 | 1 |
| 8 | 10 | 10 | 0 | F | 1 | 1 |
| 9 | 10 | 10 | 0 | P | 1 | 1 |
| 8 | 10 | 10 | 0 | 1 | 1 | 1 |
| 9 | 10 | 10 | 0 | F | 1 | 1 |
| 6 | 7 | 8 | 0 | 1 | 1 | 1 |

| | | | | | | |
|---|---|---|---|---|---|---|
| 7 | 9 | 9 | 0 | 0 | 3 | 2 |
| 6 | 9 | 10 | 0 | P | 2 | 1 |
| 9 | 10 | 10 | 0 | 1 | 3 | 2 |
| 9 | 10 | 10 | 0 | F | 4 | 3 |
| 9 | 10 | 10 | 0 | F | 1 | 1 |
| 9 | 10 | 10 | 0 | P | 1 | 1 |
| 9 | 10 | 10 | 0 | F | 4 | 3 |
| 9 | 9 | 10 | 0 | P | 1 | 1 |
| 9 | 10 | 10 | 0 | 0 | 3 | 2 |
| 9 | 10 | 10 | 0 | P | 1 | 1 |
| 9 | 10 | 10 | 0 | 0 | 1 | 1 |
| 7 | 9 | 9 | 0 | F | 2 | 1 |
| 9 | 10 | 10 | 0 | 0 | 4 | 3 |
| 7 | 8 | 9 | 0 | P | 0 | 0 |
| 7 | 8 | 9 | 0 | F | 1 | 1 |
| 5 | 9 | 10 | 0 | P | 2 | 1 |
| 9 | 9 | 10 | 0 | F | 1 | 1 |
| 9 | 10 | 10 | 0 | F | 1 | 1 |
| 8 | 9 | 10 | 0 | P | 1 | 1 |
| 9 | 10 | 10 | 0 | P | 2 | 1 |
| 10 | 10 | 10 | 0 | 0 | 1 | 1 |
| 9 | 10 | 10 | 0 | P | 1 | 1 |
| 9 | 10 | 10 | 0 | P | 1 | 1 |
| 8 | 9 | 9 | 0 | F | 4 | 3 |
| 8 | 9 | 10 | 0 | 1 | 0 | 0 |
| 9 | 10 | 10 | 0 | F | 1 | 1 |
| 9 | 10 | 10 | 0 | F | 2 | 1 |
| 9 | 10 | 10 | 0 | F | 1 | 1 |
| 8 | 9 | 10 | 0 | 1 | 0 | 0 |
| 9 | 10 | 10 | 0 | P | 1 | 1 |
| 9 | 9 | 10 | 0 | P | 1 | 1 |
| 9 | 10 | 10 | 0 | P | 1 | 1 |
| 9 | 10 | 10 | 0 | F | 1 | 1 |
| 9 | 10 | 10 | 0 | 1 | 1 | 1 |
| 4 | 9 | 10 | 0 | P | 2 | 1 |
| 9 | 10 | 10 | 0 | P | 4 | 3 |
| 9 | 10 | 10 | 0 | 1 | 0 | 0 |
| 8 | 9 | 10 | 0 | F | 1 | 1 |
| 8 | 9 | 10 | 0 | 0 | 0 | 0 |
| 9 | 10 | 10 | 0 | F | 1 | 1 |
| 9 | 10 | 10 | 0 | 1 | 1 | 1 |
| 9 | 10 | 10 | 0 | P | 1 | 1 |
| 8 | 9 | 9 | 0 | 0 | 3 | 2 |
| 8 | 9 | 10 | 0 | P | 2 | 1 |
| 8 | 10 | 10 | 0 | P | 2 | 1 |
| 8 | 10 | 10 | 0 | 0 | 0 | 0 |
| 9 | 10 | 10 | 0 | P | 1 | 1 |
| 9 | 10 | 10 | 0 | P | 1 | 1 |
| 9 | 10 | 10 | 0 | F | 1 | 1 |
| 5 | 10 | 10 | 0 | 0 | 3 | 2 |
| 9 | 10 | 10 | 0 | P | 1 | 1 |
| 3 | 8 | 10 | 0 | 1 | 1 | 1 |
| 10 | 10 | 10 | 0 | P | 1 | 1 |
| 8 | 10 | 10 | 0 | P | 1 | 1 |
| 6 | 9 | 10 | 0 | 1 | 0 | 0 |
| 9 | 10 | 10 | 0 | 1 | 3 | 2 |
| 9 | 10 | 10 | 0 | F | 1 | 1 |
| 9 | 10 | 10 | 0 | F | 1 | 1 |
| 9 | 10 | 10 | 0 | 0 | 3 | 2 |

| | | | | | | | |
|---|---|---|---|---|---|---|---|
| 9 | 10 | 10 | 0 | P | | 1 | 1 |
| 9 | 10 | 10 | 0 | F | | 1 | 1 |
| 6 | 8 | 9 | 0 | F | | 1 | 1 |
| 9 | 10 | 10 | 0 | | 1 | 0 | 0 |
| 9 | 10 | 10 | 0 | P | | 2 | 1 |
| 9 | 10 | 10 | 0 | | 1 | 0 | 0 |
| 5 | 9 | 9 | 0 | P | | 1 | 1 |
| 8 | 10 | 10 | 0 | P | | 1 | 1 |
| 9 | 10 | 10 | 0 | | 1 | 0 | 0 |
| 4 | 9 | 9 | 0 | F | | 4 | 3 |
| 9 | 10 | 10 | 0 | F | | 1 | 1 |
| 9 | 10 | 10 | 0 | F | | 2 | 1 |
| 2 | 10 | 10 | 0 | | 0 | 3 | 2 |
| 7 | 9 | 10 | 0 | P | | 1 | 1 |
| 8 | 8 | 10 | 0 | | 1 | 3 | 2 |
| 8 | 9 | 10 | 0 | P | | 1 | 1 |
| 9 | 10 | 10 | 0 | P | | 1 | 1 |
| 7 | 8 | 9 | 0 | | 1 | 0 | 0 |
| 9 | 10 | 10 | 0 | | 1 | 3 | 2 |
| 6 | 10 | 10 | 0 | | 1 | 1 | 1 |
| 9 | 10 | 10 | 0 | | 0 | 0 | 0 |
| 9 | 10 | 10 | 0 | P | | 1 | 1 |
| 9 | 10 | 10 | 0 | F | | 1 | 1 |
| 9 | 10 | 10 | 0 | | 0 | 1 | 1 |
| 9 | 10 | 10 | 0 | | 0 | 0 | 0 |
| 5 | 8 | 9 | 0 | | 1 | 0 | 0 |
| 8 | 10 | 10 | 0 | P | | 1 | 1 |
| 5 | 7 | 10 | 0 | P | | 1 | 1 |
| 9 | 10 | 10 | 0 | P | | 4 | 3 |
| 9 | 10 | 10 | 0 | P | | 1 | 1 |
| 8 | 9 | 10 | 0 | P | | 2 | 1 |
| 10 | 10 | 10 | 0 | F | | 1 | 1 |
| 9 | 10 | 10 | 0 | P | | 2 | 1 |
| 9 | 10 | 10 | 0 | F | | 2 | 1 |
| 10 | 10 | 10 | 0 | F | | 1 | 1 |
| 8 | 9 | 10 | 0 | P | | 1 | 1 |
| 9 | 10 | 10 | 0 | P | | 1 | 1 |
| 9 | 10 | 10 | 0 | P | | 1 | 1 |
| 6 | 8 | 8 | 0 | P | | 2 | 1 |
| 9 | 10 | 10 | 0 | F | | 1 | 1 |
| 8 | 8 | 9 | 0 | F | | 2 | 1 |
| 9 | 10 | 10 | 0 | P | | 2 | 1 |
| 5 | 7 | 9 | 0 | F | | 1 | 1 |
| 9 | 10 | 10 | 0 | F | | 1 | 1 |
| 8 | 10 | 10 | 0 | P | | 2 | 1 |
| 3 | 6 | 6 | 1 | | 1 | 0 | 0 |
| 9 | 10 | 10 | 0 | | 0 | 2 | 1 |
| 10 | 10 | 10 | 0 | | 1 | 3 | 2 |
| 3 | 9 | 9 | 0 | | 0 | 0 | 0 |
| 9 | 10 | 10 | 0 | | 0 | 0 | 0 |
| 7 | 10 | 10 | 0 | F | | 1 | 1 |
| 9 | 10 | 10 | 0 | | 1 | 0 | 0 |
| 5 | 8 | 10 | 0 | | 1 | 1 | 1 |
| 9 | 10 | 10 | 0 | | 1 | 1 | 1 |
| 9 | 10 | 10 | 0 | | 1 | 3 | 2 |
| 9 | 10 | 10 | 0 | F | | 1 | 1 |
| 9 | 10 | 10 | 0 | F | | 2 | 1 |
| 9 | 10 | 10 | 0 | P | | 1 | 1 |
| 2 | 7 | 10 | 0 | | 0 | 4 | 3 |

| | | | | | | | |
|---|---|---|---|---|---|---|---|
| 9 | 10 | 10 | 0 | F | | 1 | 1 |
| 9 | 10 | 10 | 0 | P | | 1 | 1 |
| 8 | 9 | 10 | 0 | P | | 1 | 1 |
| 7 | 9 | 10 | 0 | | 1 | 4 | 3 |
| 8 | 9 | 10 | 0 | F | | 1 | 1 |
| 6 | 7 | 8 | 0 | | 0 | 3 | 2 |
| 9 | 10 | 10 | 0 | F | | 1 | 1 |
| 6 | 9 | 10 | 0 | | 0 | 1 | 1 |
| 6 | 9 | 10 | 0 | F | | 1 | 1 |
| 9 | 10 | 10 | 0 | P | | 2 | 1 |
| 9 | 9 | 9 | 0 | F | | 1 | 1 |
| 9 | 10 | 10 | 0 | | 0 | 3 | 2 |
| 9 | 10 | 10 | 0 | | 0 | 0 | 0 |
| 4 | 7 | 10 | 0 | P | | 2 | 1 |
| 8 | 9 | 10 | 0 | | 1 | 0 | 0 |
| 6 | 9 | 10 | 0 | P | | 2 | 1 |
| 6 | 10 | 10 | 0 | | 1 | 0 | 0 |
| 8 | 9 | 10 | 0 | | 0 | 0 | 0 |
| 9 | 10 | 10 | 0 | F | | 4 | 3 |
| 9 | 10 | 10 | 0 | | 0 | 3 | 2 |
| 9 | 10 | 10 | 0 | F | | 2 | 1 |
| 8 | 10 | 10 | 0 | | 0 | 0 | 0 |
| 9 | 10 | 10 | 0 | | 1 | 0 | 0 |
| 9 | 10 | 10 | 0 | F | | 1 | 1 |
| 8 | 8 | 9 | 0 | | 0 | 0 | 0 |
| 2 | 8 | 8 | 0 | | 0 | 0 | 0 |
| 9 | 10 | 10 | 0 | P | | 1 | 1 |
| 0 | 2 | 3 | 1 | | 1 | 4 | 3 |
| 2 | 6 | 10 | 1 | | 0 | 0 | 0 |
| 9 | 10 | 10 | 0 | F | | 4 | 3 |
| 9 | 10 | 10 | 0 | F | | 4 | 3 |
| 9 | 10 | 10 | 0 | | 0 | 1 | 1 |
| 9 | 10 | 10 | 0 | P | | 2 | 1 |
| 9 | 10 | 10 | 0 | | 0 | 4 | 3 |
| 9 | 10 | 10 | 0 | | 1 | 1 | 1 |
| 8 | 10 | 10 | 0 | P | | 1 | 1 |
| 6 | 9 | 10 | 0 | | 1 | 3 | 2 |
| 9 | 10 | 10 | 0 | | 0 | 3 | 2 |
| 9 | 10 | 10 | 0 | P | | 4 | 3 |
| 8 | 9 | 10 | 0 | | 0 | 0 | 0 |
| 9 | 10 | 10 | 0 | F | | 4 | 3 |
| 8 | 9 | 10 | 0 | | 1 | 1 | 1 |
| 9 | 10 | 10 | 0 | F | | 1 | 1 |
| 9 | 9 | 10 | 0 | | 1 | 3 | 2 |
| 9 | 9 | 10 | 0 | P | | 4 | 3 |
| 8 | 9 | 9 | 0 | | 1 | 0 | 0 |
| 6 | 9 | 10 | 0 | F | | 1 | 1 |
| 6 | 8 | 10 | 0 | | 1 | 4 | 3 |
| 4 | 6 | 8 | 1 | | 1 | 3 | 2 |
| 1 | 6 | 7 | 1 | | 0 | 0 | 0 |
| 9 | 10 | 10 | 0 | F | | 1 | 1 |
| 9 | 10 | 10 | 0 | P | | 1 | 1 |
| 5 | 10 | 10 | 0 | F | | 1 | 1 |
| 9 | 10 | 10 | 0 | | 1 | 1 | 1 |
| 9 | 10 | 10 | 0 | P | | 1 | 1 |
| 9 | 10 | 10 | 0 | P | | 1 | 1 |
| 9 | 10 | 10 | 0 | | 1 | 1 | 1 |
| 9 | 10 | 10 | 0 | F | | 1 | 1 |
| 7 | 7 | 8 | 0 | | 1 | 0 | 0 |

| | | | | | | |
|---|---|---|---|---|---|---|
| 7 | 8 | 9 | 0 | 1 | 0 | 0 |
| 8 | 10 | 10 | 0 | P | 1 | 1 |
| 9 | 10 | 10 | 0 | 1 | 0 | 0 |
| 9 | 10 | 10 | 0 | P | 1 | 1 |
| 1 | 10 | 10 | 0 | 1 | 0 | 0 |
| 8 | 10 | 10 | 0 | 1 | 3 | 2 |
| 8 | 9 | 9 | 0 | F | 2 | 1 |
| 10 | 10 | 10 | 0 | 1 | 4 | 3 |
| 1 | 3 | 6 | 1 | 1 | 3 | 2 |
| 9 | 10 | 10 | 0 | 0 | 1 | 1 |
| 8 | 9 | 10 | 0 | F | 4 | 3 |
| 9 | 10 | 10 | 0 | F | 1 | 1 |
| 9 | 10 | 10 | 0 | 0 | 0 | 0 |
| 1 | 9 | 9 | 0 | 0 | 0 | 0 |
| 9 | 10 | 10 | 0 | P | 2 | 1 |
| 6 | 7 | 8 | 0 | F | 1 | 1 |
| 6 | 8 | 8 | 0 | 0 | 0 | 0 |
| 9 | 10 | 10 | 0 | F | 1 | 1 |
| 2 | 3 | 7 | 1 | F | 1 | 1 |
| 9 | 9 | 10 | 0 | 1 | 3 | 2 |
| 6 | 9 | 9 | 0 | 1 | 4 | 3 |
| 9 | 10 | 10 | 0 | 1 | 0 | 0 |
| 9 | 10 | 10 | 0 | F | 1 | 1 |
| 9 | 10 | 10 | 0 | F | 4 | 3 |
| 9 | 10 | 10 | 0 | 0 | 3 | 2 |
| 7 | 8 | 9 | 0 | P | 1 | 1 |
| 6 | 10 | 10 | 0 | 1 | 0 | 0 |
| 8 | 9 | 10 | 0 | P | 1 | 1 |
| 9 | 10 | 10 | 0 | P | 1 | 1 |
| 9 | 10 | 10 | 0 | 1 | 0 | 0 |
| 8 | 10 | 10 | 0 | 1 | 1 | 1 |
| 9 | 10 | 10 | 0 | F | 1 | 1 |
| 9 | 10 | 10 | 0 | 0 | 0 | 0 |
| 9 | 10 | 10 | 0 | 0 | 0 | 0 |
| 3 | 7 | 10 | 0 | 1 | 3 | 2 |
| 5 | 10 | 10 | 0 | P | 2 | 1 |
| 6 | 8 | 8 | 0 | P | 2 | 1 |
| 7 | 10 | 10 | 0 | F | 4 | 3 |
| 9 | 10 | 10 | 0 | P | 1 | 1 |
| 4 | 8 | 10 | 0 | 0 | 2 | 1 |
| 5 | 7 | 9 | 0 | 0 | 0 | 0 |
| 5 | 7 | 8 | 0 | 1 | 3 | 2 |
| 5 | 9 | 9 | 0 | 1 | 3 | 2 |
| 8 | 10 | 10 | 0 | 0 | 0 | 0 |
| 1 | 7 | 8 | 0 | F | 2 | 1 |
| 6 | 8 | 10 | 0 | P | 2 | 1 |
| 9 | 10 | 10 | 0 | F | 1 | 1 |
| 9 | 10 | 10 | 0 | F | 1 | 1 |
| 8 | 10 | 10 | 0 | 0 | 0 | 0 |
| 4 | 7 | 10 | 0 | 0 | 1 | 1 |
| 4 | 7 | 9 | 0 | F | 1 | 1 |
| 1 | 6 | 8 | 1 | 1 | 0 | 0 |
| 6 | 8 | 10 | 0 | 1 | 0 | 0 |
| 7 | 8 | 8 | 0 | P | 4 | 3 |
| 9 | 10 | 10 | 0 | F | 1 | 1 |
| 9 | 10 | 10 | 0 | P | 1 | 1 |
| 9 | 10 | 10 | 0 | 0 | 0 | 0 |
| 9 | 10 | 10 | 0 | F | 1 | 1 |
| 9 | 9 | 9 | 0 | F | 1 | 1 |

| | | | | | | |
|---|---|---|---|---|---|---|
| 9 | 10 | 10 | 0 | 1 | 0 | 0 |
| 9 | 10 | 10 | 0 | F | 2 | 1 |
| 9 | 10 | 10 | 0 | F | 1 | 1 |
| 10 | 10 | 10 | 0 | P | 1 | 1 |
| 8 | 9 | 10 | 0 | 0 | 0 | 0 |
| 9 | 10 | 10 | 0 | F | 1 | 1 |
| 8 | 9 | 10 | 0 | 1 | 0 | 0 |
| 6 | 9 | 10 | 0 | P | 2 | 1 |
| 9 | 10 | 10 | 0 | F | 1 | 1 |
| 9 | 10 | 10 | 0 | F | 2 | 1 |
| 6 | 6 | 8 | 1 | P | 4 | 3 |
| 7 | 8 | 9 | 0 | 1 | 0 | 0 |
| 8 | 8 | 10 | 0 | 0 | 2 | 1 |
| 5 | 9 | 10 | 0 | 1 | 0 | 0 |
| 2 | 6 | 8 | 1 | P | 1 | 1 |
| 9 | 10 | 10 | 0 | 0 | 1 | 1 |
| 9 | 10 | 10 | 0 | P | 4 | 3 |
| 9 | 10 | 10 | 0 | 0 | 1 | 1 |
| 8 | 9 | 10 | 0 | 1 | 1 | 1 |
| 9 | 10 | 10 | 0 | F | 2 | 1 |
| 3 | 8 | 10 | 0 | F | 2 | 1 |
| 7 | 9 | 10 | 0 | 1 | 1 | 1 |
| 9 | 10 | 10 | 0 | 1 | 0 | 0 |
| 4 | 7 | 9 | 0 | 1 | 0 | 0 |
| 6 | 10 | 10 | 0 | 1 | 0 | 0 |
| 7 | 10 | 10 | 0 | 1 | 3 | 2 |
| 8 | 9 | 10 | 0 | 1 | 3 | 2 |
| 9 | 10 | 10 | 0 | 0 | 0 | 0 |
| 8 | 8 | 8 | 0 | 0 | 0 | 0 |
| 9 | 10 | 10 | 0 | P | 1 | 1 |
| 6 | 4 | 8 | 1 | 0 | 3 | 2 |
| 6 | 9 | 10 | 0 | 1 | 1 | 1 |
| 7 | 9 | 10 | 0 | F | 2 | 1 |
| 9 | 10 | 10 | 0 | F | 4 | 3 |
| 6 | 9 | 10 | 0 | 0 | 1 | 1 |
| 9 | 10 | 10 | 0 | 1 | 0 | 0 |
| 5 | 8 | 9 | 0 | 0 | 0 | 0 |
| 6 | 8 | 9 | 0 | P | 2 | 1 |
| 7 | 10 | 10 | 0 | F | 4 | 3 |
| 7 | 8 | 8 | 0 | 0 | 3 | 2 |
| 7 | 10 | 10 | 0 | F | 2 | 1 |
| 9 | 9 | 10 | 0 | 0 | 0 | 0 |
| 9 | 10 | 10 | 0 | 1 | 0 | 0 |
| 6 | 6 | 8 | 1 | 1 | 0 | 0 |
| 9 | 10 | 10 | 0 | F | 0 | 0 |
| 9 | 10 | 10 | 0 | P | 2 | 1 |
| 9 | 10 | 10 | 0 | 1 | 1 | 1 |
| 8 | 8 | 9 | 0 | 1 | 0 | 0 |
| 9 | 10 | 10 | 0 | 0 | 3 | 2 |
| 7 | 9 | 9 | 0 | P | 1 | 1 |
| 6 | 8 | 9 | 0 | 0 | 3 | 2 |
| 7 | 9 | 10 | 0 | 0 | 3 | 2 |
| 9 | 10 | 10 | 0 | F | 1 | 1 |
| 9 | 10 | 10 | 0 | 1 | 0 | 0 |
| 9 | 10 | 10 | 0 | P | 1 | 1 |
| 9 | 9 | 10 | 0 | P | 1 | 1 |
| 9 | 9 | 10 | 0 | P | 1 | 1 |
| 9 | 10 | 10 | 0 | 0 | 1 | 1 |
| 9 | 10 | 10 | 0 | 0 | 0 | 0 |

| | | | | | | | |
|---|---|---|---|---|---|---|---|
| 9 | 10 | 10 | 0 | F | | 1 | 1 |
| 9 | 10 | 10 | 0 | 0 | | 1 | 1 |
| 7 | 10 | 10 | 0 | 1 | | 0 | 0 |
| 9 | 10 | 10 | 0 | 1 | | 0 | 0 |
| 9 | 10 | 10 | 0 | 1 | | 0 | 0 |
| 9 | 10 | 10 | 0 | P | | 4 | 3 |
| 7 | 10 | 10 | 0 | 1 | | 1 | 1 |
| 9 | 10 | 10 | 0 | P | | 1 | 1 |
| 2 | 7 | 8 | 0 | 1 | | 0 | 0 |
| 8 | 10 | 10 | 0 | P | | 2 | 1 |
| 8 | 9 | 10 | 0 | 0 | | 0 | 0 |
| 9 | 10 | 10 | 0 | 0 | | 0 | 0 |
| 9 | 10 | 10 | 0 | 0 | | 0 | 0 |
| 9 | 10 | 10 | 0 | 0 | | 0 | 0 |
| 8 | 10 | 10 | 0 | P | | 1 | 1 |
| 8 | 10 | 10 | 0 | 1 | | 1 | 1 |
| 8 | 9 | 10 | 0 | 0 | | 0 | 0 |
| 2 | 8 | 9 | 0 | 1 | | 3 | 2 |
| 8 | 10 | 10 | 0 | 1 | | 3 | 2 |
| 6 | 9 | 10 | 0 | 0 | | 3 | 2 |
| 3 | 8 | 8 | 0 | 1 | | 1 | 1 |
| 3 | 9 | 10 | 0 | 0 | | 1 | 1 |
| 9 | 10 | 10 | 0 | 0 | | 0 | 0 |
| 6 | 9 | 9 | 0 | 1 | | 1 | 1 |
| 9 | 10 | 10 | 0 | 0 | | 1 | 1 |
| 9 | 10 | 10 | 0 | P | | 0 | 0 |
| 5 | 8 | 9 | 0 | 0 | | 0 | 0 |
| 7 | 8 | 9 | 0 | P | | 2 | 1 |
| 9 | 9 | 10 | 0 | F | | 1 | 1 |
| 9 | 10 | 10 | 0 | F | | 1 | 1 |
| 9 | 10 | 10 | 0 | P | | 1 | 1 |
| 8 | 9 | 10 | 0 | 1 | | 0 | 0 |
| 9 | 9 | 10 | 0 | P | | 1 | 1 |
| 5 | 8 | 9 | 0 | 0 | | 0 | 0 |
| 9 | 10 | 10 | 0 | 1 | | 0 | 0 |
| 7 | 8 | 10 | 0 | 0 | | 0 | 0 |
| 7 | 10 | 10 | 0 | P | | 2 | 1 |
| 8 | 10 | 10 | 0 | 0 | | 1 | 1 |
| 8 | 9 | 10 | 0 | 0 | | 0 | 0 |
| 9 | 10 | 10 | 0 | P | | 1 | 1 |
| 9 | 10 | 10 | 0 | F | | 1 | 1 |
| 1 | 6 | 8 | 1 | 0 | | 0 | 0 |
| 6 | 8 | 9 | 0 | 0 | | 0 | 0 |
| 9 | 10 | 10 | 0 | 0 | | 1 | 1 |
| 8 | 7 | 7 | 0 | F | | 1 | 1 |
| 8 | 9 | 10 | 0 | 1 | | 0 | 0 |
| 7 | 10 | 10 | 0 | 0 | | 0 | 0 |
| 9 | 10 | 10 | 0 | F | | 2 | 1 |
| 8 | 10 | 10 | 0 | P | | 1 | 1 |
| 1 | 6 | 9 | 1 | 1 | | 1 | 1 |
| 9 | 10 | 10 | 0 | 1 | | 0 | 0 |
| 9 | 10 | 10 | 0 | F | | 1 | 1 |
| 9 | 10 | 10 | 0 | F | | 1 | 1 |
| 9 | 10 | 10 | 0 | F | | 1 | 1 |
| 8 | 9 | 10 | 0 | 0 | | 1 | 1 |
| 3 | 8 | 10 | 0 | 0 | | 0 | 0 |
| 9 | 10 | 10 | 0 | P | | 4 | 3 |
| 9 | 10 | 10 | 0 | F | | 4 | 3 |
| 9 | 10 | 10 | 0 | F | | 1 | 1 |

| | | | | | | | |
|---|---|---|---|---|---|---|---|
| 3 | 6 | 8 | 1 | 0 | | 1 | 1 |
| 9 | 10 | 10 | 0 | 0 | | 3 | 2 |
| 4 | 10 | 10 | 0 | 0 | | 3 | 2 |
| 9 | 9 | 10 | 0 | 0 | | 1 | 1 |
| 8 | 9 | 9 | 0 | 0 | | 3 | 2 |
| 9 | 10 | 10 | 0 | F | | 1 | 1 |
| 5 | 10 | 10 | 0 | 0 | | 0 | 0 |
| 1 | 7 | 9 | 0 | 1 | | 0 | 0 |
| 9 | 10 | 10 | 0 | F | | 1 | 1 |
| 9 | 10 | 10 | 0 | 0 | | 3 | 2 |
| 8 | 10 | 10 | 0 | 1 | | 3 | 2 |
| 4 | 8 | 9 | 0 | 0 | | 3 | 2 |
| 4 | 8 | 9 | 0 | 1 | | 3 | 2 |
| 9 | 10 | 10 | 0 | 1 | | 0 | 0 |
| 3 | 7 | 10 | 0 | 1 | | 1 | 1 |
| 5 | 8 | 9 | 0 | F | | 2 | 1 |
| 9 | 10 | 10 | 0 | 1 | | 0 | 0 |
| 9 | 10 | 10 | 0 | 1 | | 0 | 0 |
| 7 | 9 | 9 | 0 | 0 | | 1 | 1 |
| 9 | 10 | 10 | 0 | 0 | | 1 | 1 |
| 8 | 10 | 10 | 0 | 0 | | 1 | 1 |
| 8 | 10 | 10 | 0 | P | | 2 | 1 |
| 9 | 10 | 10 | 0 | F | | 1 | 1 |
| 9 | 9 | 10 | 0 | 0 | | 3 | 2 |
| 4 | 9 | 10 | 0 | 0 | | 3 | 2 |
| 8 | 8 | 10 | 0 | 0 | | 3 | 2 |
| 6 | 8 | 9 | 0 | 0 | | 0 | 0 |
| 9 | 10 | 10 | 0 | F | | 4 | 3 |
| 9 | 9 | 9 | 0 | 1 | | 1 | 1 |
| 9 | 10 | 10 | 0 | P | | 4 | 3 |
| 8 | 10 | 10 | 0 | 0 | | 1 | 1 |
| 9 | 10 | 10 | 0 | 0 | | 1 | 1 |
| 9 | 10 | 10 | 0 | P | | 1 | 1 |
| 8 | 9 | 10 | 0 | P | | 2 | 1 |
| 9 | 10 | 10 | 0 | 1 | | 3 | 2 |
| 3 | 6 | 8 | 1 | 1 | | 1 | 1 |
| 9 | 10 | 10 | 0 | P | | 2 | 1 |
| 9 | 10 | 10 | 0 | 0 | | 0 | 0 |
| 7 | 8 | 10 | 0 | F | | 2 | 1 |
| 4 | 8 | 10 | 0 | F | | 1 | 1 |
| 7 | 9 | 10 | 0 | 1 | | 0 | 0 |
| 8 | 9 | 10 | 0 | 1 | | 0 | 0 |
| 9 | 10 | 10 | 0 | P | | 2 | 1 |
| 9 | 9 | 10 | 0 | P | | 1 | 1 |
| 9 | 9 | 10 | 0 | P | | 1 | 1 |
| 8 | 8 | 9 | 0 | F | | 1 | 1 |
| 8 | 10 | 10 | 0 | 1 | | 3 | 2 |
| 9 | 9 | 10 | 0 | 1 | | 3 | 2 |
| 2 | 3 | 8 | 1 | 0 | | 3 | 2 |
| 9 | 9 | 10 | 0 | P | | 4 | 3 |
| 9 | 10 | 10 | 0 | 0 | | 0 | 0 |
| 2 | 6 | 10 | 1 | 1 | | 3 | 2 |
| 8 | 10 | 10 | 0 | 1 | | 4 | 3 |
| 9 | 10 | 10 | 0 | P | | 1 | 1 |
| 9 | 10 | 10 | 0 | 1 | | 4 | 3 |
| 7 | 8 | 9 | 0 | P | | 4 | 3 |
| 1 | 5 | 7 | 1 | P | | 1 | 1 |
| 9 | 10 | 10 | 0 | P | | 1 | 1 |
| 5 | 9 | 10 | 0 | 0 | | 0 | 0 |

| | | | | | | |
|---|---|---|---|---|---|---|
| 6 | 9 | 7 | 0 | P | 2 | 1 |
| 9 | 10 | 10 | 0 | 1 | 1 | 1 |
| 9 | 10 | 10 | 0 | F | 1 | 1 |
| 7 | 8 | 9 | 0 | F | 2 | 1 |
| 9 | 10 | 10 | 0 | P | 1 | 1 |
| 8 | 10 | 10 | 0 | P | 1 | 1 |
| 7 | 9 | 10 | 0 | P | 2 | 1 |
| 9 | 10 | 10 | 0 | 1 | 1 | 1 |
| 9 | 10 | 10 | 0 | 0 | 3 | 2 |
| 9 | 10 | 10 | 0 | F | 4 | 3 |
| 6 | 10 | 10 | 0 | F | 1 | 1 |
| 9 | 10 | 10 | 0 | F | 1 | 1 |
| 9 | 10 | 10 | 0 | P | 4 | 3 |
| 9 | 10 | 10 | 0 | F | 1 | 1 |
| 9 | 10 | 10 | 0 | F | 1 | 1 |
| 6 | 7 | 8 | 0 | 0 | 0 | 0 |
| 9 | 10 | 10 | 0 | 1 | 0 | 0 |
| 1 | 7 | 8 | 0 | P | 1 | 1 |
| 9 | 10 | 10 | 0 | F | 1 | 1 |
| 9 | 10 | 10 | 0 | 0 | 4 | 3 |
| 4 | 8 | 8 | 0 | 0 | 0 | 0 |
| 8 | 9 | 10 | 0 | 0 | 1 | 1 |
| 9 | 10 | 10 | 0 | P | 2 | 1 |
| 7 | 8 | 9 | 0 | 0 | 3 | 2 |
| 9 | 10 | 10 | 0 | P | 1 | 1 |
| 8 | 9 | 10 | 0 | P | 2 | 1 |
| 2 | 6 | 7 | 1 | 1 | 1 | 1 |
| 8 | 9 | 10 | 0 | F | 1 | 1 |
| 9 | 10 | 10 | 0 | F | 1 | 1 |
| 9 | 10 | 10 | 0 | 0 | 1 | 1 |
| 9 | 10 | 10 | 0 | P | 1 | 1 |
| 9 | 10 | 10 | 0 | F | 1 | 1 |
| 9 | 10 | 10 | 0 | 1 | 0 | 0 |
| 9 | 10 | 10 | 0 | F | 1 | 1 |
| 7 | 10 | 10 | 0 | 0 | 3 | 2 |
| 10 | 10 | 10 | 0 | P | 1 | 1 |
| 9 | 10 | 10 | 0 | 1 | 0 | 0 |
| 9 | 10 | 10 | 0 | 1 | 0 | 0 |
| 1 | 1 | 0 | 1 | 1 | 3 | 2 |
| 8 | 9 | 9 | 0 | F | 0 | 0 |
| 8 | 10 | 10 | 0 | F | 2 | 1 |
| 4 | 8 | 10 | 0 | 1 | 3 | 2 |
| 5 | 9 | 10 | 0 | P | 2 | 1 |
| 9 | 10 | 10 | 0 | F | 1 | 1 |
| 9 | 10 | 10 | 0 | F | 1 | 1 |
| 9 | 10 | 10 | 0 | 1 | 0 | 0 |
| 10 | 10 | 10 | 0 | F | 1 | 1 |
| 9 | 10 | 10 | 0 | F | 1 | 1 |
| 9 | 10 | 10 | 0 | 1 | 3 | 2 |
| 6 | 10 | 10 | 0 | 1 | 3 | 2 |
| 1 | 6 | 8 | 1 | F | 2 | 1 |
| 8 | 9 | 10 | 0 | P | 1 | 1 |
| 6 | 10 | 10 | 0 | 1 | 0 | 0 |
| | | | | | | |
| 9 | 10 | 10 | 0 | 0 | 0 | 0 |
| | | | | | | |
| 9 | 10 | 10 | 0 | 1 | 3 | 2 |
| 4 | 9 | 10 | 0 | 0 | 0 | 0 |
| | | | | | | |

| | | | | | | |
|---|---|---|---|---|---|---|
| 5 | 7 | 8 | 0 | P | 0 | 0 |
| 3 | 4 | 5 | 1 | 1 | 0 | 0 |
| 8 | 9 | 10 | 0 | 1 | 0 | 0 |
| 8 | 8 | 9 | 0 | 0 | 0 | 0 |
| | | | | | | |
| 8 | 9 | 9 | 0 | P | 2 | 1 |
| 9 | 10 | 10 | 0 | 0 | 1 | 1 |
| | | | | | | |
| 8 | 9 | 9 | 0 | 1 | 3 | 2 |
| | | | | | | |
| 6 | 10 | 10 | 0 | 1 | 0 | 0 |
| 9 | 10 | 10 | 0 | 0 | 0 | 0 |
| 8 | 10 | 10 | 0 | F | 2 | 1 |
| 2 | 6 | 10 | 1 | 1 | 1 | 1 |
| | | | | | | |
| 9 | 10 | 10 | 0 | P | 1 | 1 |
| 9 | 10 | 10 | 0 | 1 | 3 | 2 |
| 8 | 10 | 10 | 0 | 0 | 1 | 1 |
| 9 | 8 | 10 | 0 | F | 1 | 1 |
| | | | | | | |
| | | | | | | |
| 9 | 10 | 10 | 0 | F | 1 | 1 |
| 4 | 7 | 9 | 0 | 1 | 1 | 1 |
| 9 | 9 | 10 | 0 | 0 | 4 | 3 |
| 8 | 9 | 10 | 0 | F | 1 | 1 |
| | | | | | | |
| | | | | | | |
| | | | | | | |
| 2 | 3 | 7 | 1 | 0 | 1 | 1 |
| 9 | 10 | 10 | 0 | 1 | 1 | 1 |
| 8 | 9 | 10 | 0 | 0 | 1 | 1 |

| Upplivning | PH navelartär | BD- navelartär | Metabolic | NICU | Antal dagar | HIE 0=nej, |
|---|---|---|---|---|---|---|
| 0 | 7,26 | -4,3 | 0 | 0 | | 0 |
| 0 | 7,20 | 10,3 | 0 | 0 | | 0 |
| 0 | 7,23 | 3,9 | 0 | 0 | | 0 |
| 0 | 7,28 | -2,4 | 0 | 0 | | 0 |
| 0 | 7,29 | 6 | 0 | 0 | | 0 |
| 0 | 7,26 | -3,5 | 0 | 0 | | 0 |
| 0 | 7,28 | -3,8 | 0 | 0 | | 0 |
| 0 | 7,2 | 8 | 0 | 0 | | 0 |
| 0 | 7,06 | 10 | 0 | 0 | | 0 |
| 0 | | | | 0 | | 0 |
| 0 | 7,21 | 5 | 0 | 0 | | 0 |
| 0 | 7,29 | -4,7 | 0 | 0 | | 0 |
| 0 | 7,26 | -6,3 | 0 | 0 | | 0 |
| 0 | | | | 0 | | 0 |
| 0 | 7,24 | 7,1 | 0 | 0 | | 0 |
| 0 | 7,21 | 2,6 | 0 | 0 | | 0 |
| 0 | 6,95 | 18 | 1 | 0 | | 0 |
| 0 | 7,24 | 6,2 | 0 | 1 | 6 | 0 |
| 0 | 7,29 | -5,4 | 0 | 0 | | 0 |
| 0 | 7,28 | 1 | 0 | 0 | | 0 |
| 0 | 7,27 | 1 | 0 | 0 | | 0 |
| 0 | 7,15 | 5,7 | 0 | 0 | | 0 |
| 0 | 7,17 | 2 | 0 | 0 | | 0 |
| 0 | 7,16 | -7 | 0 | 0 | | 0 |
| 0 | 7,09 | 11 | 0 | 0 | | 0 |
| 0 | 7,27 | 4 | 0 | 0 | | 0 |
| 0 | 7,13 | 7 | 0 | 0 | | 0 |
| 0 | 7,36 | 4,7 | 0 | 0 | | 0 |
| 0 | 7,21 | 6,3 | 0 | 0 | | 0 |
| 0 | 7,28 | 4 | 0 | 0 | | 0 |
| 0 | 7,25 | 0 | 0 | 0 | | 0 |
| 0 | 7,19 | -5,3 | 0 | 0 | | 0 |
| 0 | 7,09 | 9 | 0 | 1 | 2 | 0 |
| 0 | 7,06 | -15,6 | 0 | 0 | | 0 |
| 0 | 7,23 | 7 | 0 | 0 | | 0 |
| 0 | 7,14 | 6 | 0 | 0 | | 0 |
| 0 | 7,19 | -9,3 | 0 | 0 | | 0 |
| 0 | 7,18 | -6,9 | 0 | 0 | | 0 |
| 0 | 7,26 | -2,6 | 0 | 0 | | 0 |
| 0 | 7,28 | 4,6 | 0 | 0 | | 0 |
| 0 | 7,20 | 9 | 0 | 0 | | 0 |
| 0 | | | | 0 | | 0 |
| 0 | 7,19 | -6,8 | 0 | 0 | | 0 |
| 0 | 7,14 | -9,2 | 0 | 0 | | 0 |
| 0 | 7,28 | 5 | 0 | 0 | | 0 |
| 0 | 7,30 | 0,4 | 0 | 0 | | 0 |
| 0 | 6,97 | -17,5 | 1 | 0 | | 0 |
| 1 | 7,035 | 15,7 | 0 | 0 | | 0 |
| 0 | 7,18 | 19 | 0 | 0 | | 0 |
| 0 | 7,20 | 6,7 | 0 | 0 | | 0 |
| 0 | 7,26 | 7,6 | 0 | 0 | | 0 |
| 0 | | | | 0 | | 0 |
| 0 | 7,25 | 6,9 | 0 | 0 | | 0 |
| 0 | 7,29 | 1,7 | 0 | 0 | | 0 |
| 0 | 7,31 | 6 | 0 | 0 | | 0 |
| 0 | 7,66 | 16,6 | 0 | 1 | 3 | 0 |
| 0 | | | 0 | 0 | | 0 |
| 0 | 7,27 | -8,7 | 0 | 0 | | 0 |

| | | | | | | |
|---|---|---|---|---|---|---|
| 0 | 7,31 | 2,7 | 0 | 0 | | 0 |
| 0 | 7,14 | 9,5 | 0 | 0 | | 0 |
| 0 | 7,00 | 15,2 | 1 | 0 | | 0 |
| 0 | 7,21 | 7,2 | 0 | 0 | | 0 |
| 0 | 7,01 | -13,5 | 1 | 0 | | 0 |
| 0 | 7,33 | 2,9 | 0 | 0 | | 0 |
| 0 | 7,19 | 10 | 0 | 0 | | 0 |
| 0 | 7,27 | -6,1 | 0 | 0 | | 0 |
| 0 | 7,24 | -8,5 | 0 | 0 | | 0 |
| 0 | 7,28 | -3,7 | 0 | 0 | | 0 |
| 0 | 7,20 | 8,8 | 0 | 0 | | 0 |
| 0 | 7,24 | -4,9 | 0 | 0 | | 0 |
| 0 | 7,3 | 1 | 0 | 0 | | 0 |
| 0 | 7,2 | 3,4 | 0 | 0 | | 0 |
| 0 | 7,27 | -5,7 | 0 | 0 | | 0 |
| 0 | 7,14 | -9,3 | 0 | 0 | | 0 |
| 0 | 7,28 | -6,6 | 0 | 1 | 4 | 0 |
| 0 | 7,3 | -5 | 0 | 1 | 2 | 0 |
| 0 | 7,09 | -11,4 | 0 | 0 | | 0 |
| 0 | 7,3 | 6 | 0 | 0 | | 0 |
| 0 | 7,30 | 5,1 | 0 | 0 | | 0 |
| 0 | | | 0 | 0 | | 0 |
| 0 | 7,34 | 5 | 0 | 0 | | 0 |
| 0 | 7,21 | 9 | 0 | 0 | | 0 |
| 0 | 7,18 | 4 | 0 | 0 | | 0 |
| 0 | 7,14 | 5,9 | 0 | 0 | | 0 |
| 0 | 7,25 | 8 | 0 | 0 | | 0 |
| 0 | 7,29 | 2 | 0 | 0 | | 0 |
| 0 | 7,22 | 13,4 | 0 | 0 | | 0 |
| 0 | 7,16 | -8,4 | 0 | 0 | | 0 |
| 0 | 7,05 | -13,7 | 0 | 0 | | 0 |
| 0 | 7,32 | -4,8 | 0 | 0 | | 0 |
| 0 | 7,18 | 9 | 0 | 0 | | 0 |
| 0 | 7,39 | -1,4 | 0 | 0 | | 0 |
| 0 | 7,31 | -4,2 | 0 | 0 | | 0 |
| 0 | 7,27 | -9 | 0 | 0 | | 0 |
| 0 | 7,17 | 8 | 0 | 0 | | 0 |
| 0 | | | | 1 | 8 | 0 |
| 0 | 7,26 | 2 | 0 | 0 | | 0 |
| 0 | 7,27 | 4 | 0 | 0 | | 0 |
| 0 | 7,25 | 7 | 0 | 0 | | 0 |
| 0 | | | | 1 | 1 | 0 |
| 0 | 7,17 | 9,8 | 0 | 0 | | 0 |
| 0 | 7,18 | 6 | 0 | 1 | 7 | 0 |
| 0 | 7,35 | 0,6 | 0 | 1 | 9 | 0 |
| 0 | 7,27 | 8,3 | 0 | 0 | | 0 |
| 0 | | | | 1 | 4 | 0 |
| 0 | 7,31 | -12,4 | 0 | 0 | | 0 |
| 0 | 7,35 | 5,3 | 0 | 0 | | 0 |
| 0 | 7,11 | 9 | 0 | 1 | 3 | 0 |
| 0 | 7,02 | -12,7 | 1 | 0 | | 0 |
| 0 | 7,11 | -9,2 | 0 | 0 | | 0 |
| 0 | 7,25 | 5 | 0 | 0 | | 0 |
| 0 | 7,36 | 1 | 0 | 1 | 4 | 0 |
| 0 | 7,17 | 5 | 0 | 0 | | 0 |
| 0 | 7,15 | -6,6 | 0 | 0 | | 0 |
| 0 | 7,23 | -6,7 | 0 | 0 | | 0 |
| 0 | 7,33 | 5 | 0 | 0 | | 0 |
| 0 | 7,24 | -5,2 | 0 | 0 | | 0 |

| | | | | | | |
|---|---|---|---|---|---|---|
| 0 | 7,25 | -3,2 | 0 | 0 | | 0 |
| 0 | | | | 0 | | 0 |
| 0 | 7,397 | 4,3 | 0 | 1 | 1 | 0 |
| 0 | | | | 0 | | 0 |
| 0 | 7,22 | 3,6 | 0 | 0 | | 0 |
| 0 | | | | 0 | | 0 |
| 0 | 7,18 | 0,4 | 0 | 0 | | 0 |
| 0 | 7,19 | -6 | 0 | 0 | | 0 |
| 0 | 7,21 | -9,7 | 0 | 0 | | 0 |
| 0 | 7,19 | 7,4 | 0 | 0 | | 0 |
| 0 | 7,24 | | | 0 | | 0 |
| 0 | 7,16 | -7,6 | 0 | 0 | | 0 |
| 0 | 7,2 | -7 | 0 | 1 | 1 | 0 |
| 0 | | | | 1 | 4 | 0 |
| 0 | 7,09 | -10,3 | 0 | 0 | | 0 |
| 0 | 7,15 | -8,8 | 0 | 0 | | 0 |
| 0 | 7,33 | -1,9 | 0 | 0 | | 0 |
| 0 | 7,28 | 6 | 0 | 0 | | 0 |
| 0 | 7,17 | 7 | 0 | 0 | | 0 |
| 0 | 7,16 | 4,9 | 0 | 0 | | 0 |
| 0 | 7,21 | 4 | 0 | 0 | | 0 |
| 0 | 7,27 | 1 | 0 | 0 | | 0 |
| 0 | 7,24 | 4 | 0 | 0 | | 0 |
| 0 | 7,28 | -0,8 | 0 | 0 | | 0 |
| 0 | 7,1 | 10 | 0 | 0 | | 0 |
| 0 | 7,39 | -4,3 | 0 | 0 | | 0 |
| 0 | 7,25 | 7,5 | 0 | 0 | | 0 |
| 0 | 7,23 | 8 | 0 | 0 | | 0 |
| 0 | 7,14 | 6,2 | 0 | 0 | | 0 |
| 0 | 7,30 | 0 | 0 | 0 | | 0 |
| 0 | 7,29 | 2 | 0 | 0 | | 0 |
| 0 | 7,28 | -2 | 0 | 0 | | 0 |
| 0 | 7,14 | 9 | 0 | 0 | | 0 |
| 0 | 7,31 | 7,6 | 0 | 0 | | 0 |
| 0 | 7,19 | -8,5 | 0 | 0 | | 0 |
| 0 | 7,31 | 1,8 | 0 | 0 | | 0 |
| 0 | 7,3 | 1 | 0 | 0 | | 0 |
| 0 | 7,32 | 3,7 | 0 | 0 | | 0 |
| 0 | 7,25 | -4,1 | 0 | 1 | 4 | 0 |
| 0 | 7,27 | 7,5 | 0 | 0 | | 0 |
| 0 | 7,24 | 9 | 0 | 0 | | 0 |
| 0 | 7,18 | -8,7 | 0 | 0 | | 0 |
| 0 | 7,06 | 14 | 0 | 1 | 9 | 0 |
| 0 | 7,21 | -6,6 | 0 | 0 | | 0 |
| 0 | 7,36 | 2 | 0 | 0 | | 0 |
| 0 | 7,31 | -3,1 | 0 | 0 | | 0 |
| 0 | 7,23 | 2 | 0 | 0 | | 0 |
| 0 | 7,19 | -9,2 | 0 | 0 | | 0 |
| 0 | 7,26 | 1 | 0 | 0 | | 0 |
| 0 | 7,25 | 7,6 | 0 | 0 | | 0 |
| 0 | 7,36 | -3,5 | 0 | 0 | | 0 |
| 0 | 7,02 | -13 | 0 | 1 | 4 | 0 |
| 0 | 7,17 | -8,6 | 0 | 0 | | 0 |
| 0 | 7,20 | -4,6 | 0 | 0 | | 0 |
| 0 | 6,99 | 11 | 0 | 0 | | 0 |
| 0 | 7,25 | -5 | 0 | 0 | | 0 |
| 0 | 7,32 | 0,5 | 0 | 0 | | 0 |
| 0 | 7,34 | -4,2 | 0 | 0 | | 0 |
| 0 | 7,23 | -8,3 | 0 | 0 | | 0 |

| | | | | | | |
|---|---|---|---|---|---|---|
| 0 | 7,13 | 5 | 0 | 0 | | 0 |
| 0 | 7,15 | 5 | 0 | 0 | | 0 |
| 1 | 7,15 | 7,5 | 0 | 0 | | 0 |
| 0 | 7,19 | -6,4 | 0 | 0 | | 0 |
| 0 | 7,19 | -11,9 | 0 | 0 | | 0 |
| 0 | 7,33 | -2 | 0 | 0 | | 0 |
| 0 | | | | 0 | | 0 |
| 0 | 7,26 | -4,7 | 0 | 0 | | 0 |
| 0 | 7,17 | -9,9 | 0 | 0 | | 0 |
| 1 | 7,26 | -4,1 | 0 | 0 | | 0 |
| 0 | 7,27 | -5 | 0 | 0 | | 0 |
| 0 | 7,20 | -5,8 | 0 | 0 | | 0 |
| 0 | 6,92 | 17,8 | 1 | 0 | | 0 |
| 0 | 7,30 | 5,8 | 0 | 0 | | 0 |
| 0 | 7,13 | 3 | 0 | 0 | | 0 |
| 0 | | | 0 | 0 | | 0 |
| 0 | 7,21 | -4,2 | 0 | 0 | | 0 |
| 0 | 7,18 | -4 | 0 | 0 | | 0 |
| 0 | 7,35 | 0,7 | 0 | 1 | 15 | 0 |
| 0 | 7,2 | 6 | 0 | 0 | | 0 |
| 0 | 7,37 | -1,3 | 0 | 0 | | 0 |
| 0 | 7,23 | 4,7 | 0 | 0 | | 0 |
| 0 | 7,22 | 17 | 0 | 0 | | 0 |
| 0 | 7,3 | 6 | 0 | 0 | | 0 |
| 0 | 7,32 | -1,1 | 0 | 0 | | 0 |
| 0 | 7,11 | -7,6 | 0 | 0 | | 0 |
| 0 | 7,08 | 10,3 | 0 | 0 | | 0 |
| 0 | 7,26 | -0,1 | 0 | 0 | | 0 |
| 0 | | | | 0 | | 0 |
| 0 | 7,28 | 2 | 0 | 1 | 19 | 0 |
| 0 | 7,18 | -8 | 0 | 0 | | 0 |
| 0 | 7,29 | 1,8 | 0 | 0 | | 0 |
| 0 | 7,04 | -10,7 | 0 | 0 | | 0 |
| 0 | 7,15 | -8,6 | 0 | 0 | | 0 |
| 0 | 7,34 | -1,5 | 0 | 0 | | 0 |
| 0 | | | 0 | 0 | | 0 |
| 0 | 7,29 | -6 | 0 | 0 | | 0 |
| 0 | 7,30 | -2 | 0 | 0 | | 0 |
| 0 | | | | 0 | | 0 |
| 0 | 7,27 | -5 | 0 | 0 | | 0 |
| 0 | 7,20 | 6 | 0 | 0 | | 0 |
| 0 | 7,22 | 6,7 | 0 | 0 | | 0 |
| 0 | 7,18 | -6,9 | 0 | 0 | | 0 |
| 0 | 7,25 | -6,3 | 0 | 0 | | 0 |
| 0 | | | | 0 | | 0 |
| 0 | 7,15 | 10 | 0 | 0 | | 0 |
| 0 | 7,28 | 1 | 0 | 0 | | 0 |
| 0 | 7,3 | 4 | 0 | 0 | | 0 |
| 1 | 7,24 | -4,2 | 0 | 0 | | 0 |
| 0 | 7,24 | 8 | 0 | 1 | 22 | 0 |
| 0 | 7,16 | 9 | 0 | 0 | | 0 |
| 0 | 7,32 | 1 | 0 | 0 | | 0 |
| 0 | 7,28 | 2 | 0 | 0 | | 0 |
| 0 | 7,21 | 8 | 0 | 0 | | 0 |
| 0 | 7,33 | 3,8 | 0 | 0 | | 0 |
| 0 | 7,14 | 7 | 0 | 0 | | 0 |
| 0 | | | | 0 | | 0 |
| 0 | 7,14 | 10,6 | 0 | 0 | | 0 |
| 0 | 7,03 | 8 | 0 | 0 | | 0 |

| | | | | | | |
|---|---|---|---|---|---|---|
| 0 | 7,36 | 1,4 | 0 | 0 | | 0 |
| 0 | 7,17 | 6,3 | 0 | 0 | | 0 |
| 0 | 7,10 | 5 | 0 | 0 | | 0 |
| 0 | 7,37 | -4,6 | 0 | 0 | | 0 |
| 0 | 7,24 | -7 | 0 | 0 | | 0 |
| 0 | 7,04 | -13,1 | 0 | 0 | | 0 |
| 0 | 7,22 | 6,6 | 0 | 0 | | 0 |
| 0 | 7,152 | 10,2 | 0 | 0 | | 0 |
| 0 | 7,13 | 3 | 0 | 0 | | 0 |
| 0 | 7,21 | 9 | 0 | 0 | | 0 |
| 0 | 7,28 | -4,8 | 0 | 0 | | 0 |
| 0 | 7,24 | 3 | 0 | 1 | 5 | 0 |
| 0 | 7,34 | -1,3 | 0 | 0 | | 0 |
| 0 | 7,10 | -9,3 | 0 | 0 | | 0 |
| 0 | 7,17 | 11 | 0 | 0 | | 0 |
| 0 | 7,18 | 9 | 0 | 0 | | 0 |
| 0 | 7,15 | 18,1 | 0 | 0 | | 0 |
| 0 | 7,09 | -9,3 | 0 | 0 | | 0 |
| 0 | 7,18 | 8 | 0 | 0 | | 0 |
| 0 | 7,21 | -15 | 0 | 0 | | 0 |
| 0 | | | | 0 | | 0 |
| 0 | 7,3 | -4,2 | 0 | 0 | | 0 |
| 0 | 7,26 | 2 | 0 | 0 | | 0 |
| 0 | 7,18 | 7,1 | 0 | 0 | | 0 |
| 0 | 7,2 | 7 | 0 | 0 | | 0 |
| 1 | 7,25 | -6,7 | 0 | 1 | 7 | 0 |
| 0 | 7,30 | -2,5 | 0 | 0 | | 0 |
| 1 | 6,67 | 28,7 | 1 | 1 | 13 | 3 |
| 0 | 7,17 | 8 | 0 | 1 | 8 | 0 |
| 0 | 7,26 | 3 | 0 | 0 | | 0 |
| 0 | 7,33 | 0,1 | 0 | 0 | | 0 |
| 0 | 7,16 | 7 | 0 | 0 | | 0 |
| 0 | 7,32 | 5 | 0 | 0 | | 0 |
| 0 | 7,1 | 11 | 0 | 0 | | 0 |
| 0 | 7,26 | -3,2 | 0 | 0 | | 0 |
| 0 | 7,09 | 19,4 | 0 | 0 | | 0 |
| 0 | 7,194 | -10 | 0 | 0 | | 0 |
| 0 | 7,22 | -6,7 | 0 | 0 | | 0 |
| 0 | 7,22 | -7,2 | 0 | 0 | | 0 |
| 0 | 7,23 | -3,2 | 0 | 0 | | 0 |
| 0 | 7,22 | -4 | 0 | 0 | | 0 |
| 0 | 7,066 | -15,6 | 0 | 0 | | 0 |
| 0 | 7,25 | 4 | 0 | 0 | | 0 |
| 0 | 7,28 | 0 | 0 | 0 | | 0 |
| 0 | 7,25 | 1 | 0 | 0 | | 0 |
| 0 | 7,09 | 7 | 0 | 0 | | 0 |
| 0 | 7,10 | 11,6 | 0 | 0 | | 0 |
| 0 | 7,3 | 5 | 0 | 0 | | 0 |
| 0 | 6,96 | -16,3 | 1 | 0 | | 0 |
| 0 | 7,24 | 3,3 | 0 | 1 | 1 | 0 |
| 0 | 7,35 | 6,3 | 0 | 0 | | 0 |
| 0 | 7,29 | -3,4 | 0 | 0 | | 0 |
| 0 | 7,27 | 11,9 | 0 | 1 | 1 | 0 |
| 0 | 7,22 | -5,3 | 0 | 0 | | 0 |
| 0 | 7,28 | 3,9 | 0 | 0 | | 0 |
| 0 | 7,34 | 2 | 0 | 0 | | 0 |
| 0 | 7,26 | 2,1 | 0 | 0 | | 0 |
| 0 | 7,25 | 3 | 0 | 0 | | 0 |
| 0 | 7,17 | -7,1 | 0 | 0 | | 0 |

| | | | | | | |
|---|---|---|---|---|---|---|
| 0 | 7,09 | -13 | 0 | 0 | | 0 |
| 0 | | | | 1 | 5 | 0 |
| 0 | 7,31 | -1,7 | 0 | 0 | | 0 |
| 0 | 7,35 | 0 | 0 | 0 | | 0 |
| 1 | 7,27 | 6,5 | 0 | 0 | | 0 |
| 0 | 7,32 | 4 | 0 | 0 | | 0 |
| 0 | 7,16 | 7,7 | 0 | 0 | | 0 |
| 0 | 7,086 | 12,5 | 0 | 0 | | 0 |
| 1 | 7,11 | 12 | 0 | 1 | 11 | 0 |
| 0 | 7,28 | -6,8 | 0 | 0 | | 0 |
| 0 | 7,33 | 2 | 0 | 0 | | 0 |
| 0 | 7,30 | 9 | 0 | 0 | | 0 |
| 0 | 7,25 | 4 | 0 | 0 | | 0 |
| 0 | 7,35 | -3,4 | 0 | 0 | | 0 |
| 0 | | | | 0 | | 0 |
| 0 | 7,21 | 6 | 0 | 0 | | 0 |
| 0 | 7 | -15,7 | 0 | 0 | | 0 |
| 0 | 7,31 | -1 | 0 | 1 | 3 | 0 |
| 0 | 7,11 | 9 | 0 | 1 | 6 | 1 |
| 0 | 7,1 | -9,3 | 0 | 0 | | 0 |
| 0 | 7,27 | -9 | 0 | 0 | | 0 |
| 0 | 7,19 | 5 | 0 | 0 | | 0 |
| 0 | | | | 0 | | 0 |
| 0 | | | | 0 | | 0 |
| 0 | 7,3 | 0 | 0 | 0 | | 0 |
| 0 | 7,19 | -4,2 | 0 | 0 | | 0 |
| 0 | 7,2 | 8,2 | 0 | 0 | | 0 |
| 0 | 7,13 | -6,9 | 0 | 0 | | 0 |
| 0 | 7,24 | -7,9 | 0 | 0 | | 0 |
| 0 | 7,22 | -14,5 | 0 | 0 | | 0 |
| 0 | 7,22 | -8,8 | 0 | 0 | | 0 |
| 0 | 7,42 | -5,5 | 0 | 0 | | 0 |
| 0 | 7,23 | -4,2 | 0 | 0 | | 0 |
| 0 | 7,24 | 10 | 0 | 0 | | 0 |
| 0 | 7,11 | | | 1 | 1 | 0 |
| 0 | 7,16 | -7,6 | 0 | 0 | | 0 |
| 0 | 7,39 | -4,8 | 0 | 0 | | 0 |
| 0 | 7,27 | -2,2 | 0 | 0 | | 0 |
| 0 | | | | 0 | | 0 |
| 0 | 7,12 | 14 | 0 | 0 | | 0 |
| 0 | 7,21 | 5,6 | 0 | 0 | | 0 |
| 0 | 7,14 | -8,7 | 0 | 0 | | 0 |
| 0 | 7,3 | -5,6 | 0 | 0 | | 0 |
| 1 | 7,14 | 7,8 | 0 | 0 | | 0 |
| 0 | | | 0 | 0 | | 0 |
| 0 | 7,23 | -6,4 | 0 | 0 | | 0 |
| 0 | 7,34 | -1,3 | 0 | 0 | | 0 |
| 0 | 7,15 | -10,3 | 0 | 0 | | 0 |
| 0 | 7,15 | 9 | 0 | 0 | | 0 |
| 0 | 7,04 | 11,7 | 0 | 0 | | 0 |
| 1 | 7,19 | 4 | 0 | 1 | 12 | 0 |
| 1 | 7,19 | 13,7 | 0 | 0 | | 0 |
| 0 | 7,17 | 7 | 0 | 1 | 1 | 0 |
| 0 | 7,27 | -3,2 | 0 | 1 | 5 | 0 |
| 0 | 7,30 | -4,2 | 0 | 0 | | 0 |
| 0 | 7,19 | 3,1 | 0 | 0 | | 0 |
| 0 | 7,24 | 6 | 0 | 0 | | 0 |
| 0 | 7,23 | 5,1 | 0 | 0 | | 0 |
| 0 | 7,16 | 6 | | 0 | | 0 |

| | | | | | | |
|---|---|---|---|---|---|---|
| 0 | 7,2 | 8 | 0 | 0 | | 0 |
| 0 | | | 0 | 0 | | 0 |
| 0 | 7,23 | -3,8 | 0 | 0 | | 0 |
| 0 | 7,11 | 12 | 0 | 1 | 1 | 0 |
| 0 | 7,1 | 13 | 0 | 1 | 3 | 0 |
| 0 | 7,33 | 5,9 | 0 | 0 | | 0 |
| 0 | 7,14 | -9,1 | 0 | 0 | | 0 |
| 0 | 7,18 | -3,7 | 0 | 0 | | 0 |
| 0 | 7,21 | -5,4 | 0 | 0 | | 0 |
| 0 | 7,33 | 2 | 0 | 0 | | 0 |
| 0 | 7,38 | 6,1 | 0 | 1 | 1 | 0 |
| 0 | 7,16 | 9 | 0 | 1 | 8 | 0 |
| 0 | 7,15 | -17,9 | 0 | 0 | | 0 |
| 0 | 7,3 | -2,9 | 0 | 0 | | 0 |
| 0 | 7,24 | 4 | 0 | 1 | 1 | 0 |
| 0 | 7,23 | 5,8 | 0 | 1 | 2 | 0 |
| 0 | 7,30 | 6 | 0 | 0 | | 0 |
| 0 | 7,192 | 10 | 0 | 0 | | 0 |
| 0 | 7,19 | 8 | 0 | 1 | 2 | 0 |
| 0 | 7,28 | 6,5 | 0 | 0 | | 0 |
| 0 | | | | 0 | | 0 |
| 0 | 7,15 | -6,8 | 0 | 0 | | 0 |
| 0 | 7,18 | -6 | 0 | 0 | | 0 |
| 0 | 7 | 12,8 | 0 | 0 | | 0 |
| 0 | 7,22 | 4 | 0 | 0 | | 0 |
| 0 | 7,24 | 5 | 0 | 0 | | 0 |
| 0 | 7,22 | -8,9 | 0 | 0 | | 0 |
| 0 | 7,045 | -14,6 | 0 | 0 | | 0 |
| 0 | 7,16 | 10 | 0 | 1 | 2 | 0 |
| 0 | 7,29 | -5 | 0 | 0 | | 0 |
| 1 | 7,24 | -6,5 | 0 | 1 | 4 | 0 |
| 0 | 7,19 | 8 | 0 | 0 | | 0 |
| 0 | 7,18 | 9 | 0 | 0 | | 0 |
| 1 | 7,19 | 6,8 | 0 | 0 | | 0 |
| 0 | 7,21 | 3 | 0 | 0 | | 0 |
| 0 | 7,28 | 5 | 0 | 0 | | 0 |
| 0 | 7,17 | 10 | 0 | 0 | | 0 |
| 0 | 7,05 | 20,2 | 0 | 0 | | 0 |
| 0 | | | | 0 | | 0 |
| 0 | 7,11 | -16,8 | 0 | 0 | | 0 |
| 0 | 7,25 | 6,6 | 0 | 0 | | 0 |
| 0 | 7,2 | 9 | 0 | 0 | | 0 |
| 0 | 7,3 | 3,8 | 0 | 0 | | 0 |
| 0 | 6,92 | 21 | 1 | 0 | | 0 |
| 0 | 7,08 | 17,4 | 0 | 0 | | 0 |
| 0 | 7,21 | 6 | 0 | 0 | | 0 |
| 0 | 7,29 | 4,7 | 0 | 0 | | 0 |
| 0 | 7,19 | 4 | 0 | 0 | | 0 |
| 0 | 7,29 | 6,7 | 0 | 0 | | 0 |
| 0 | 7,18 | | 0 | 0 | | 0 |
| 0 | 7,13 | 8 | 0 | 0 | | 0 |
| 0 | 7,16 | 9 | 0 | 0 | | 0 |
| 0 | 7,21 | -6 | 0 | 0 | | 0 |
| 0 | 7,26 | 4 | 0 | 0 | | 0 |
| 0 | 7,21 | 6,2 | 0 | 0 | | 0 |
| 0 | 7,28 | 4 | 0 | 0 | | 0 |
| 0 | 7,16 | 8,6 | 0 | 0 | | 0 |
| 0 | 7,15 | 7 | 0 | 0 | | 0 |
| 0 | 7,31 | -6,2 | 0 | 0 | | 0 |

| | | | | | | |
|---|---|---|---|---|---|---|
| 0 | 7,25 | 7 | 0 | 0 | | 0 |
| 0 | 7,26 | 4 | 0 | 1 | 6 | 0 |
| 0 | 7,22 | 8 | 0 | 0 | | 0 |
| 0 | 7,23 | 5,4 | 0 | 0 | | 0 |
| 0 | 7,18 | -7,9 | 0 | 0 | | 0 |
| 0 | 7,27 | 4 | 0 | 0 | | 0 |
| 0 | 7,01 | -14,7 | 0 | 0 | | 0 |
| 0 | 7,09 | 10 | 0 | 0 | | 0 |
| 0 | 7,09 | 12 | 0 | 1 | 10 | 0 |
| 0 | 7,17 | -7,7 | 0 | 0 | | 0 |
| 0 | 7,11 | -7 | 0 | 0 | | 0 |
| 0 | 7,22 | 5 | 0 | 0 | | 0 |
| 0 | 7,21 | 5 | 0 | 0 | | 0 |
| 0 | 7,23 | 4 | 0 | 0 | | 0 |
| 0 | 7,22 | 6 | 0 | 0 | | 0 |
| 0 | 7,17 | 7 | 0 | 0 | | 0 |
| 0 | 7,14 | -10,8 | 0 | 0 | | 0 |
| 0 | 7,04 | -13,5 | 0 | 0 | | 0 |
| 0 | 7,267 | -3,8 | 0 | 0 | | 0 |
| 0 | 7,57 | 8,1 | 0 | 0 | | 0 |
| 0 | 7,17 | | | 1 | 19 | 0 |
| 0 | 7,26 | -7,4 | 0 | 0 | | 0 |
| 0 | 7,37 | 3,2 | 0 | 0 | | 0 |
| 0 | 7,05 | 12 | 0 | 0 | | 0 |
| 0 | 7,24 | 4,9 | 0 | 0 | | 0 |
| 0 | 7,30 | 5 | 0 | 0 | | 0 |
| 0 | 7,24 | 7 | 0 | 0 | | 0 |
| 0 | 7,12 | 8,9 | 0 | 0 | | 0 |
| 0 | 7,29 | 1 | 0 | 0 | | 0 |
| 0 | 7,17 | 7 | 0 | 0 | | 0 |
| 0 | 7,37 | 3,2 | 0 | 0 | | 0 |
| 0 | 7,18 | 8 | 0 | 0 | | 0 |
| 0 | 7,39 | -3,2 | 0 | 0 | | 0 |
| 0 | 7,06 | -13,3 | 0 | 0 | | 0 |
| 0 | 7,15 | 7 | 0 | 0 | | 0 |
| 0 | 7,29 | 3 | 0 | 0 | | 0 |
| 0 | 7,24 | -6,7 | 0 | 0 | | 0 |
| 0 | 7,08 | 11,6 | 0 | 0 | | 0 |
| 0 | 7,29 | 5 | 0 | 0 | | 0 |
| 0 | 7,16 | 9,3 | 0 | 0 | | 0 |
| 0 | | | | 0 | | 0 |
| 0 | 7,18 | -9 | 0 | 1 | 3 | 0 |
| 0 | 7,095 | -8,8 | 0 | 0 | | 0 |
| 0 | 7,26 | 9 | 0 | 0 | | 0 |
| 0 | 7,16 | 11,1 | 0 | 1 | 26 | 0 |
| 0 | 7,24 | 13,1 | 0 | 0 | | 0 |
| 0 | 7,22 | 8,1 | 0 | 0 | | 0 |
| 0 | 7,04 | 13,8 | 1 | 0 | | 0 |
| 0 | 7,26 | 4,3 | 0 | 0 | | 0 |
| 0 | 6,93 | 17 | 1 | 0 | | 0 |
| 0 | 7,2 | 7 | 0 | 0 | | 0 |
| 0 | 7,04 | 11 | 0 | 0 | | 0 |
| 0 | 7,35 | 3 | 0 | 1 | 1 | 0 |
| 0 | 7,20 | -8,7 | 0 | 0 | | 0 |
| 0 | 7,12 | 9 | 0 | 0 | | 0 |
| 0 | 7,17 | 7,3 | 0 | 0 | | 0 |
| 0 | 7,25 | 3,9 | 0 | 0 | | 0 |
| 0 | 7,33 | -2,7 | 0 | 0 | | 0 |
| 0 | 7,15 | 7,8 | 0 | 0 | | 0 |

| | | | | | | |
|---|---|---|---|---|---|---|
| 0 | 6,87 | 26 | 1 | 1 | 6 | 2 |
| 0 | 7,31 | -7,6 | 0 | 0 | | 0 |
| 0 | 7,2 | 3 | 0 | 0 | | 0 |
| 0 | 7,09 | 11 | 0 | 0 | | 0 |
| 0 | 7,2 | -7 | 0 | 0 | | 0 |
| 0 | 7,24 | -6,9 | 0 | 0 | | 0 |
| 0 | 7,2 | -7 | 0 | 0 | | 0 |
| 1 | 7,21 | -7,6 | 0 | 1 | 2 | 0 |
| 0 | 7,16 | -8,4 | 0 | 0 | | 0 |
| 0 | 7,32 | -3 | 0 | 0 | | 0 |
| 0 | 7,24 | -4,3 | 0 | 1 | 5 | 0 |
| 0 | 6,94 | -14,3 | 1 | 0 | | 0 |
| 0 | 7,22 | 3 | 0 | 0 | | 0 |
| 0 | 7,33 | -6 | 0 | 0 | | 0 |
| 1 | 7,1 | 9 | 0 | 1 | 4 | 0 |
| 0 | 7,15 | -11 | 0 | 1 | 3 | 0 |
| 0 | 7,34 | -1,1 | 0 | 0 | | 0 |
| 0 | 7,21 | 7 | 0 | 0 | | 0 |
| 0 | 7,129 | -9,6 | 0 | 1 | 3 | 0 |
| 0 | 7,3 | -6,7 | 0 | 0 | | 0 |
| 0 | 7,34 | -8,2 | 0 | 0 | | 0 |
| 0 | 7,21 | 5,4 | 0 | 0 | | 0 |
| 0 | 7,25 | 2 | 0 | 0 | | 0 |
| 0 | 7,24 | 2 | 0 | 0 | | 0 |
| 0 | 7,26 | 7 | 0 | 0 | | 0 |
| 0 | 7,15 | 8 | 0 | 1 | 10 | 0 |
| 0 | 7,12 | 11 | 0 | 1 | 14 | 0 |
| 0 | | | 0 | 0 | | 0 |
| 0 | 7,21 | 4 | 0 | 0 | | 0 |
| 0 | 7,36 | 2 | 0 | 0 | | 0 |
| 0 | 7,13 | 7 | 0 | 0 | | 0 |
| 0 | 7,25 | 11,4 | 0 | 0 | | 0 |
| 0 | 7,39 | -5,5 | 0 | 0 | | 0 |
| 0 | 7,24 | -6 | 0 | 0 | | 0 |
| 0 | 7,22 | -4,6 | 0 | 0 | | 0 |
| 0 | 7,29 | -2 | 0 | 1 | 6 | 0 |
| 0 | 7,25 | | 0 | 0 | | 0 |
| 0 | 7,37 | -4,9 | 0 | 0 | | 0 |
| 0 | | | | 0 | | 0 |
| 0 | 7,20 | 6,7 | | 0 | | 0 |
| 0 | 7,18 | -5,8 | 0 | 0 | | 0 |
| 0 | 7,14 | 7,7 | 0 | 0 | | 0 |
| 0 | 7,15 | 13 | 0 | 0 | | 0 |
| 0 | 7,25 | -2,4 | 0 | 0 | | 0 |
| 0 | 7,27 | 3,4 | 0 | 0 | | 0 |
| 0 | 7,40 | -4,5 | 0 | 0 | | 0 |
| 0 | 7,15 | 5,5 | 0 | 0 | | 0 |
| 0 | 7,33 | -1,6 | 0 | 0 | | 0 |
| 0 | 7,19 | -6 | 0 | 1 | 3 | 0 |
| 0 | 7,20 | 11 | 0 | 0 | | 0 |
| 0 | 7,22 | 4,5 | 0 | 0 | | 0 |
| 0 | 7,2 | -4 | 0 | 1 | 4 | 0 |
| 0 | 7,31 | -2 | 0 | 1 | 1 | 0 |
| 0 | 7,41 | -2 | 0 | 0 | | 0 |
| 0 | 7,23 | 8 | 0 | 0 | | 0 |
| 0 | 7,25 | -4,6 | 0 | 1 | 10 | 0 |
| 1 | 6,95 | 19 | 0 | 1 | 8 | 0 |
| 0 | 7,28 | -5 | 0 | 0 | | 0 |
| 0 | 7,19 | 9 | 0 | 1 | 9 | 0 |

| | | | | | | |
|---|---|---|---|---|---|---|
| 0 | 7,06 | 13 | 0 | 1 | 2 | 0 |
| 0 | 7,18 | 12 | 0 | 0 | | 0 |
| 0 | 7,24 | -3,6 | 0 | 0 | | 0 |
| 0 | 7,35 | -1,9 | 0 | 0 | | 0 |
| 0 | 7,20 | 8,3 | 0 | 0 | | 0 |
| 0 | 7,10 | -10,2 | 0 | 1 | 5 | 0 |
| 0 | 7,43 | -3,7 | 0 | 0 | | 0 |
| 0 | 7,3 | -4,3 | 0 | 0 | | 0 |
| 0 | 7,148 | -8 | 0 | 0 | | 0 |
| 0 | 7,27 | 2 | 0 | 0 | | 0 |
| 0 | 7,14 | -8,1 | 1 | 1 | 11 | 0 |
| 0 | 7,30 | -5,7 | 0 | 0 | | 0 |
| 0 | 7,21 | 5,4 | 0 | 0 | | 0 |
| 0 | 7,24 | | | 0 | | 0 |
| 0 | 7,14 | 10 | 0 | 0 | | 0 |
| 0 | 7,1 | -12,1 | 0 | 0 | | 0 |
| 0 | 7,34 | 2 | 0 | 0 | | 0 |
| 0 | 7,20 | 5 | 0 | 1 | 4 | 0 |
| 0 | 7,15 | -10,3 | 0 | 0 | | 0 |
| 0 | 7,14 | 10 | 0 | 0 | | 0 |
| 0 | 7,22 | 5 | 0 | 0 | | 0 |
| 0 | 7,16 | 8,7 | 0 | 0 | | 0 |
| 0 | 7,14 | -10,5 | 0 | 0 | | 0 |
| 0 | 7,07 | -11,7 | 0 | 0 | | 0 |
| 0 | 7,20 | 5,4 | 0 | 0 | | 0 |
| 0 | 7,07 | -15,6 | 0 | 0 | | 0 |
| 0 | 7,1 | -9 | 0 | 1 | 2 | 0 |
| 0 | 7,34 | -4,6 | 0 | 0 | | 0 |
| 0 | 7,40 | -3,6 | 0 | 0 | | 0 |
| 0 | 7,25 | 5 | 0 | 1 | 3 | 0 |
| 0 | 7,22 | -4,1 | 0 | 1 | 3 | 0 |
| 0 | 7,31 | 6 | 0 | 0 | | 0 |
| 0 | 7,24 | 2 | 0 | 0 | | 0 |
| 0 | 7,20 | 11,4 | 0 | 0 | | 0 |
| 0 | 7,31 | -0,8 | 0 | 0 | | 0 |
| 0 | 7,37 | -1,8 | 0 | 0 | | 0 |
| 0 | 7,18 | -7,9 | 0 | 0 | | 0 |
| 0 | 7,19 | -7,8 | 0 | 0 | | 0 |
| 1 | 7,15 | | | 0 | | 0 |
| 0 | 7,04 | 8,6 | 0 | 0 | | 0 |
| 0 | 7,34 | -8,2 | 0 | 0 | | 0 |
| 0 | 7,18 | 8 | 0 | 0 | | 0 |
| 0 | 7,10 | 9 | 0 | 1 | 1 | 0 |
| 0 | 7,32 | -4,7 | 0 | 0 | | 0 |
| 0 | 7,14 | 6 | 0 | 0 | | 0 |
| 0 | 7,13 | 12 | 0 | 0 | | 0 |
| 0 | 7,33 | 3 | 0 | 0 | | 0 |
| 0 | 7,25 | 3 | 0 | 0 | | 0 |
| 0 | 7,29 | 1 | 0 | 0 | | 0 |
| 0 | 7,08 | -10,2 | 0 | 0 | | 0 |
| 0 | | -11 | 0 | 1 | 9 | 0 |
| 0 | | | | 0 | | 0 |
| 0 | 7,29 | -3,6 | 0 | 0 | | 0 |
| | | | | | | |
| 0 | 7,311 | 5,2 | 0 | 0 | | 0 |
| | | | | | | |
| 0 | 7,16 | -8,2 | 0 | 0 | | 0 |
| 1 | 7,26 | 7 | 0 | 0 | | 0 |
| | | | | | | |

| | | | | | | |
|---|---|---|---|---|---|---|
| 0 | 7,14 | 6 | 0 | 0 | | 0 |
| 0 | 6,88 | 18 | 1 | 1 | 15 | 0 |
| 0 | 7,05 | -11,9 | 0 | 0 | | 0 |
| 0 | 7,14 | -10,1 | 0 | 0 | | 0 |
| | | | | | | |
| 0 | 7,28 | 7,3 | 0 | 0 | | 0 |
| 0 | 7,24 | 6 | 0 | 0 | | 0 |
| | | | | | | |
| 0 | 7,16 | 6 | 0 | 1 | 8 | 0 |
| | | | | | | |
| 0 | 7,2 | 5 | 0 | 0 | | 0 |
| 0 | 7,22 | 7 | 0 | 0 | | 0 |
| 0 | 7,19 | 5,9 | 0 | 0 | | 0 |
| 0 | 7,22 | 6 | 0 | 1 | 2 | 0 |
| | | | | | | |
| 0 | 7,27 | -3,5 | 0 | 0 | | 0 |
| 0 | 7,31 | 2,2 | 0 | 0 | | 0 |
| 0 | 7,29 | -2,6 | 0 | 0 | | 0 |
| 0 | 7,27 | 0 | 0 | 0 | | 0 |
| | | | | | | |
| | | | | | | |
| 0 | 7,28 | 2 | 0 | 0 | | 0 |
| 0 | 7,02 | 9 | 0 | 0 | | 0 |
| 0 | 7,27 | -5 | 0 | 0 | | 0 |
| 0 | 7,21 | -5,4 | 0 | 0 | | 0 |
| | | | | | | |
| | | | | | | |
| | | | | | | |
| 0 | 7,11 | 9 | 0 | 1 | 6 | 2 |
| 0 | 7,26 | 5 | 0 | 0 | | 0 |
| 0 | 7,22 | 7 | 0 | 0 | | 0 |

| MAS | Andning | CPAP | Resp | Grovmoto | Finmotorik | else instrul | Syn | Hörsel |
|---|---|---|---|---|---|---|---|---|
| 0 | 0 | 0 | 0 | | | | | |
| 0 | 0 | 0 | 0 | | | | | |
| 0 | 0 | 0 | 0 | | | | | |
| 0 | 0 | 0 | 0 | | | | | |
| 0 | 0 | 0 | 0 | | | | | |
| 0 | 0 | 0 | 0 | | | | | |
| 0 | 1 | 1 | 0 | | | | | |
| 0 | 0 | 0 | 0 | | | | | |
| 0 | 0 | 0 | 0 | | | | | |
| 0 | 0 | 0 | 0 | | | | | |
| 0 | 0 | 0 | 0 | | | | | |
| 0 | 0 | 0 | 0 | | | | | |
| 0 | 0 | 0 | 0 | | | | | |
| 0 | 0 | 0 | 0 | | | | | |
| 0 | 0 | 0 | 0 | | | | | |
| 0 | 0 | 0 | 0 | | | | | |
| 0 | 0 | 0 | 0 | | | | | |
| 0 | 0 | 0 | 0 | | | | | |
| 0 | 0 | 0 | 0 | | | | | |
| 0 | 0 | 0 | 0 | | | | | |
| 0 | 0 | 0 | 0 | | | | | |
| 0 | 0 | 0 | 0 | | | | | |
| 0 | 0 | 0 | 0 | | | | | |
| 0 | 0 | 0 | 0 | | | | | |
| 0 | 0 | 0 | 0 | | | | | |
| 0 | 0 | 0 | 0 | | | | | |
| 0 | 0 | 0 | 0 | | | | | |
| 0 | 0 | 0 | 0 | | | | | |
| 0 | 0 | 0 | 0 | | | | | |
| 0 | 0 | 0 | 0 | | | | | |
| 0 | 0 | 0 | 0 | | | | | |
| 0 | 0 | 0 | 0 | | | | | |
| 0 | 0 | 0 | 0 | | | | | |
| 0 | 0 | 0 | 0 | | | | | |
| 0 | 0 | 0 | 0 | | | | | |
| 0 | 0 | 0 | 0 | | | | | |
| 0 | 0 | 0 | 0 | | | | | |
| 0 | 0 | 0 | 0 | | | | | |
| 0 | 0 | 0 | 0 | | | | | |
| 0 | 0 | 0 | 0 | | | | | |
| 0 | 0 | 0 | 0 | | | | | |
| 0 | 0 | 0 | 0 | | | | | |
| 0 | 0 | 0 | 0 | | | | | |
| 0 | 0 | 0 | 0 | | | | | |
| 0 | 0 | 0 | 0 | | | | | |
| 0 | 0 | 0 | 0 | | | | | |
| 0 | 0 | 0 | 0 | | | | | |
| 0 | 0 | 0 | 0 | | | | | |
| 0 | 0 | 0 | 0 | | | | | |
| 0 | 0 | 0 | 0 | | | | | |
| 0 | 0 | 0 | 0 | | | | | |
| 0 | 0 | 0 | 0 | | | | | |
| 0 | 0 | 0 | 0 | | | | | |
| 0 | 0 | 0 | 0 | | | | | |
| 0 | 0 | 0 | 0 | | | | | |
| 0 | 0 | 0 | 0 | | | | | |
| 0 | 0 | 0 | 0 | | | | | |
| 0 | 0 | 0 | 0 | | | | | |

| | | | | | | | | |
|---|---|---|---|---|---|---|---|---|
| 0 | 0 | 0 | 0 | | | | | |
| 0 | 0 | 0 | 0 | | | | | |
| 0 | 0 | 0 | 0 | | | | | |
| 0 | 0 | 0 | 0 | | | | | |
| 0 | 0 | 0 | 0 | | | | | |
| 0 | 0 | 0 | 0 | | | | | |
| 0 | 0 | 0 | 0 | | | | | |
| 0 | 0 | 0 | 0 | | | | | |
| 0 | 0 | 0 | 0 | | | | | |
| 0 | 0 | 0 | 0 | | | | | |
| 0 | 0 | 0 | 0 | | | | | |
| 0 | 0 | 0 | 0 | | | | | |
| 0 | 0 | 0 | 0 | | | | | |
| 0 | 0 | 0 | 0 | | | | | |
| 0 | 0 | 0 | 0 | | | | | |
| 0 | 0 | 0 | 0 | | | | | |
| 0 | 0 | 0 | 0 | | | | | |
| 0 | 0 | 0 | 0 | | | | | |
| 0 | 0 | 0 | 0 | | | | | |
| 0 | 0 | 0 | 0 | | | | | |
| 0 | 0 | 0 | 0 | | | | | |
| 0 | 0 | 0 | 0 | | | | | |
| 0 | 0 | 0 | 0 | | | | | |
| 0 | 0 | 0 | 0 | | | | | |
| 0 | 0 | 0 | 0 | | | | | |
| 0 | 0 | 0 | 0 | | | | | |
| 0 | 0 | 0 | 0 | | | | | |
| 0 | 0 | 0 | 0 | | | | | |
| 0 | 0 | 0 | 0 | | | | | |
| 0 | 0 | 0 | 0 | | | | | |
| 0 | 1 | 1 | 0 | | | | | |
| 0 | 0 | 0 | 0 | | | | | |
| 0 | 0 | 0 | 0 | | | | | |
| 0 | 0 | 0 | 0 | | | | | |
| 0 | 0 | 0 | 0 | | | | | |
| 0 | 0 | 0 | 0 | | | | | |
| 0 | 0 | 0 | 0 | | | | | |
| 0 | 0 | 0 | 0 | | | | | |
| 0 | 0 | 0 | 0 | | | | | |
| 0 | 0 | 0 | 0 | | | | | |
| 0 | 0 | 0 | 0 | | | | | |
| 0 | 0 | 0 | 0 | | | | | |
| 0 | 0 | 0 | 0 | | | | | |
| 0 | 0 | 0 | 0 | | | | | |
| 0 | 1 | 1 | 0 | | | | | |
| 0 | 0 | 0 | 0 | | | | | |
| 0 | 1 | 1 | 0 | | | | | |
| 0 | 0 | 0 | 0 | | | | | |
| 0 | 0 | 0 | 0 | | | | | |
| 0 | 0 | 0 | 0 | | | | | |
| 0 | 0 | 0 | 0 | | | | | |
| 0 | 0 | 0 | 0 | | | | | |
| 0 | 0 | 0 | 0 | | | | | |
| 0 | 0 | 0 | 0 | | | | | |
| 0 | 0 | 0 | 0 | | | | | |
| 0 | 0 | 0 | 0 | | | | | |
| 0 | 0 | 0 | 0 | | | | | |
| 0 | 0 | 0 | 0 | | | | | |
| 0 | 0 | 0 | 0 | | | | | |

| | | | | | | | | |
|---|---|---|---|---|---|---|---|---|
| 0 | 0 | 0 | 0 | | | | | |
| 0 | 0 | 0 | 0 | | | | | |
| 0 | 0 | 0 | 0 | | | | | |
| 0 | 0 | 0 | 0 | | | | | |
| 0 | 0 | 0 | 0 | | | | | |
| 0 | 0 | 0 | 0 | | | | | |
| 0 | 0 | 0 | 0 | | | | | |
| 0 | 0 | 0 | 0 | | | | | |
| 0 | 0 | 0 | 0 | | | | | |
| 0 | 0 | 0 | 0 | | | | | |
| 0 | 0 | 0 | 0 | | | | | |
| 0 | 0 | 0 | 0 | | | | | |
| 0 | 0 | 0 | 0 | | | | | |
| 0 | 1 | 1 | 0 | | | | | |
| 0 | 0 | 0 | 0 | | | | | |
| 0 | 0 | 0 | 0 | | | | | |
| 0 | 0 | 0 | 0 | | | | | |
| 0 | 0 | 0 | 0 | | | | | |
| 0 | 0 | 0 | 0 | | | | | |
| 0 | 0 | 0 | 0 | | | | | |
| 0 | 0 | 0 | 0 | | | | | |
| 0 | 0 | 0 | 0 | | | | | |
| 0 | 0 | 0 | 0 | | | | | |
| 0 | 0 | 0 | 0 | | | | | |
| 0 | 0 | 0 | 0 | | | | | |
| 0 | 0 | 0 | 0 | | | | | |
| 0 | 0 | 0 | 0 | | | | | |
| 0 | 0 | 0 | 0 | | | | | |
| 0 | 0 | 0 | 0 | | | | | |
| 0 | 0 | 0 | 0 | | | | | |
| 0 | 0 | 0 | 0 | | | | | |
| 0 | 0 | 0 | 0 | | | | | |
| 0 | 0 | 0 | 0 | | | | | |
| 0 | 0 | 0 | 0 | | | | | |
| 0 | 0 | 0 | 0 | | | | | |
| 0 | 0 | 0 | 0 | | | | | |
| 0 | 0 | 0 | 0 | | | | | |
| 0 | 0 | 0 | 0 | | | | | |
| 0 | 0 | 0 | 0 | | | | | |
| 0 | 0 | 0 | 0 | | | | | |
| 0 | 0 | 0 | 0 | | | | | |
| 0 | 0 | 0 | 0 | | | | | |
| 0 | 0 | 0 | 0 | | | | | |
| 0 | 0 | 0 | 0 | | | | | |
| 0 | 0 | 0 | 0 | | | | | |
| 0 | 0 | 0 | 0 | | | | | |
| 0 | 0 | 0 | 0 | | | | | |
| 0 | 0 | 0 | 0 | | | | | |
| 0 | 0 | 0 | 0 | | | | | |
| 0 | 0 | 0 | 0 | | | | | |
| 0 | 0 | 0 | 0 | | | | | |
| 0 | 0 | 0 | 0 | | | | | |
| 0 | 0 | 0 | 0 | | | | | |
| 0 | 0 | 0 | 0 | | | | | |
| 0 | 0 | 0 | 0 | | | | | |
| 0 | 0 | 0 | 0 | | | | | |
| 0 | 0 | 0 | 0 | | | | | |
| 0 | 0 | 0 | 0 | | | | | |
| 0 | 0 | 0 | 0 | | | | | |

| | | | | | | | | |
|---|---|---|---|---|---|---|---|---|
| 0 | 0 | 0 | 0 | | | | | |
| 0 | 0 | 0 | 0 | | | | | |
| 0 | 0 | 0 | 0 | | | | | |
| 0 | 0 | 0 | 0 | | | | | |
| 0 | 0 | 0 | 0 | | | | | |
| 0 | 0 | 0 | 0 | | | | | |
| 0 | 0 | 0 | 0 | | | | | |
| 0 | 0 | 0 | 0 | | | | | |
| 0 | 0 | 0 | 0 | | | | | |
| 0 | 0 | 0 | 0 | | | | | |
| 0 | 0 | 0 | 0 | | | | | |
| 0 | 0 | 0 | 0 | | | | | |
| 0 | 0 | 0 | 0 | | | | | |
| 0 | 0 | 0 | 0 | | | | | |
| 0 | 0 | 0 | 0 | | | | | |
| 0 | 0 | 0 | 0 | | | | | |
| 0 | 0 | 0 | 0 | | | | | |
| 0 | 0 | 0 | 0 | | | | | |
| 0 | 0 | 0 | 0 | | | | | |
| 0 | 0 | 0 | 0 | | | | | |
| 0 | 0 | 0 | 0 | | | | | |
| 0 | 0 | 0 | 0 | | | | | |
| 0 | 0 | 0 | 0 | | | | | |
| 0 | 0 | 0 | 0 | | | | | |
| 0 | 0 | 0 | 0 | | | | | |
| 0 | 0 | 0 | 0 | | | | | |
| 0 | 0 | 0 | 0 | | | | | |
| 0 | 0 | 0 | 0 | | | | | |
| 0 | 0 | 0 | 0 | | | | | |
| 0 | 0 | 0 | 0 | | | | | |
| 0 | 0 | 0 | 0 | | | | | |
| 0 | 0 | 0 | 0 | | | | | |
| 0 | 0 | 0 | 0 | | | | | |
| 0 | 0 | 0 | 0 | | | | | |
| 0 | 0 | 0 | 0 | | | | | |
| 0 | 0 | 0 | 0 | | | | | |
| 0 | 0 | 0 | 0 | | | | | |
| 0 | 0 | 0 | 0 | | | | | |
| 0 | 0 | 0 | 0 | | | | | |
| 0 | 0 | 0 | 0 | | | | | |
| 0 | 0 | 0 | 0 | | | | | |
| 0 | 0 | 0 | 0 | | | | | |
| 0 | 0 | 0 | 0 | | | | | |
| 0 | 0 | 0 | 0 | | | | | |
| 0 | 0 | 0 | 0 | | | | | |
| 0 | 0 | 0 | 0 | | | | | |
| 0 | 0 | 0 | 0 | | | | | |
| 0 | 0 | 0 | 0 | | | | | |
| 0 | 0 | 0 | 0 | | | | | |
| 0 | 0 | 0 | 0 | 0 | 0 | 0 | 0 | 0 |
| 0 | 0 | 0 | 0 | 0 | 0 | 0 | 0 | 0 |
| 0 | 0 | 0 | 0 | 0 | 0 | 0 | 0 | 0 |
| 0 | 0 | 0 | 0 | 0 | 0 | 0 | 0 | 0 |
| 0 | 0 | 0 | 0 | 0 | 0 | 0 | 0 | 0 |
| 0 | 0 | 0 | 0 | 0 | 0 | 0 | 0 | 0 |
| 0 | 0 | 0 | 0 | 0 | 0 | 0 | 0 | 0 |
| 0 | 0 | 0 | 0 | 0 | 0 | 0 | 0 | 0 |
| 0 | 0 | 0 | 0 | 0 | 0 | 0 | 0 | 0 |
| 0 | 0 | 0 | 0 | 0 | 0 | 0 | 0 | 0 |

| | | | | | | | | |
|---|---|---|---|---|---|---|---|---|
| 0 | 0 | 0 | 0 | 0 | 0 | 0 | 0 | 0 |
| 0 | 0 | 0 | 0 | 0 | 0 | 0 | 0 | 0 |
| 0 | 0 | 0 | 0 | 0 | 0 | 0 | 0 | 0 |
| 0 | 0 | 0 | 0 | 0 | 0 | 0 | 0 | 0 |
| 0 | 0 | 0 | 0 | 0 | 0 | 0 | 1 | 0 |
| 0 | 0 | 0 | 0 | 0 | 0 | 0 | 0 | 0 |
| 0 | 0 | 0 | 0 | 0 | 0 | 0 | 1 | 0 |
| 0 | 0 | 0 | 0 | 0 | 0 | 0 | 1 | 0 |
| 0 | 0 | 0 | 0 | 0 | 0 | 0 | 0 | 0 |
| 0 | 0 | 0 | 0 | 0 | 0 | 0 | 0 | 0 |
| 0 | 0 | 0 | 0 | 0 | 0 | 0 | 0 | 0 |
| 0 | 0 | 0 | 0 | 0 | 0 | 0 | 0 | 0 |
| 0 | 0 | 0 | 0 | 0 | 0 | 0 | 0 | 0 |
| 0 | 0 | 0 | 0 | 0 | 0 | 0 | 0 | 0 |
| 0 | 0 | 0 | 0 | 0 | 0 | 0 | 1 | 0 |
| 0 | 0 | 0 | 0 | 0 | 0 | 0 | 0 | 0 |
| 0 | 1 | 1 | 0 | 0 | 0 | 0 | 0 | 0 |
| 0 | 0 | 0 | 0 | 0 | 0 | 0 | 0 | 0 |
| 0 | 0 | 0 | 0 | 0 | 0 | 0 | 0 | 0 |
| 0 | 0 | 0 | 0 | 0 | 0 | 0 | 0 | 0 |
| 0 | 0 | 0 | 0 | 1 | 0 | 0 | 0 | 0 |
| 0 | 0 | 0 | 0 | 0 | 0 | 0 | 1 | 0 |
| 0 | 0 | 0 | 0 | 0 | 0 | 0 | 0 | 0 |
| 0 | 0 | 0 | 0 | 0 | 0 | | 0 | 0 |
| 0 | 0 | 0 | 0 | 0 | 0 | 0 | 0 | 0 |
| 0 | 1 | 0 | 1 | 0 | 0 | 0 | 0 | 0 |
| 0 | 0 | 0 | 0 | 0 | 0 | 0 | 0 | 0 |
| 0 | 1 | 0 | 1 | 1 | 1 | 1 | 1 | 1 |
| 1 | 0 | 0 | 0 | 0 | 0 | 0 | 0 | 0 |
| 0 | 0 | 0 | 0 | 0 | 0 | 0 | 0 | 0 |
| 0 | 0 | 0 | 0 | 0 | 0 | 0 | 0 | 0 |
| 0 | 0 | 0 | 0 | 0 | 0 | 0 | 0 | 0 |
| 0 | 0 | 0 | 0 | 0 | 0 | 0 | 0 | 0 |
| 0 | 0 | 0 | 0 | 0 | 0 | 0 | 0 | 0 |
| 0 | 0 | 0 | 0 | 0 | 0 | 0 | 0 | 0 |
| 0 | 0 | 0 | 0 | 0 | 0 | 0 | 0 | 0 |
| 0 | 0 | 0 | 0 | 0 | 0 | 0 | 0 | 0 |
| 0 | 0 | 0 | 0 | 0 | 0 | 0 | 0 | 0 |
| 0 | 0 | 0 | 0 | 0 | 0 | 0 | 0 | 0 |
| 0 | 0 | 0 | 0 | 0 | 0 | 0 | 0 | 0 |
| 0 | 0 | 0 | 0 | 0 | 0 | 0 | 0 | 0 |
| 0 | 0 | 0 | 0 | 0 | 0 | 0 | 0 | 0 |
| 0 | 0 | 0 | 0 | 0 | 0 | 0 | 1 | 0 |
| 0 | 0 | 0 | 0 | 0 | 1 | 0 | 1 | 0 |
| 0 | 0 | 0 | 0 | 0 | 0 | 0 | 0 | |
| 0 | 0 | 0 | 0 | 0 | 0 | 1 | 0 | 0 |
| 0 | 0 | 0 | 0 | 0 | 0 | 0 | 0 | 0 |
| 0 | 0 | 0 | 0 | 0 | 0 | 0 | 0 | 0 |
| 0 | 0 | 0 | 0 | 0 | 0 | 0 | 0 | 0 |
| 0 | 0 | 0 | 0 | 0 | 0 | 0 | 0 | 0 |
| 0 | 0 | 0 | 0 | 0 | 0 | 0 | 0 | 0 |
| 0 | 0 | 0 | 0 | 0 | 0 | 0 | 0 | 0 |
| 0 | 0 | 0 | 0 | 0 | 0 | 0 | 0 | 0 |
| 0 | 0 | 0 | 0 | 0 | 0 | 0 | 0 | 0 |
| 0 | 0 | 0 | 0 | 0 | 0 | 0 | 0 | 0 |
| 0 | 0 | 0 | 0 | 0 | 0 | 0 | 0 | 0 |
| 0 | 0 | 0 | 0 | 0 | 0 | 0 | 0 | 0 |
| 0 | 0 | 0 | 0 | 0 | 0 | 0 | 1 | 0 |
| 0 | 0 | 0 | 0 | 0 | 0 | 0 | 0 | 0 |

| | | | | | | | | |
|---|---|---|---|---|---|---|---|---|
| 0 | 0 | 0 | 0 | 0 | 0 | | 0 | 0 |
| 0 | 0 | 0 | 0 | 0 | 0 | 0 | 0 | 0 |
| 0 | 0 | 0 | 0 | 0 | 0 | 0 | 0 | 0 |
| 0 | 0 | 0 | 0 | 0 | 0 | 0 | 0 | 0 |
| 0 | 0 | 0 | 0 | 0 | 0 | 0 | 0 | 0 |
| 0 | 0 | 0 | 0 | 0 | 1 | 0 | 0 | 0 |
| 0 | 0 | 0 | 0 | 0 | 0 | 0 | 0 | 0 |
| 0 | 0 | 0 | 0 | 0 | 0 | 0 | 0 | 0 |
| 1 | 1 | 0 | 1 | 1 | 1 | 1 | 1 | 0 |
| 0 | 0 | 0 | 0 | 0 | 0 | 0 | 0 | 0 |
| 0 | 0 | 0 | 0 | 0 | 0 | 0 | 0 | 0 |
| 0 | 0 | 0 | 0 | 0 | 0 | 0 | 0 | 0 |
| 0 | 0 | 0 | 0 | 0 | 0 | 0 | 0 | 0 |
| 0 | 0 | 0 | 0 | 0 | 0 | 0 | 0 | 0 |
| 0 | 0 | 0 | 0 | 0 | 0 | 0 | 0 | 0 |
| 0 | 0 | 0 | 0 | 0 | 0 | 0 | 0 | 0 |
| 0 | 0 | 0 | 0 | 0 | 0 | 0 | 0 | 0 |
| 0 | 0 | 0 | 0 | 0 | 0 | 0 | 0 | 0 |
| 0 | 0 | 0 | 0 | 0 | 0 | 0 | 0 | 0 |
| 0 | 0 | 0 | 0 | 0 | 0 | 0 | 0 | 0 |
| 0 | 0 | 0 | 0 | 0 | 0 | 0 | 0 | 0 |
| 0 | 0 | 0 | 0 | 0 | 0 | 0 | 0 | 0 |
| 0 | 0 | 0 | 0 | 0 | 0 | 0 | 0 | 0 |
| 0 | 0 | 0 | 0 | 0 | 0 | 0 | 0 | 0 |
| 0 | 0 | 0 | 0 | 0 | 0 | 0 | 0 | 0 |
| 0 | 0 | 0 | 0 | 0 | 0 | 0 | 0 | 0 |
| 0 | 0 | 0 | 0 | 0 | 0 | 0 | 1 | 0 |
| 0 | 0 | 0 | 0 | 0 | 0 | 0 | 0 | 0 |
| 0 | 0 | 0 | 0 | 0 | 0 | 0 | 0 | 0 |
| 0 | 0 | 0 | 0 | 0 | 0 | 0 | 0 | 0 |
| 0 | 0 | 0 | 0 | 0 | 0 | 0 | 0 | 0 |
| 0 | 0 | 0 | 0 | 0 | 0 | 0 | 0 | 0 |
| 0 | 0 | 0 | 0 | 0 | 0 | 0 | 0 | 0 |
| 0 | 0 | 0 | 0 | 0 | 0 | 0 | 0 | 0 |
| 0 | 0 | 0 | 0 | 0 | 0 | 0 | 1 | 0 |
| 0 | 0 | 0 | 0 | 0 | 0 | 0 | 0 | 0 |
| 0 | 0 | 0 | 0 | 0 | 0 | 0 | 0 | 0 |
| 0 | 0 | 0 | 0 | 0 | 0 | 0 | 0 | 0 |
| 0 | 0 | 0 | 0 | 0 | 0 | 0 | 0 | 0 |
| 0 | 0 | 0 | 0 | 0 | 0 | 0 | 1 | 0 |
| 0 | 0 | 0 | 0 | 0 | 0 | 0 | 0 | 0 |
| 0 | 0 | 0 | 0 | 0 | 0 | 0 | 0 | 0 |
| 0 | 0 | 0 | 0 | 0 | 1 | 0 | 0 | 0 |
| 0 | 0 | 0 | 0 | 0 | 0 | 0 | 0 | 0 |
| 0 | 0 | 0 | 0 | 0 | 1 | 0 | 0 | 0 |
| 0 | 0 | 0 | 0 | 0 | 0 | 0 | 0 | 0 |
| 0 | 0 | 0 | 0 | 0 | 0 | 0 | 0 | 0 |
| 0 | 0 | 0 | 0 | 0 | 0 | 0 | 0 | 0 |
| 0 | 0 | 0 | 0 | 0 | 1 | 1 | 1 | 1 |
| 0 | 0 | 0 | 0 | 0 | 0 | 0 | 0 | 0 |
| 0 | 0 | 0 | 0 | 0 | 0 | 0 | 1 | 0 |
| 0 | 0 | 0 | 0 | 0 | 0 | 0 | 1 | 0 |
| 0 | 0 | 0 | 0 | 0 | 0 | 0 | 0 | 0 |
| 0 | 0 | 0 | 0 | 0 | 0 | 0 | 0 | 0 |
| 0 | 0 | 0 | 0 | 0 | 0 | 0 | 0 | 0 |
| 0 | 0 | 0 | 0 | 0 | 0 | 0 | 0 | 0 |
| 0 | 0 | 0 | 0 | 0 | 0 | 0 | 0 | 0 |
| 0 | 0 | 0 | 0 | 0 | 0 | 0 | 0 | 0 |
| 0 | 0 | 0 | 0 | 0 | 0 | 0 | 1 | 1 |

| | | | | | | | | |
|---|---|---|---|---|---|---|---|---|
| 0 | 0 | 0 | 0 | 0 | 0 | 0 | 0 | 0 |
| 0 | 0 | 0 | 0 | 0 | 0 | 0 | 0 | 0 |
| 0 | 0 | 0 | 0 | 0 | 0 | 0 | 0 | 0 |
| 0 | 0 | 0 | 0 | 0 | 0 | 0 | 1 | 0 |
| 0 | 0 | 0 | 0 | 0 | 0 | 0 | 1 | 0 |
| 0 | 0 | 0 | 0 | 0 | 0 | 0 | 0 | 0 |
| 0 | 0 | 0 | 0 | 0 | 0 | 0 | 0 | 0 |
| 0 | 0 | 0 | 0 | 0 | 0 | 0 | 1 | 0 |
| 0 | 0 | 0 | 0 | 0 | 0 | 0 | 0 | 0 |
| 0 | 0 | 0 | 0 | 0 | 0 | 0 | 1 | 0 |
| 0 | 0 | 0 | 0 | 0 | 0 | 0 | 0 | 0 |
| 0 | 0 | 0 | 0 | 0 | 0 | 1 | 0 | 0 |
| 0 | 0 | 0 | 0 | 0 | 0 | 0 | 0 | 0 |
| 0 | 0 | 0 | 0 | 0 | 0 | 0 | 0 | 0 |
| 0 | 0 | 0 | 0 | 0 | 0 | 0 | 0 | 0 |
| 0 | 0 | 0 | 0 | 0 | 0 | 0 | 0 | 0 |
| 0 | 0 | 0 | 0 | 0 | 0 | 0 | 0 | 0 |
| 0 | 0 | 0 | 0 | 0 | 0 | 0 | 0 | 0 |
| 0 | 0 | 0 | 0 | 0 | 0 | 0 | 0 | 0 |
| 0 | 0 | 0 | 0 | 0 | 0 | 0 | 0 | 1 |
| 0 | 0 | 0 | 0 | 0 | 0 | 0 | 0 | 0 |
| 0 | 0 | 0 | 0 | 0 | 0 | 0 | 1 | 0 |
| 0 | 0 | 0 | 0 | 0 | 0 | 0 | 0 | 0 |
| 0 | 0 | 0 | 0 | 0 | 0 | 0 | 0 | 0 |
| 0 | 0 | 0 | 0 | 1 | 1 | 0 | 1 | 0 |
| 0 | 0 | 0 | 0 | 0 | 0 | 0 | 0 | 0 |
| 0 | 0 | 0 | 0 | 0 | 0 | 0 | 0 | 0 |
| 0 | 0 | 0 | 0 | 0 | 0 | 0 | 0 | 0 |
| 0 | 0 | 0 | 0 | 0 | 0 | 0 | 1 | 0 |
| 0 | 0 | 0 | 0 | 0 | 0 | 0 | 0 | 0 |
| 0 | 0 | 0 | 0 | 0 | 0 | 0 | 0 | 0 |
| 0 | 0 | 0 | 0 | 0 | 0 | 0 | 1 | 0 |
| 0 | 0 | 0 | 0 | 0 | 0 | 0 | 0 | 0 |
| 0 | 0 | 0 | 0 | 0 | 0 | 0 | 0 | 0 |
| 0 | 0 | 0 | 0 | 0 | 0 | 0 | 0 | 0 |
| 0 | 0 | 0 | 0 | 1 | 1 | | | |
| 0 | 0 | 0 | 0 | 0 | 0 | 0 | 0 | 0 |
| 0 | 0 | 0 | 0 | 0 | 0 | 0 | 0 | 0 |
| 0 | 0 | 0 | 0 | 0 | 0 | 0 | 1 | 0 |
| 0 | 0 | 0 | 0 | 0 | 0 | 0 | 0 | 0 |
| 0 | 0 | 0 | 0 | 0 | 0 | 0 | 0 | 0 |
| 0 | 0 | 0 | 0 | 0 | 0 | 0 | 0 | 0 |
| 0 | 0 | 0 | 0 | 0 | 0 | 0 | 0 | 0 |
| 0 | 0 | 0 | 0 | 0 | 0 | 0 | 0 | 0 |
| 0 | 0 | 0 | 0 | 0 | 0 | 0 | 0 | 0 |
| 0 | 0 | 0 | 0 | 0 | 0 | | 0 | 0 |
| 0 | 0 | 0 | 0 | 0 | 0 | 0 | 0 | 0 |
| 0 | 0 | 0 | 0 | 0 | 0 | 0 | 0 | 0 |
| 0 | 0 | 0 | 0 | 0 | 0 | 0 | 0 | 1 |
| 0 | 0 | 0 | 0 | 1 | 0 | 0 | 1 | 0 |
| 0 | 0 | 0 | 0 | 0 | 0 | 0 | 1 | 0 |
| 0 | 0 | 0 | 0 | 0 | 1 | 0 | 1 | 0 |
| 0 | 0 | 0 | 0 | 0 | 0 | 0 | 0 | |
| 0 | 0 | 0 | 0 | 1 | 0 | 0 | 0 | 0 |
| 0 | 0 | 0 | 0 | 0 | 0 | 0 | 0 | 0 |
| 0 | 0 | 0 | 0 | 0 | 0 | 0 | 0 | 0 |
| 0 | 0 | 0 | 0 | 0 | 0 | 0 | 0 | 0 |
| 0 | 0 | 0 | 0 | 0 | 0 | 0 | 0 | 0 |
| 0 | 0 | 0 | 0 | 0 | 0 | 0 | 0 | 0 |

| | | | | | | | | |
|---|---|---|---|---|---|---|---|---|
| 0 | 0 | 0 | 0 | 0 | 0 | 0 | 0 | 0 |
| 0 | 0 | 0 | 0 | 0 | 0 | 0 | 1 | 0 |
| 0 | 0 | 0 | 0 | 0 | 0 | 0 | 1 | 0 |
| 0 | 0 | 0 | 0 | 0 | 0 | 0 | 0 | 0 |
| 0 | 0 | 0 | 0 | 0 | 0 | 0 | 0 | 0 |
| 0 | 0 | 0 | 0 | 0 | 0 | 0 | 0 | 0 |
| 0 | 0 | 0 | 0 | 0 | 0 | 0 | 0 | 0 |
| 0 | 0 | 0 | 0 | 0 | 0 | 0 | 0 | 0 |
| 0 | 0 | 0 | 0 | 0 | 0 | 0 | 0 | 0 |
| 0 | 0 | 0 | 0 | 0 | 0 | 0 | 1 | 0 |
| 0 | 0 | 0 | 0 | | 0 | 0 | 1 | 0 |
| 0 | 0 | 0 | 0 | 0 | 0 | 0 | 1 | 0 |
| 0 | 0 | 0 | 0 | 0 | 0 | 0 | 0 | 0 |
| 0 | 0 | 0 | 0 | 0 | 0 | 0 | 0 | 0 |
| 0 | 0 | 0 | 0 | 0 | | | | |
| 0 | 0 | 0 | 0 | 0 | 0 | 0 | 0 | 0 |
| 0 | 0 | 0 | 0 | 0 | 0 | 0 | 0 | 0 |
| 0 | 0 | 0 | 0 | 0 | 0 | 0 | 0 | 0 |
| 0 | 0 | 0 | 0 | 0 | 0 | 0 | 0 | 0 |
| 0 | 0 | 0 | 0 | 0 | 0 | 0 | 0 | 0 |
| 0 | 0 | 0 | 0 | 0 | 0 | 1 | 0 | 0 |
| 0 | 0 | 0 | 0 | 0 | 0 | 0 | 0 | 0 |
| 0 | 0 | 0 | 0 | 0 | 0 | 0 | 0 | 0 |
| 0 | 0 | 0 | 0 | 1 | 1 | 0 | 0 | 0 |
| 0 | 0 | 0 | 0 | 0 | 0 | 0 | 0 | 0 |
| 0 | 0 | 0 | 0 | 0 | 0 | 0 | 0 | 0 |
| 0 | 0 | 0 | 0 | 0 | 0 | 0 | 0 | 0 |
| 0 | 0 | 0 | 0 | 0 | 0 | 0 | 0 | 0 |
| 0 | 0 | 0 | 0 | 0 | 0 | 0 | 0 | 0 |
| 0 | 0 | 0 | 0 | 0 | 0 | 0 | 0 | 0 |
| 0 | 0 | 0 | 0 | 0 | 0 | 0 | 0 | 0 |
| 0 | 0 | 0 | 0 | 0 | 0 | 0 | 0 | 0 |
| 0 | 0 | 0 | 0 | 0 | 0 | 0 | 0 | 0 |
| 0 | 0 | 0 | 0 | 0 | 0 | 0 | 1 | 0 |
| 0 | 0 | 0 | 0 | 0 | 1 | 0 | 0 | 0 |
| 0 | 0 | 0 | 0 | 0 | 0 | 0 | 0 | 0 |
| 0 | 0 | 0 | 0 | 0 | 0 | 0 | 0 | 0 |
| 0 | 0 | 0 | 0 | 0 | 0 | 0 | 0 | 0 |
| 0 | 0 | 0 | 0 | 0 | 0 | 0 | 1 | 0 |
| 0 | 0 | 0 | 0 | 0 | 0 | 0 | 0 | 0 |
| 0 | 0 | 0 | 0 | 0 | 0 | 0 | 0 | 0 |
| 0 | 0 | 0 | 0 | 0 | 0 | 0 | 1 | 0 |
| 0 | 0 | 0 | 0 | 0 | 0 | 0 | 0 | 0 |
| 0 | 0 | 0 | 0 | 0 | 0 | 0 | 0 | 0 |
| 1 | 1 | 0 | 1 | 0 | 0 | 0 | 0 | 0 |
| 0 | 0 | 0 | 0 | 1 | 1 | 0 | 0 | 0 |
| 0 | 0 | 0 | 0 | 0 | 0 | 0 | 0 | 0 |
| 0 | 0 | 0 | 0 | 0 | 0 | 0 | 0 | 0 |
| 0 | 0 | 0 | 0 | 0 | 0 | 0 | 0 | 0 |
| 0 | 0 | 0 | 0 | 0 | 0 | 0 | 0 | 0 |
| 0 | 0 | 0 | 0 | 0 | 0 | 0 | 0 | 0 |
| 0 | 0 | 0 | 0 | 0 | 0 | 0 | 1 | 0 |
| 0 | 0 | 0 | 0 | 0 | 0 | 0 | 0 | 0 |
| 0 | 0 | 0 | 0 | 0 | 0 | 0 | 0 | 0 |
| 0 | 0 | 0 | 0 | 0 | 0 | 0 | 0 | 0 |
| 0 | 0 | 0 | 0 | 0 | 0 | 0 | 0 | 0 |
| 0 | 0 | 0 | 0 | 0 | 1 | 0 | 0 | 1 |
| 0 | 0 | 0 | 0 | 0 | 0 | 0 | 0 | 0 |
| 0 | 0 | 0 | 0 | 0 | 0 | 0 | 0 | 0 |

| | | | | | | | | |
|---|---|---|---|---|---|---|---|---|
| 0 | 0 | 0 | 0 | 0 | 0 | 0 | 0 | 0 |
| 0 | 0 | 0 | 0 | 0 | 0 | 0 | 0 | 0 |
| 0 | 0 | 0 | 0 | 0 | 0 | 0 | 0 | 0 |
| 0 | 0 | 0 | 0 | 0 | 0 | 0 | 0 | 0 |
| 0 | 0 | 0 | 0 | 0 | 0 | 0 | 0 | 0 |
| 0 | 0 | 0 | 0 | 0 | 0 | 1 | 0 | 0 |
| 0 | 0 | 0 | 0 | 0 | 0 | 0 | 0 | 0 |
| 0 | 0 | 0 | 0 | 0 | 0 | 0 | 0 | 0 |
| 0 | 0 | 0 | 0 | 0 | 0 | 0 | 0 | 0 |
| 0 | 0 | 0 | 0 | 0 | 0 | 0 | 0 | 0 |
| 0 | 0 | 0 | 0 | 0 | 0 | 0 | 0 | 0 |
| 0 | 0 | 0 | 0 | 0 | 0 | 0 | 0 | 0 |
| 0 | 0 | 0 | 0 | 0 | 0 | 0 | 0 | 0 |
| 0 | 0 | 0 | 0 | 0 | 0 | 0 | 0 | 0 |
| 0 | 0 | 0 | 0 | 0 | 0 | 0 | 0 | 0 |
| 0 | 0 | 0 | 0 | 0 | 0 | 0 | 0 | 0 |
| 0 | 0 | 0 | 0 | 0 | 0 | 0 | 0 | |
| 0 | 0 | 0 | 0 | 0 | 0 | 0 | 0 | 0 |
| 0 | 0 | 0 | 0 | 0 | 0 | 0 | 0 | 0 |
| 0 | 0 | 0 | 0 | 0 | 0 | 0 | 1 | 0 |
| 0 | 0 | 0 | 0 | 0 | 0 | 0 | 0 | 0 |
| 0 | 0 | 0 | 0 | 0 | 0 | 0 | 1 | 0 |
| 0 | 0 | 0 | 0 | 0 | 0 | 0 | 0 | 0 |
| 0 | 0 | 0 | 0 | 0 | 0 | 0 | 0 | 0 |
| 0 | 0 | 0 | 0 | 0 | 0 | 0 | 0 | 0 |
| 0 | 0 | 0 | 0 | 0 | 0 | 0 | 0 | 0 |
| 0 | 0 | 0 | 0 | 0 | 0 | 0 | 1 | 0 |
| 0 | 0 | 0 | 0 | 0 | 1 | 0 | 0 | 0 |
| 0 | 0 | 0 | 0 | 0 | 0 | 0 | 0 | 0 |
| 0 | 0 | 0 | 0 | 0 | 0 | 0 | 1 | 0 |
| 0 | 0 | 0 | 0 | 0 | 0 | 0 | 0 | 0 |
| 0 | 0 | 0 | 0 | 0 | 0 | 0 | 0 | 0 |
| 0 | 0 | 0 | 0 | 0 | 0 | 0 | 0 | 0 |
| 0 | 0 | 0 | 0 | 0 | 0 | 0 | 0 | 1 |
| 0 | 0 | 0 | 0 | 0 | 0 | 0 | 0 | 0 |
| 0 | 1 | 1 | 0 | 0 | 0 | 0 | 0 | 0 |
| 0 | 0 | 0 | 0 | 0 | 0 | 0 | 0 | 0 |
| 0 | 0 | 0 | 0 | 0 | 0 | 0 | 0 | 0 |
| 0 | 0 | 0 | 0 | 0 | 0 | 0 | 0 | 0 |
| 0 | 0 | 0 | 0 | 0 | 0 | 0 | 0 | 0 |
| 0 | 0 | 0 | 0 | 0 | 0 | 0 | 0 | 0 |
| 0 | 0 | 0 | 0 | 0 | 0 | 0 | 0 | 0 |
| 0 | 0 | 0 | 0 | 0 | 0 | 0 | 0 | 0 |
| 0 | 0 | 0 | 0 | 0 | 0 | 0 | 0 | 0 |
| 0 | 0 | 0 | 0 | 0 | 0 | 0 | 0 | 0 |
| 0 | 0 | 0 | 0 | 0 | 0 | 0 | 0 | 0 |
| 0 | 0 | 0 | 0 | 0 | 0 | 0 | 0 | 0 |
| 0 | 0 | 0 | 0 | 0 | 0 | 0 | 0 | 0 |
| 0 | 1 | 1 | 0 | 0 | 0 | 0 | 0 | 0 |
| 0 | 0 | 0 | 0 | 0 | 0 | 0 | 0 | 0 |
| 0 | 0 | 0 | 0 | 0 | 0 | 0 | 0 | 0 |
| 0 | 0 | 0 | 0 | 0 | 0 | 0 | 0 | 0 |
| 0 | 0 | 0 | 0 | 1 | 1 | 1 | 1 | 0 |
| 0 | 0 | 0 | 0 | 0 | 1 | 1 | 1 | 1 |
| 0 | 0 | 0 | 0 | 0 | 0 | 0 | 0 | 0 |
| 0 | 1 | 1 | 0 | 0 | 0 | 0 | 1 | 0 |
| 0 | 0 | 0 | 0 | 1 | 0 | 0 | 0 | 0 |
| 0 | 0 | 0 | 0 | 0 | 0 | 0 | 0 | 0 |
| 0 | 0 | 0 | 0 | 0 | 0 | 0 | 0 | 0 |

| | | | | | | | | |
|---|---|---|---|---|---|---|---|---|
| 0 | 1 | 1 | 0 | 0 | 0 | 0 | 0 | 0 |
| 0 | 0 | 0 | 0 | 0 | 0 | 0 | 0 | 0 |
| 0 | 0 | 0 | 0 | | | | | |
| 0 | 0 | 0 | 0 | | | | | |
| 0 | 0 | 0 | 0 | | | | | |
| 0 | 0 | 0 | 0 | | | | | |
| 0 | 0 | 0 | 0 | | | | | |
| 0 | 0 | 0 | 0 | | | | | |
| 0 | 0 | 0 | 0 | | | | | |
| 0 | 0 | 0 | 0 | | | | | |
| 0 | 0 | 0 | 0 | | | | | |
| 0 | 0 | 0 | 0 | | | | | |
| 0 | 0 | 0 | 0 | | | | | |
| 0 | 0 | 0 | 0 | | | | | |
| 0 | 0 | 0 | 0 | | | | | |
| 0 | 1 | 1 | 0 | | | | | |
| 0 | 0 | 0 | 0 | | | | | |
| 0 | 0 | 0 | 0 | | | | | |
| 0 | 0 | 0 | 0 | | | | | |
| 0 | 0 | 0 | 0 | | | | | |
| 0 | 0 | 0 | 0 | | | | | |
| 0 | 0 | 0 | 0 | | | | | |
| 0 | 0 | 0 | 0 | | | | | |
| 0 | 0 | 0 | 0 | | | | | |
| 0 | 0 | 0 | 0 | | | | | |
| 0 | 0 | 0 | 0 | | | | | |
| 0 | 1 | 1 | 0 | | | | | |
| 0 | 0 | 0 | 0 | | | | | |
| 0 | 0 | 0 | 0 | | | | | |
| 0 | 0 | 0 | 0 | | | | | |
| 0 | 0 | 0 | 0 | | | | | |
| 0 | 0 | 0 | 0 | | | | | |
| 0 | 0 | 0 | 0 | | | | | |
| 0 | 0 | 0 | 0 | | | | | |
| 0 | 0 | 0 | 0 | | | | | |
| 0 | 0 | 0 | 0 | | | | | |
| 0 | 0 | 0 | 0 | | | | | |
| 0 | 0 | 0 | 0 | | | | | |
| 0 | 0 | 0 | 0 | | | | | |
| 0 | 0 | 0 | 0 | | | | | |
| 0 | 0 | 0 | 0 | | | | | |
| 0 | 0 | 0 | 0 | | | | | |
| 0 | 0 | 0 | 0 | | | | | |
| 0 | 0 | 0 | 0 | | | | | |
| 0 | 0 | 0 | 0 | | | | | |
| 0 | 0 | 0 | 0 | | | | | |
| 0 | 0 | 0 | 0 | | | | | |
| 0 | 0 | 0 | 0 | | | | | |
| 0 | 0 | 0 | 0 | | | | | |
| 0 | 0 | 0 | 0 | | | | | |
| 0 | 0 | 0 | 0 | | | | | |
| 0 | 0 | 0 | 0 | | | | | |
| | | | | | | | | |
| 0 | 0 | 0 | 0 | | | | | |
| | | | | | | | | |
| 0 | 0 | 0 | 0 | | | | | |
| 0 | 0 | 0 | 0 | | | | | |
| | | | | | | | | |

| | | | | | | | | |
|---|---|---|---|---|---|---|---|---|
| 0 | 0 | 0 | 0 | | | | | |
| 0 | 0 | 0 | 0 | | | | | |
| 0 | 0 | 0 | 0 | | | | | |
| 0 | 0 | 0 | 0 | | | | | |
| | | | | | | | | |
| 0 | 0 | 0 | 0 | | | | | |
| 0 | 0 | 0 | 0 | | | | | |
| | | | | | | | | |
| 0 | 0 | 0 | 0 | | | | | |
| | | | | | | | | |
| 0 | 0 | 0 | 0 | | | | | |
| 0 | 0 | 0 | 0 | | | | | |
| 0 | 0 | 0 | 0 | | | | | |
| 0 | 0 | 0 | 0 | | | | | |
| | | | | | | | | |
| 0 | 0 | 0 | 0 | | | | | |
| 0 | 0 | 0 | 0 | | | | | |
| 0 | 0 | 0 | 0 | | | | | |
| 0 | 0 | 0 | 0 | | | | | |
| | | | | | | | | |
| | | | | | | | | |
| 0 | 0 | 0 | 0 | | | | | |
| 0 | 0 | 0 | 0 | | | | | |
| 0 | 0 | 0 | 0 | | | | | |
| 0 | 0 | 0 | 0 | | | | | |
| | | | | | | | | |
| | | | | | | | | |
| | | | | 0 | 1 | 1 | 0 | 1 |
| 0 | 0 | 0 | 0 | 0 | 0 | 0 | 0 | 1 |
| 0 | 0 | 0 | 0 | 0 | 0 | 0 | 0 | 0 |
| 0 | 0 | 0 | 0 | 0 | 0 | 0 | 0 | 0 |

| Språk | almotorisk | dning barnl | en på kogn. Påv | | | | | | |
|---|---|---|---|---|---|---|---|---|---|
| | | | | | | | | | |
| | | | | | | | | | |
| | | | | | | | | | |
| | | | | | | | | | |
| | | | | | | | | | |
| | | | | | | | | | |
| | | | | | | | | | |
| | | | | | | | | | |
| | | | | | | | | | |
| | | | | | | | | | |
| | | | | | | | | | |
| | | | | | | | | | |
| | | | | | | | | | |
| | | | | | | | | | |
| | | | | | | | | | |
| | | | | | | | | | |
| | | | | | | | | | |
| | | | | | | | | | |
| | | | | | | | | | |
| | | | | | | | | | |
| | | | | | | | | | |
| | | | | | | | | | |
| | | | | | | | | | |
| | | | | | | | | | |
| | | | | | | | | | |
| | | | | | | | | | |
| | | | | | | | | | |
| | | | | | | | | | |
| | | | | | | | | | |
| | | | | | | | | | |
| | | | | | | | | | |
| | | | | | | | | | |
| | | | | | | | | | |
| | | | | | | | | | |
| | | | | | | | | | |
| | | | | | | | | | |
| | | | | | | | | | |
| | | | | | | | | | |
| | | | | | | | | | |
| | | | | | | | | | |
| | | | | | | | | | |
| | | | | | | | | | |
| | | | | | | | | | |
| | | | | | | | | | |
| | | | | | | | | | |
| | | | | | | | | | |
| | | | | | | | | | |
| | | | | | | | | | |
| | | | | | | | | | |
| | | | | | | | | | |
| | | | | | | | | | |
| | | | | | | | | | |
| | | | | | | | | | |
| | | | | | | | | | |
| | | | | | | | | | |
| | | | | | | | | | |
| | | | | | | | | | |
| | | | | | | | | | |

| | | | | | | | | | |
|---|---|---|---|---|---|---|---|---|---|
| | | | | | | | | | |
| | | | | | | | | | |
| | | | | | | | | | |
| | | | | | | | | | |
| | | | | | | | | | |
| | | | | | | | | | |
| | | | | | | | | | |
| | | | | | | | | | |
| | | | | | | | | | |
| | | | | | | | | | |
| | | | | | | | | | |
| | | | | | | | | | |
| | | | | | | | | | |
| | | | | | | | | | |
| | | | | | | | | | |
| | | | | | | | | | |
| | | | | | | | | | |
| | | | | | | | | | |
| | | | | | | | | | |
| | | | | | | | | | |
| | | | | | | | | | |
| | | | | | | | | | |
| | | | | | | | | | |
| | | | | | | | | | |
| | | | | | | | | | |
| | | | | | | | | | |
| | | | | | | | | | |
| | | | | | | | | | |
| | | | | | | | | | |
| | | | | | | | | | |
| | | | | | | | | | |
| | | | | | | | | | |
| | | | | | | | | | |
| | | | | | | | | | |
| | | | | | | | | | |
| | | | | | | | | | |
| | | | | | | | | | |
| | | | | | | | | | |
| | | | | | | | | | |
| | | | | | | | | | |
| | | | | | | | | | |
| | | | | | | | | | |
| | | | | | | | | | |
| | | | | | | | | | |
| | | | | | | | | | |
| | | | | | | | | | |
| | | | | | | | | | |
| | | | | | | | | | |
| 0 | 0 | 0 | 0 | | | | | | |
| 0 | 0 | 0 | 0 | | | | | | |
| 0 | 0 | 0 | 0 | | | | | | |
| 0 | 0 | 0 | 0 | Mamma död | | | | | |
| 0 | 0 | 0 | 0 | | | | | | |
| 0 | 0 | 0 | 0 | | | | | | |
| 0 | 0 | 0 | 0 | | | | | | |
| 0 | 0 | 0 | 0 | | | | | | |
| 0 | 0 | 0 | 0 | | | | | | |
| 0 | 0 | 0 | 0 | | | | | | |

| | | | | |
|---|---|---|---|---|
| 0 | 0 | 0 | 0 | |
| 0 | 0 | 0 | 0 | |
| 0 | 0 | 0 | 0 | |
| 0 | 0 | 0 | 0 | |
| 0 | 0 | 0 | 0 | Syn 0.8 med HVOT |
| 0 | 0 | 0 | 0 | i 2015 dysleksi |
| 1 | 0 | 0 | 0 | 0,8 bilokulärt, ikke ren udtale |
| 0 | 0 | 0 | 0 | Syn 0.8 hö / 0.5 ve |
| 1 | 0 | 0 | 0 | Kan inte sige R |
| 0 | 0 | 0 | 0 | |
| 0 | 0 | 0 | 0 | |
| 0 | 0 | 0 | 0 | |
| 0 | 0 | 0 | 0 | |
| 0 | 0 | 0 | 0 | |
| 0 | 0 | 0 | 0 | Syn 0,65 vä, 0,8 hö |
| 0 | 0 | 0 | 0 | |
| 0 | 0 | 0 | 0 | |
| 0 | 0 | 0 | 0 | |
| 0 | 0 | 0 | 0 | |
| 0 | 0 | 0 | 0 | !! Hämtat från 5-årskontroll) |
| 0 | 0 | 0 | 0 | kan inte hoppe på et ben bilateralt |
| 0 | 0 | 1 | 0 | Kontakt med Danderyds sjukhus ögonklinik. Vet ej vilken diagnos |
| 0 | 0 | 0 | 0 | |
| 0 | 0 | 0 | 0 | Mangler at udfylde forståelse af instruktion. |
| 0 | 0 | 0 | 0 | |
| 0 | 0 | 0 | 0 | |
| 0 | 0 | 0 | 0 | |
| 1 | 1 | 1 | 1 | CP-skada pga asfyxi. Normal grav. Värksvag under förlossning. In |
| 0 | 0 | 0 | 0 | |
| 0 | 0 | 0 | 0 | |
| 0 | 0 | 0 | 0 | |
| 0 | 0 | 0 | 0 | |
| 0 | 0 | 0 | 0 | |
| 0 | 0 | 0 | 0 | |
| 0 | 0 | 0 | 0 | |
| 0 | 0 | 0 | 0 | |
| 0 | 0 | 0 | 0 | |
| 0 | 0 | 0 | 0 | |
| 0 | 0 | 0 | 0 | |
| 0 | 0 | 0 | 0 | |
| 0 | 0 | 0 | 0 | |
| 0 | 0 | 1 | 0 | Remiss pga systol. Blåsljud, men ingen uppföljning |
| 0 | 0 | 0 | 0 | Skelen |
| 0 | 0 | 0 | 1 | Omogen pennfattning. Ritar ej huvudfoting. |
| 0 | 0 | 0 | 0 | |
| 1 | 0 | 0 | 1 | Stockig kontroll, medvirker ej bra, vil selv bestemme |
| 0 | 0 | 0 | 0 | |
| 0 | 0 | 0 | 0 | |
| 0 | 0 | 0 | 0 | |
| 0 | 0 | 0 | 0 | |
| 0 | 0 | 0 | 0 | |
| 0 | 0 | 0 | 0 | |
| 0 | 0 | 0 | 0 | |
| 0 | 0 | 0 | 0 | |
| 0 | 0 | 0 | 0 | |
| 0 | 0 | 0 | 0 | |
| 0 | 0 | 0 | 0 | |
| 0 | 0 | 0 | 0 | Syn vä, 0,8; hö 0,8 |
| 0 | 0 | 0 | 0 | |

| | | | | | |
|---|---|---|---|---|---|
| 0 | 0 | 0 | 0 | | |
| 0 | 1 | 0 | 0 | muligvis, kramper som tolkes som feberkramper | |
| 0 | 0 | 0 | 0 | | |
| 0 | 0 | 0 | 0 | | |
| 0 | 0 | 1 | 0 | Utretts för obstruktiv bronkit vid 2 mån ålder | |
| 0 | 0 | 0 | 0 | Ritar inte som han burde | |
| 0 | 0 | 0 | 0 | | |
| 0 | 0 | 0 | 0 | | |
| 1 | 1 | 1 | 1 | Barnklinik Lindköping, kramper för och ved 4 års alder. Henvist og | |
| 0 | 0 | 0 | 0 | | |
| 0 | 0 | 0 | 0 | | |
| 0 | 0 | 0 | 0 | | |
| 0 | 0 | 0 | 0 | | |
| 0 | 0 | 0 | 0 | | |
| 0 | 0 | 0 | 0 | | |
| 0 | 0 | 0 | 0 | | |
| 0 | 0 | 0 | 0 | | |
| 1 | 0 | 0 | 0 | Kan inte sige R | |
| 0 | 0 | 0 | 0 | | |
| 0 | 0 | 0 | 0 | | |
| 0 | 0 | 0 | 0 | Remiss till ortoped pga hälta. Bedömdes normalt, ingen åtgärd. | |
| 0 | 0 | 0 | 0 | | |
| 0 | 0 | 0 | 0 | | |
| 0 | 0 | 0 | 0 | | |
| 0 | 0 | 0 | 0 | | |
| 0 | 0 | 0 | 0 | | |
| 0 | 0 | 0 | 0 | | |
| 0 | 0 | 0 | 0 | | |
| 0 | 0 | 0 | 0 | | |
| 0 | 0 | 0 | 0 | | |
| 0 | 0 | 0 | 0 | | |
| 0 | 0 | 0 | 0 | Ifylddt af foräldre | |
| 0 | 0 | 0 | 0 | | |
| 0 | 0 | 0 | 0 | | |
| 0 | 0 | 0 | 0 | Syn 0,5 hö, 0,8 vä | |
| 0 | 0 | 0 | 0 | | |
| 0 | 0 | 0 | 0 | | |
| 0 | 0 | 0 | 0 | | |
| 0 | 0 | 0 | 0 | | |
| 0 | 0 | 0 | 0 | Vä 0,5, hö 0,65 | |
| 0 | 0 | 0 | 0 | | |
| 0 | 0 | 0 | 0 | | |
| 1 | 0 | 1 | 1 | Utretts neu | Autism |
| 1 | 0 | 0 | 0 | kan inte udtale S | |
| 0 | 0 | 0 | 0 | kan inte ritta som forventet | |
| 0 | 0 | 0 | 0 | | |
| 0 | 0 | 0 | 0 | | |
| 0 | 0 | 0 | 0 | | |
| 1 | 1 | 1 | 1 | Charge syndrom, arveligt, ikke asphyxi | |
| 1 | 0 | 0 | 0 | Stammar, henvist til logopäd | |
| 0 | 0 | 0 | 0 | Översynt, går på kontrol | |
| 0 | 0 | 0 | 0 | Syn 0,65 h | Vid 5-årskontroll: Remiss till Syn-ortoptist |
| 0 | 0 | 0 | 0 | | |
| 0 | 0 | 0 | 0 | | |
| 0 | 0 | 0 | 0 | | |
| 0 | 0 | 0 | 0 | | |
| 0 | 0 | 0 | 0 | Läs och skriv vanskeligheder (mammas udtalelse), BVC 5 års kon | |
| 0 | 0 | 0 | 0 | | |
| 0 | 0 | 0 | 0 | Nedsat syn och nedsat hörsel, ikke udredt på barnklinik | |

| | | | | |
|---|---|---|---|---|
| 0 | 0 | 0 | 0 | Från 5 1/2 års kontroll!!) |
| 0 | 0 | 0 | 0 | |
| 0 | 0 | 0 | 0 | |
| 0 | 0 | 0 | 0 | Glasögen H 0,5; V 0,8 |
| 0 | 0 | 1 | 0 | Tidligere kontakt med barnmedicinsk mott ang tilväkst |
| 0 | 0 | 0 | 0 | |
| 0 | 0 | 0 | 0 | |
| 0 | 0 | 1 | 0 | thyroidea, tager Levaxin |
| 0 | 0 | 0 | 0 | |
| 0 | 0 | 0 | 0 | Hö och ve öje 0,65 |
| 0 | 0 | 0 | 0 | |
| 0 | 0 | 0 | 0 | |
| 0 | 0 | 0 | 0 | |
| 0 | 0 | 1 | 0 | Kraftig overvægt |
| 0 | 0 | 0 | 0 | |
| 0 | 0 | 0 | 0 | svärt med at läse/skrive |
| 1 | 0 | 0 | 0 | Kan inte formulere sig, kender man inte barnet forståes ej hvad de |
| 0 | 0 | 0 | 0 | |
| 0 | 0 | 0 | 0 | |
| 1 | 0 | 0 | 0 | Ören inflamation, drän. Kan inte sige S |
| 0 | 0 | 0 | 0 | |
| 0 | 0 | 0 | 0 | Syn 0,65 bägge ögon |
| 0 | 0 | 0 | 0 | |
| 0 | 0 | 0 | 0 | |
| 1 | 0 | 1 | | Udredt pga forsenet udvikling |
| 0 | 0 | 0 | 0 | |
| 0 | 0 | 0 | 0 | |
| 0 | 0 | 0 | 0 | |
| 0 | 0 | 0 | 0 | 0,65 bilateralt, dålig hærsel ved kontrol på ØNH |
| 0 | 0 | 0 | 0 | |
| 0 | 0 | 0 | 0 | |
| 0 | 0 | 0 | 0 | Nedsatt syn ena ögat -> glasögon. |
| 0 | 0 | 0 | 0 | |
| 1 | 0 | 0 | 0 | Nasalt tal, remiss til logoped |
| 0 | 0 | 0 | 0 | |
| | 1 | 1 | | CP |
| 0 | 0 | 1 | 0 | Allergi ägg mjölk |
| 0 | 0 | 0 | 0 | |
| 0 | 0 | 0 | 0 | Syn 0,8 bilat |
| 0 | 0 | 1 | 0 | Obstipation |
| 0 | 0 | 0 | 0 | |
| 0 | 0 | 0 | 0 | |
| 0 | 0 | 0 | 0 | |
| 0 | 0 | 0 | 0 | |
| 1 | 0 | 0 | 0 | Kan inte udtale R |
| | | | | |
| 0 | 0 | 0 | 0 | |
| 1 | 0 | 0 | 0 | Kan inte ud tale RS, remiss til logoped |
| 1 | 0 | 0 | 0 | |
| 1 | 0 | 1 | 0 | kan inte hoppe, nedsat syn ve æga, sen utviklet sprog, overvikt |
| 1 | 0 | 0 | 1 | Syn: amblyopi hö öga. Språk: språkstörning ljudsystemet enl logop |
| 0 | 0 | 0 | 1 | Ritar ej huvudfoting. Syn 0,8 hö och vä |
| 0 | 0 | 0 | 0 | Hörsel ej kollat |
| 0 | 0 | 0 | 0 | Problem med hoppa på ett ben. |
| 0 | 0 | 0 | 0 | |
| 1 | 0 | 0 | 0 | kan inte sige R lyd |
| 0 | 0 | 0 | 0 | |
| 0 | 0 | 0 | 0 | |
| 0 | 0 | 0 | 0 | |

| | | | | | | | | | |
|---|---|---|---|---|---|---|---|---|---|
| 0 | 0 | 0 | 0 | | | | | | |
| 0 | 0 | 0 | 0 | Syn 0,5 båda ögon | | | | | |
| 0 | 0 | 0 | 0 | ogonmott | | | | | |
| 0 | 0 | 0 | 0 | | | | | | |
| 0 | 0 | 0 | 0 | | | | | | |
| 0 | 0 | 0 | 0 | | | | | | |
| 0 | 0 | 0 | 0 | | | | | | |
| 0 | 0 | 0 | 0 | | | | | | |
| 0 | 0 | 0 | 0 | | | | | | |
| 0 | 0 | 0 | 0 | Occulär albinism | | | | | |
| 0 | 0 | 0 | 0 | Syn, HVOT 0.65 | | | | | |
| 0 | 0 | 0 | 0 | Syn HVOT 0,65 bägge ögon, skelning. Språk: r till j. | | | | | |
| 1 | 0 | 0 | 0 | Kan inte sige R ljud, logoped | | | | | |
| 0 | 0 | 0 | 0 | | | | | | |
| 0 | | | | Ingen anmärkninger ved skolundersökning. | | | | | |
| 0 | 0 | 0 | 0 | | | | | | |
| 0 | 0 | 0 | 0 | | | | | | |
| 0 | 0 | 0 | 1 | Behöver mkt stöd i skolarbetet | | | | | |
| 0 | 0 | 0 | 0 | | | | | | |
| 0 | 0 | 0 | 0 | | | | | | |
| 0 | 0 | 0 | 0 | Astma | | | | | |
| 0 | 0 | 0 | 0 | | | | | | |
| 0 | 0 | 0 | 0 | | | | | | |
| 0 | 0 | 0 | 0 | | | | | | |
| 0 | 0 | 0 | 0 | | | | | | |
| 0 | 0 | 0 | 0 | | | | | | |
| 0 | 0 | 0 | 0 | Pronation båda fötterna, remiss till ortoped | | | | | |
| 0 | 0 | 0 | 0 | | | | | | |
| 0 | 0 | 0 | 0 | | | | | | |
| 0 | 0 | 0 | 0 | Skolen finder inte journalen, spörgeskema er udfyldt af föräldrene | | | | | |
| 1 | 0 | 0 | 0 | Svärt at sige R | | | | | |
| 0 | 0 | 0 | 0 | | | | | | |
| 0 | 0 | 0 | 0 | | | | | | |
| 0 | 0 | 0 | 0 | Bilat exofori | | | | | |
| 1 | 1 | 0 | 1 | Finmotorik: växlar hö- och vänsterhänt. Språk: sluddrar vid sponta | | | | | |
| 0 | 0 | 0 | 0 | | | | | | |
| 0 | 0 | 0 | 0 | | | | | | |
| 0 | 0 | 0 | 0 | | | | | | |
| 0 | 0 | 0 | 0 | Syn 0,65 vä, 0,8 hö | | | | | |
| 0 | 0 | 0 | 0 | | | | | | |
| 0 | 0 | 0 | 0 | | | | | | |
| 0 | 0 | 0 | 0 | Syn 0,65 vä, 0,8 hö | | | | | |
| 0 | 0 | 0 | 0 | | | | | | |
| 0 | 0 | 0 | 0 | | | | | | |
| 0 | 0 | 0 | 0 | | | | | | |
| 1 | 0 | 1 | 0 | forsänet utvikling, språkstörning og motorisk sväriheter. | | | | | |
| 0 | 0 | 0 | 0 | | | | | | |
| 1 | 0 | 0 | 0 | Kan ikke sige R | | | | | |
| 0 | 0 | 0 | 0 | | | | | | |
| 0 | 0 | 1 | 0 | Misstänkt mjölkallergi/laktosintolerans. Astma. | | | | | |
| 0 | 0 | 0 | 0 | | | | | | |
| 0 | 0 | 0 | 1 | 0,8 bilateralt, kan inte se forskel på farver. | | | | | |
| 0 | 0 | 0 | 0 | | | | | | |
| 0 | 0 | 0 | 0 | | | | | | |
| 0 | 0 | 0 | 0 | | | | | | |
| 0 | 0 | 0 | 0 | | | | | | |
| 0 | 0 | 0 | 0 | kan ikke perler på tråd, nedsat hærelse på hø øre gælder alle frek | | | | | |
| 0 | 0 | 0 | 0 | | | | | | |
| 0 | 0 | 0 | 0 | | | | | | |

| | | | | |
|---|---|---|---|---|
| 0 | 0 | 0 | 0 | |
| 0 | 0 | 0 | 0 | |
| 0 | 0 | 0 | 0 | |
| 0 | 0 | 0 | 0 | |
| 0 | 0 | 0 | 0 | (Svårt förstå instruktioner, pga att svenska är andraspråk) |
| 1 | 0 | 0 | 0 | Talepedagog, logped |
| 0 | 0 | 0 | 0 | |
| 0 | 0 | 0 | 0 | |
| 0 | 0 | 0 | 0 | |
| 0 | 0 | 0 | 0 | |
| 1 | 0 | 0 | 1 | Henvist til BUP/BUNN för 4 års alder. |
| 0 | 0 | 0 | 0 | |
| 0 | 0 | 0 | 0 | |
| 0 | 0 | 0 | 0 | |
| 0 | 0 | 0 | 0 | |
| 0 | 0 | 0 | 0 | |
| 0 | 0 | 0 | 0 | |
| 0 | 0 | 0 | 0 | |
| 0 | 0 | 1 | 0 | Remiss pga circumoral cyanos. Bedömdes perifert betingad |
| 0 | 0 | 1 | 0 | Syn: hö 0,8, vä 0,65 Språk: kan ej uttala r-ljud Utredning: astma |
| 0 | 0 | 1 | 0 | Eksem. Allergi? |
| 0 | 0 | 0 | 0 | syn vä 0,8; hö 0,8 |
| 0 | 0 | 0 | 0 | |
| 0 | 0 | 0 | 0 | |
| 0 | 0 | 0 | 0 | |
| 0 | 0 | 0 | 0 | !! Hämtat från 5-årskontroll) |
| 0 | 0 | 1 | 0 | Visus 0,8 bilat, udredning pga dålig tilväkst + kronisk obstipation |
| 1 | 0 | 0 | 0 | kan ej rita huvedfoting, utydeligt tal, henvist til logoped |
| 0 | 0 | 0 | 0 | |
| 0 | 0 | 0 | 0 | 0,8 höjre och vänstre |
| 0 | 0 | 0 | 0 | Normal efter foräldre udsagn, skema udfyldt efter 5års kontrol |
| 0 | 0 | 0 | 0 | |
| 0 | 0 | 0 | 0 | |
| 0 | 0 | 0 | 0 | Kronisk mellemöre inflamation |
| 0 | 0 | 0 | 0 | |
| 0 | 0 | 0 | 0 | |
| 0 | 0 | 0 | 0 | |
| 0 | 0 | 0 | 0 | |
| 0 | 0 | 0 | 0 | |
| 0 | 0 | 0 | 0 | |
| 0 | 0 | 1 | 0 | Språk: säger inte r. Utr barnklinik: UVI, rotavirus. |
| 0 | 0 | 0 | 0 | |
| 0 | 0 | 0 | 0 | |
| 0 | 0 | 0 | 0 | |
| 0 | 0 | 1 | 1 | Svärt for koncentration og side stille, har også epilepsi |
| 0 | 0 | 0 | 0 | |
| 0 | 0 | 1 | 0 | Udredning pga af dålig tilväkst. |
| 0 | 0 | 0 | 0 | |
| 0 | 0 | 0 | 0 | |
| 0 | 0 | 0 | 0 | |
| 0 | 0 | 1 | 0 | Utretts för kramper vid 9 mån ålder - utan anmärkn |
| 0 | 0 | 0 | 0 | |
| 1 | 0 | 1 | 1 | Grov: balansprobl. Fin: ritar ej. Tal: sen talutv. |
| 1 | 0 | 1 | 1 | Problem med finmotorik och samleken. Skeler, hörer dårligt, stam |
| 0 | 0 | 0 | 0 | |
| 0 | 0 | 1 | 1 | Visus bilat 6,65; Remis pga ADHA, kan inte fokusere i store grupp |
| 0 | 1 | 1 | 0 | CP skada, har svårt stå länge og hoppe med det venstre ben. |
| 0 | 0 | 0 | 0 | |
| 1 | 0 | 0 | 0 | Försenad talutv. |

| | | | | | | | | | |
|---|---|---|---|---|---|---|---|---|---|
| 0 | 0 | 0 | 0 | | | | | | |
| 0 | 0 | 0 | 0 | | | | | | |
| | | | | | | | | | |
| | | | | | | | | | |
| | | | | | | | | | |
| | | | | | | | | | |
| | | | | | | | | | |
| | | | | | | | | | |
| | | | | | | | | | |
| | | | | | | | | | |
| | | | | | | | | | |
| | | | | | | | | | |
| | | | | | | | | | |
| | | | | | | | | | |
| | | | | | | | | | |
| | | | | | | | | | |
| | | | | | | | | | |
| | | | | | | | | | |
| | | | | | | | | | |
| | | | | | | | | | |
| | | | | | | | | | |
| | | | | | | | | | |
| | | | | | | | | | |
| | | | | | | | | | |
| | | | | | | | | | |
| | | | | | | | | | |
| | | | | | | | | | |
| | | | | Skolen svarer ej | | | | | |
| | | | | | | | | | |
| | | | | | | | | | |
| | | | | | | | | | |
| | | | | | | | | | |
| | | | | | | | | | |
| | | | | | | | | | |
| | | | | | | | | | |
| | | | | | | | | | |
| | | | | | | | | | |
| | | | | | | | | | |
| | | | | Avlidet, genupplivning afbruts | | | | | |
| | | | | Vil inte deltage | | | | | |
| | | | | Avlider, hjärtmissbildning | | | | | |
| | | | | Emigreret til Forende Arabemiranten | | | | | |
| | | | | Emigreret til Australien | | | | | |
| | | | | Vil inte deltage | | | | | |
| | | | | Sekretess | | | | | |
| | | | | fraflyttet, findes inte i folkbokföring | | | | | |
| | | | | Emigreret Uganda | | | | | |
| | | | | Fraflyttet, findes inte i folkbokföring | | | | | |
| | | | | Vil inte deltage | | | | | |
| | | | | Flyttet til UK | | | | | |
| | | | | Mener ikke hun har deltaget, derfor udesluttet | | | | | |
| | | | | Fraflyttat | | | | | |
| | | | | Journalen kan inte fremskaffes | | | | | |
| | | | | Transposition, usikker på utfald | | | | | |
| | | | | Emigreret, Frankrig | | | | | |
| | | | | Död | | | | | |
| | | | | Emigreret, USA | | | | | |
| | | | | Vil inte medverka | | | | | |
| | | | | Flyttet til IRAK | | | | | |

| | | | | | | | | | |
|---|---|---|---|---|---|---|---|---|---|
| | | | | Emigreret, Schweiz | | | | | |
| | | | | Fraflyttet, findes inte i folkbokföring | | | | | |
| | | | | Fraflytttet | | | | | |
| | | | | Journal finns ej | | | | | |
| | | | | Journal finns ej | | | | | |
| | | | | BVC journal finns ej | | | | | |
| | | | | Journal finns ej | | | | | |
| | | | | Emigreret | | | | | |
| | | | | Avlidat | | | | | |
| | | | | Emigeret til UK | | | | | |
| | | | | Emigeret til UK | | | | | |
| | | | | Emigreret til US | | | | | |
| | | | | Mor har opgivet forkert personnr | | | | | |
| | | | | Journal kan inte findes | | | | | |
| | | | | Skydd personlighed | | | | | |
| | | | | Fraflyttet | | | | | |
| | | | | Fejlagtigt personnr på mor | | | | | |
| | | | | Vil inte medverka | | | | | |
| | | | | Fraflyttet | | | | | |
| | | | | Udrejst, Somalia | | | | | |
| | | | | Avlider, pga ukendt syndrom | | | | | |
| | | | | Fraflyttet | | | | | |
| | | | | Emigreret til Tyrkiet | | | | | |
| | | | | Fejagtigt personnr på mor | | | | | |
| | | | | Fraflyttet | | | | | |
| | | | | | | | | | |
| | | | | | | | | | |
| 1 | 1 | 1 | | dålig balans, kan inte [illegible]riat, forstår int instruktioner, udredt for autism | | | | | |
| 0 | 0 | 1 | 0 | Sensorineural hörselsnedsätning | | | | | |
| 0 | 0 | 0 | 0 | | | | | | |
| 0 | 0 | 0 | 0 | | | | | | |

| | | | | | | | | | |
|---|---|---|---|---|---|---|---|---|---|
| | | | | | | | | | |
| | | | | | | | | | |
| | | | | | | | | | |
| | | | | | | | | | |
| | | | | | | | | | |
| | | | | | | | | | |
| | | | | | | | | | |
| | | | | | | | | | |
| | | | | | | | | | |
| | | | | | | | | | |
| | | | | | | | | | |
| | | | | | | | | | |
| | | | | | | | | | |
| | | | | | | | | | |
| | | | | | | | | | |
| | | | | | | | | | |
| | | | | | | | | | |
| | | | | | | | | | |
| | | | | | | | | | |
| | | | | | | | | | |
| | | | | | | | | | |
| | | | | | | | | | |
| | | | | | | | | | |
| | | | | | | | | | |
| | | | | | | | | | |
| | | | | | | | | | |
| | | | | | | | | | |
| | | | | | | | | | |
| | | | | | | | | | |
| | | | | | | | | | |
| | | | | | | | | | |
| | | | | | | | | | |
| | | | | | | | | | |
| | | | | | | | | | |
| | | | | | | | | | |
| | | | | | | | | | |
| | | | | | | | | | |
| | | | | | | | | | |
| | | | | | | | | | |
| | | | | | | | | | |
| | | | | | | | | | |
| | | | | | | | | | |
| | | | | | | | | | |
| | | | | | | | | | |
| | | | | | | | | | |
| | | | | | | | | | |
| | | | | | | | | | |
| | | | | | | | | | |
| | | | | | | | | | |
| | | | | | | | | | |
| | | | | | | | | | |
| | | | | | | | | | |
| | | | | | | | | | |
| | | | | | | | | | |
| | | | | | | | | | |
| | | | | | | | | | |
| | | | | | | | | | |
| | | | | | | | | | |

| | | | | | | | | | |
|---|---|---|---|---|---|---|---|---|---|
| | | | | | | | | | |
| | | | | | | | | | |
| | | | | | | | | | |
| | | | | | | | | | |
| | | | | | | | | | |
| | | | | | | | | | |
| | | | | | | | | | |
| | | | | | | | | | |
| | | | | | | | | | |
| | | | | | | | | | |
| | | | | | | | | | |
| | | | | | | | | | |
| | | | | | | | | | |
| | | | | | | | | | |
| | | | | | | | | | |
| | | | | | | | | | |
| | | | | | | | | | |
| | | | | | | | | | |
| | | | | | | | | | |
| | | | | | | | | | |
| | | | | | | | | | |
| | | | | | | | | | |
| | | | | | | | | | |
| | | | | | | | | | |
| | | | | | | | | | |
| | | | | | | | | | |
| | | | | | | | | | |
| gen 4-årskontroll på BVC | | | | | | | | | |
| | | | | | | | | | |
| | | | | | | | | | |
| | | | | | | | | | |
| | | | | | | | | | |
| | | | | | | | | | |
| | | | | | | | | | |
| | | | | | | | | | |
| | | | | | | | | | |
| | | | | | | | | | |
| | | | | | | | | | |
| | | | | | | | | | |
| | | | | | | | | | |
| | | | | | | | | | |
| | | | | | | | | | |
| | | | | | | | | | |
| | | | | | | | | | |
| | | | | | | | | | |
| | | | | | | | | | |
| | | | | | | | | | |
| | | | | | | | | | |
| | | | | | | | | | |
| | | | | | | | | | |
| | | | | | | | | | |
| | | | | | | | | | |
| | | | | | | | | | |
| | | | | | | | | | |
| | | | | | | | | | |
| | | | | | | | | | |
| | | | | | | | | | |
| | | | | | | | | | |
| | | | | | | | | | |

| | | | | | | | | | |
|---|---|---|---|---|---|---|---|---|---|
| | | | | | | | | | |
| | | | | | | | | | |
| | | | | | | | | | |
| | | | | | | | | | |
| | | | | | | | | | |
| | | | | | | | | | |
| | | | | | | | | | |
| | | | | | | | | | |
| så til psykolog. | | | | | | | | | |
| | | | | | | | | | |
| | | | | | | | | | |
| | | | | | | | | | |
| | | | | | | | | | |
| | | | | | | | | | |
| | | | | | | | | | |
| | | | | | | | | | |
| | | | | | | | | | |
| | | | | | | | | | |
| | | | | | | | | | |
| | | | | | | | | | |
| | | | | | | | | | |
| | | | | | | | | | |
| | | | | | | | | | |
| | | | | | | | | | |
| | | | | | | | | | |
| | | | | | | | | | |
| | | | | | | | | | |
| | | | | | | | | | |
| | | | | | | | | | |
| | | | | | | | | | |
| | | | | | | | | | |
| | | | | | | | | | |
| | | | | | | | | | |
| | | | | | | | | | |
| | | | | | | | | | |
| | | | | | | | | | |
| | | | | | | | | | |
| | | | | | | | | | |
| | | | | | | | | | |
| | | | | | | | | | |
| | | | | | | | | | |
| | | | | | | | | | |
| | | | | | | | | | |
| | | | | | | | | | |
| | | | | | | | | | |
| | | | | | | | | | |
| | | | | | | | | | |
| | | | | | | | | | |
| | | | | | | | | | |
| | | | | | | | | | |
| | | | | | | | | | |
| | | | | | | | | | |
| | | | | | | | | | |
| | | | | | | | | | |
| | | | | | | | | | |
| trol | | | | | | | | | |
| | | | | | | | | | |
| | | | | | | | | | |

| | | | | | | | | | |
|---|---|---|---|---|---|---|---|---|---|
| | | | | | | | | | |
| | | | | | | | | | |
| | | | | | | | | | |
| | | | | | | | | | |
| | | | | | | | | | |
| | | | | | | | | | |
| | | | | | | | | | |
| | | | | | | | | | |
| | | | | | | | | | |
| | | | | | | | | | |
| | | | | | | | | | |
| | | | | | | | | | |
| | | | | | | | | | |
| | | | | | | | | | |
| | | | | | | | | | |
| | | | | | | | | | |
| r siges | | | | | | | | | |
| | | | | | | | | | |
| | | | | | | | | | |
| | | | | | | | | | |
| | | | | | | | | | |
| | | | | | | | | | |
| | | | | | | | | | |
| | | | | | | | | | |
| | | | | | | | | | |
| | | | | | | | | | |
| | | | | | | | | | |
| | | | | | | | | | |
| | | | | | | | | | |
| | | | | | | | | | |
| | | | | | | | | | |
| | | | | | | | | | |
| | | | | | | | | | |
| | | | | | | | | | |
| | | | | | | | | | |
| | | | | | | | | | |
| | | | | | | | | | |
| | | | | | | | | | |
| | | | | | | | | | |
| | | | | | | | | | |
| | | | | | | | | | |
| | | | | | | | | | |
| | | | | | | | | | |
| | | | | | | | | | |
| | | | | | | | | | |
| | | | | | | | | | |
| | | | | | | | | | |
| | | | | | | | | | |
| | | | | | | | | | |
| | | | | | | | | | |
| ed | | | | | | | | | |
| | | | | | | | | | |
| | | | | | | | | | |
| | | | | | | | | | |
| | | | | | | | | | |
| | | | | | | | | | |
| | | | | | | | | | |
| | | | | | | | | | |
| | | | | | | | | | |

ntal. Centralmot: kognitivt, kan ej färger.

venser

mer, remiteret til BUP og logopäd

er

| | | | | | | | | | |
|---|---|---|---|---|---|---|---|---|---|
| | | | | | | | | | |
| | | | | | | | | | |
| | | | | | | | | | |
| | | | | | | | | | |
| | | | | | | | | | |
| | | | | | | | | | |
| | | | | | | | | | |
| | | | | | | | | | |
| | | | | | | | | | |
| | | | | | | | | | |
| | | | | | | | | | |
| | | | | | | | | | |
| | | | | | | | | | |
| | | | | | | | | | |
| | | | | | | | | | |
| | | | | | | | | | |
| | | | | | | | | | |
| | | | | | | | | | |
| | | | | | | | | | |
| | | | | | | | | | |
| | | | | | | | | | |
| | | | | | | | | | |
| | | | | | | | | | |
| | | | | | | | | | |
| | | | | | | | | | |
| | | | | | | | | | |
| | | | | | | | | | |
| | | | | | | | | | |
| | | | | | | | | | |
| | | | | | | | | | |
| | | | | | | | | | |
| | | | | | | | | | |
| | | | | | | | | | |
| | | | | | | | | | |
| | | | | | | | | | |
| | | | | | | | | | |
| | | | | | | | | | |
| | | | | | | | | | |
| | | | | | | | | | |
| | | | | | | | | | |
| | | | | | | | | | |
| | | | | | | | | | |
| | | | | | | | | | |
| | | | | | | | | | |
| | | | | | | | | | |
| | | | | | | | | | |
| | | | | | | | | | |
| | | | | | | | | | |
| | | | | | | | | | |
| | | | | | | | | | |
| | | | | | | | | | |
| | | | | | | | | | |
| | | | | | | | | | |
| | | | | | | | | | |
| | | | | | | | | | |
| | | | | | | | | | |
| | | | | | | | | | |
| | | | | | | | | | |

| | | | | | | | | | |
|---|---|---|---|---|---|---|---|---|---|
| | | | | | | | | | |
| | | | | | | | | | |
| | | | | | | | | | |
| | | | | | | | | | |
| | | | | | | | | | |
| | | | | | | | | | |
| | | | | | | | | | |
| | | | | | | | | | |
| | | | | | | | | | |
| | | | | | | | | | |
| | | | | | | | | | |
| | | | | | | | | | |
| | | | | | | | | | |
| | | | | | | | | | |
| | | | | | | | | | |
| | | | | | | | | | |
| | | | | | | | | | |
| | | | | | | | | | |
| | | | | | | | | | |
| | | | | | | | | | |
| | | | | | | | | | |
| | | | | | | | | | |
| | | | | | | | | | |
| | | | | | | | | | |
| | | | | | | | | | |
| | | | | | | | | | |
| | | | | | | | | | |
| | | | | | | | | | |
| | | | | | | | | | |
| | | | | | | | | | |

| | | | | | | | |
|---|---|---|---|---|---|---|---|
| | | | | | | | |
| | | | | | | | |
| | | | | | | | |
| | | | | | | | |
| | | | | | | | |
| | | | | | | | |
| | | | | | | | |
| | | | | | | | |
| | | | | | | | |
| | | | | | | | |
| | | | | | | | |
| | | | | | | | |
| | | | | | | | |
| | | | | | | | |
| | | | | | | | |
| | | | | | | | |
| | | | | | | | |
| | | | | | | | |
| | | | | | | | |
| | | | | | | | |
| | | | | | | | |
| | | | | | | | |
| | | | | | | | |
| | | | | | | | |
| | | | | | | | |
| | | | | | | | |
| | | | | | | | |
| | | | | | | | |
| | | | | | | | |
| | | | | | | | |
| | | | | | | | |
| | | | | | | | |
| | | | | | | | |
| | | | | | | | |
| | | | | | | | |
| | | | | | | | |
| | | | | | | | |
| | | | | | | | |
| | | | | | | | |
| | | | | | | | |
| | | | | | | | |
| | | | | | | | |
| | | | | | | | |
| | | | | | | | |
| | | | | | | | |
| | | | | | | | |
| | | | | | | | |
| | | | | | | | |
| | | | | | | | |
| | | | | | | | |
| | | | | | | | |
| | | | | | | | |
| | | | | | | | |
| | | | | | | | |
| | | | | | | | |
| | | | | | | | |
| | | | | | | | |
| | | | | | | | |

| | | | | | | | |
|---|---|---|---|---|---|---|---|
| | | | | | | | |
| | | | | | | | |
| | | | | | | | |
| | | | | | | | |
| | | | | | | | |
| | | | | | | | |
| | | | | | | | |
| | | | | | | | |
| | | | | | | | |
| | | | | | | | |
| | | | | | | | |
| | | | | | | | |
| | | | | | | | |
| | | | | | | | |
| | | | | | | | |
| | | | | | | | |
| | | | | | | | |
| | | | | | | | |
| | | | | | | | |
| | | | | | | | |
| | | | | | | | |
| | | | | | | | |
| | | | | | | | |
| | | | | | | | |
| | | | | | | | |
| | | | | | | | |
| | | | | | | | |
| | | | | | | | |
| | | | | | | | |
| | | | | | | | |
| | | | | | | | |
| | | | | | | | |
| | | | | | | | |
| | | | | | | | |
| | | | | | | | |
| | | | | | | | |
| | | | | | | | |
| | | | | | | | |
| | | | | | | | |
| | | | | | | | |
| | | | | | | | |
| | | | | | | | |
| | | | | | | | |
| | | | | | | | |
| | | | | | | | |
| | | | | | | | |
| | | | | | | | |
| | | | | | | | |
| | | | | | | | |
| | | | | | | | |
| | | | | | | | |
| | | | | | | | |
| | | | | | | | |
| | | | | | | | |
| | | | | | | | |
| | | | | | | | |
| | | | | | | | |
| | | | | | | | |

| | | | | | | | |
|---|---|---|---|---|---|---|---|
| | | | | | | | |
| | | | | | | | |
| | | | | | | | |
| | | | | | | | |
| | | | | | | | |
| | | | | | | | |
| | | | | | | | |
| | | | | | | | |
| | | | | | | | |
| | | | | | | | |
| | | | | | | | |
| | | | | | | | |
| | | | | | | | |
| | | | | | | | |
| | | | | | | | |
| | | | | | | | |
| | | | | | | | |
| | | | | | | | |
| | | | | | | | |
| | | | | | | | |
| | | | | | | | |
| | | | | | | | |
| | | | | | | | |
| | | | | | | | |
| | | | | | | | |
| | | | | | | | |
| | | | | | | | |
| | | | | | | | |
| | | | | | | | |
| | | | | | | | |
| | | | | | | | |
| | | | | | | | |
| | | | | | | | |
| | | | | | | | |
| | | | | | | | |
| | | | | | | | |
| | | | | | | | |
| | | | | | | | |
| | | | | | | | |
| | | | | | | | |
| | | | | | | | |
| | | | | | | | |
| | | | | | | | |
| | | | | | | | |
| | | | | | | | |
| | | | | | | | |
| | | | | | | | |
| | | | | | | | |
| | | | | | | | |
| | | | | | | | |
| | | | | | | | |
| | | | | | | | |
| | | | | | | | |
| | | | | | | | |
| | | | | | | | |
| | | | | | | | |
| | | | | | | | |
| | | | | | | | |

| | | | | | | | |
|---|---|---|---|---|---|---|---|
| | | | | | | | |
| | | | | | | | |
| | | | | | | | |
| | | | | | | | |
| | | | | | | | |
| | | | | | | | |
| | | | | | | | |
| | | | | | | | |
| | | | | | | | |
| | | | | | | | |
| | | | | | | | |
| | | | | | | | |
| | | | | | | | |
| | | | | | | | |
| | | | | | | | |
| | | | | | | | |
| | | | | | | | |
| | | | | | | | |
| | | | | | | | |
| | | | | | | | |
| | | | | | | | |
| | | | | | | | |
| | | | | | | | |
| | | | | | | | |
| | | | | | | | |
| | | | | | | | |
| | | | | | | | |
| | | | | | | | |
| | | | | | | | |
| | | | | | | | |
| | | | | | | | |
| | | | | | | | |
| | | | | | | | |
| | | | | | | | |
| | | | | | | | |
| | | | | | | | |
| | | | | | | | |
| | | | | | | | |
| | | | | | | | |
| | | | | | | | |
| | | | | | | | |
| | | | | | | | |
| | | | | | | | |
| | | | | | | | |
| | | | | | | | |
| | | | | | | | |
| | | | | | | | |
| | | | | | | | |
| | | | | | | | |
| | | | | | | | |
| | | | | | | | |
| | | | | | | | |
| | | | | | | | |
| | | | | | | | |
| | | | | | | | |
| | | | | | | | |
| | | | | | | | |
| | | | | | | | |
| | | | | | | | |

| | | | | | | | |
|---|---|---|---|---|---|---|---|
| | | | | | | | |
| | | | | | | | |
| | | | | | | | |
| | | | | | | | |
| | | | | | | | |
| | | | | | | | |
| | | | | | | | |
| | | | | | | | |
| | | | | | | | |
| | | | | | | | |
| | | | | | | | |
| | | | | | | | |
| | | | | | | | |
| | | | | | | | |
| | | | | | | | |
| | | | | | | | |
| | | | | | | | |
| | | | | | | | |
| | | | | | | | |
| | | | | | | | |
| | | | | | | | |
| | | | | | | | |
| | | | | | | | |
| | | | | | | | |
| | | | | | | | |
| | | | | | | | |
| | | | | | | | |
| | | | | | | | |
| | | | | | | | |
| | | | | | | | |
| | | | | | | | |
| | | | | | | | |
| | | | | | | | |
| | | | | | | | |
| | | | | | | | |
| | | | | | | | |
| | | | | | | | |
| | | | | | | | |
| | | | | | | | |
| | | | | | | | |
| | | | | | | | |
| | | | | | | | |
| | | | | | | | |
| | | | | | | | |
| | | | | | | | |
| | | | | | | | |
| | | | | | | | |
| | | | | | | | |
| | | | | | | | |
| | | | | | | | |
| | | | | | | | |
| | | | | | | | |
| | | | | | | | |
| | | | | | | | |
| | | | | | | | |
| | | | | | | | |
| | | | | | | | |
| | | | | | | | |

| | | | | | | | |
|---|---|---|---|---|---|---|---|
| | | | | | | | |
| | | | | | | | |
| | | | | | | | |
| | | | | | | | |
| | | | | | | | |
| | | | | | | | |
| | | | | | | | |
| | | | | | | | |
| | | | | | | | |
| | | | | | | | |
| | | | | | | | |
| | | | | | | | |
| | | | | | | | |
| | | | | | | | |
| | | | | | | | |
| | | | | | | | |
| | | | | | | | |
| | | | | | | | |
| | | | | | | | |
| | | | | | | | |
| | | | | | | | |
| | | | | | | | |
| | | | | | | | |
| | | | | | | | |
| | | | | | | | |
| | | | | | | | |
| | | | | | | | |
| | | | | | | | |
| | | | | | | | |
| | | | | | | | |
| | | | | | | | |
| | | | | | | | |
| | | | | | | | |
| | | | | | | | |
| | | | | | | | |
| | | | | | | | |
| | | | | | | | |
| | | | | | | | |
| | | | | | | | |
| | | | | | | | |
| | | | | | | | |
| | | | | | | | |
| | | | | | | | |
| | | | | | | | |
| | | | | | | | |
| | | | | | | | |
| | | | | | | | |
| | | | | | | | |
| | | | | | | | |
| | | | | | | | |
| | | | | | | | |
| | | | | | | | |
| | | | | | | | |
| | | | | | | | |
| | | | | | | | |
| | | | | | | | |
| | | | | | | | |
| | | | | | | | |

| | | | | | | | |
|---|---|---|---|---|---|---|---|
| | | | | | | | |
| | | | | | | | |
| | | | | | | | |
| | | | | | | | |
| | | | | | | | |
| | | | | | | | |
| | | | | | | | |
| | | | | | | | |
| | | | | | | | |
| | | | | | | | |
| | | | | | | | |
| | | | | | | | |
| | | | | | | | |
| | | | | | | | |
| | | | | | | | |
| | | | | | | | |
| | | | | | | | |
| | | | | | | | |
| | | | | | | | |
| | | | | | | | |
| | | | | | | | |
| | | | | | | | |
| | | | | | | | |
| | | | | | | | |
| | | | | | | | |
| | | | | | | | |
| | | | | | | | |
| | | | | | | | |
| | | | | | | | |
| | | | | | | | |
| | | | | | | | |
| | | | | | | | |
| | | | | | | | |
| | | | | | | | |
| | | | | | | | |
| | | | | | | | |
| | | | | | | | |
| | | | | | | | |
| | | | | | | | |
| | | | | | | | |
| | | | | | | | |
| | | | | | | | |
| | | | | | | | |
| | | | | | | | |
| | | | | | | | |
| | | | | | | | |
| | | | | | | | |
| | | | | | | | |
| | | | | | | | |
| | | | | | | | |
| | | | | | | | |
| | | | | | | | |
| | | | | | | | |
| | | | | | | | |
| | | | | | | | |
| | | | | | | | |
| | | | | | | | |
| | | | | | | | |

| | | | | | | | |
|---|---|---|---|---|---|---|---|
| | | | | | | | |
| | | | | | | | |
| | | | | | | | |
| | | | | | | | |
| | | | | | | | |
| | | | | | | | |
| | | | | | | | |
| | | | | | | | |
| | | | | | | | |
| | | | | | | | |
| | | | | | | | |
| | | | | | | | |
| | | | | | | | |
| | | | | | | | |
| | | | | | | | |
| | | | | | | | |
| | | | | | | | |
| | | | | | | | |
| | | | | | | | |
| | | | | | | | |
| | | | | | | | |
| | | | | | | | |
| | | | | | | | |
| | | | | | | | |
| | | | | | | | |
| | | | | | | | |
| | | | | | | | |
| | | | | | | | |
| | | | | | | | |
| | | | | | | | |
| | | | | | | | |
| | | | | | | | |
| | | | | | | | |
| | | | | | | | |
| | | | | | | | |
| | | | | | | | |
| | | | | | | | |
| | | | | | | | |
| | | | | | | | |
| | | | | | | | |
| | | | | | | | |
| | | | | | | | |
| | | | | | | | |
| | | | | | | | |
| | | | | | | | |
| | | | | | | | |
| | | | | | | | |
| | | | | | | | |
| | | | | | | | |
| | | | | | | | |
| | | | | | | | |
| | | | | | | | |
| | | | | | | | |
| | | | | | | | |
| | | | | | | | |
| | | | | | | | |
| | | | | | | | |
| | | | | | | | |
| | | | | | | | |

| | | | | | | | |
|---|---|---|---|---|---|---|---|
| | | | | | | | |
| | | | | | | | |
| | | | | | | | |
| | | | | | | | |
| | | | | | | | |
| | | | | | | | |
| | | | | | | | |
| | | | | | | | |
| | | | | | | | |
| | | | | | | | |
| | | | | | | | |
| | | | | | | | |
| | | | | | | | |
| | | | | | | | |
| | | | | | | | |
| | | | | | | | |
| | | | | | | | |
| | | | | | | | |
| | | | | | | | |
| | | | | | | | |
| | | | | | | | |
| | | | | | | | |
| | | | | | | | |
| | | | | | | | |
| | | | | | | | |
| | | | | | | | |
| | | | | | | | |
| | | | | | | | |
| | | | | | | | |
| | | | | | | | |
| | | | | | | | |
| | | | | | | | |
| | | | | | | | |
| | | | | | | | |
| | | | | | | | |
| | | | | | | | |
| | | | | | | | |
| | | | | | | | |
| | | | | | | | |
| | | | | | | | |
| | | | | | | | |
| | | | | | | | |
| | | | | | | | |
| | | | | | | | |
| | | | | | | | |
| | | | | | | | |
| | | | | | | | |
| | | | | | | | |
| | | | | | | | |
| | | | | | | | |
| | | | | | | | |
| | | | | | | | |
| | | | | | | | |
| | | | | | | | |
| | | | | | | | |
| | | | | | | | |
| | | | | | | | |
| | | | | | | | |

| | | | | | | | |
|---|---|---|---|---|---|---|---|
| | | | | | | | |
| | | | | | | | |
| | | | | | | | |
| | | | | | | | |
| | | | | | | | |
| | | | | | | | |
| | | | | | | | |
| | | | | | | | |
| | | | | | | | |
| | | | | | | | |
| | | | | | | | |
| | | | | | | | |
| | | | | | | | |
| | | | | | | | |
| | | | | | | | |
| | | | | | | | |
| | | | | | | | |
| | | | | | | | |
| | | | | | | | |
| | | | | | | | |
| | | | | | | | |
| | | | | | | | |
| | | | | | | | |
| | | | | | | | |
| | | | | | | | |
| | | | | | | | |
| | | | | | | | |
| | | | | | | | |
| | | | | | | | |
| | | | | | | | |
| | | | | | | | |
| | | | | | | | |
| | | | | | | | |
| | | | | | | | |
| | | | | | | | |
| | | | | | | | |
| | | | | | | | |
| | | | | | | | |
| | | | | | | | |
| | | | | | | | |
| | | | | | | | |
| | | | | | | | |
| | | | | | | | |
| | | | | | | | |
| | | | | | | | |
| | | | | | | | |
| | | | | | | | |
| | | | | | | | |
| | | | | | | | |
| | | | | | | | |
| | | | | | | | |
| | | | | | | | |
| | | | | | | | |
| | | | | | | | |
| | | | | | | | |
| | | | | | | | |
| | | | | | | | |
| | | | | | | | |
| | | | | | | | |

| | | | | | | | |
|---|---|---|---|---|---|---|---|
| | | | | | | | |
| | | | | | | | |
| | | | | | | | |
| | | | | | | | |
| | | | | | | | |
| | | | | | | | |
| | | | | | | | |
| | | | | | | | |
| | | | | | | | |
| | | | | | | | |
| | | | | | | | |
| | | | | | | | |
| | | | | | | | |
| | | | | | | | |
| | | | | | | | |
| | | | | | | | |
| | | | | | | | |
| | | | | | | | |
| | | | | | | | |
| | | | | | | | |
| | | | | | | | |
| | | | | | | | |
| | | | | | | | |
| | | | | | | | |
| | | | | | | | |
| | | | | | | | |
| | | | | | | | |
| | | | | | | | |
| | | | | | | | |
| | | | | | | | |
